# Drug‐Induced Raynaud's Phenomenon and Underlying Mechanism: A Disproportionality Analysis From the World Health Organization Pharmacovigilance Database

**DOI:** 10.1002/art.43442

**Published:** 2026-02-03

**Authors:** Alex Hlavaty, Loubna Dari, Jean‐Luc Cracowski, Matthieu Roustit, Charles Khouri

**Affiliations:** ^1^ Pharmacovigilance Unit Grenoble Alpes University Hospital, University Grenoble Alpes F‐38000 Grenoble France; ^2^ University Grenoble Alpes, INSERM U1300 HP2 Grenoble France; ^3^ Department of Vascular Medicine Bordeaux University Hospital Bordeaux France; ^4^ Bordeaux Population Health, INSERM U1219 Bordeaux France; ^5^ University Grenoble Alpes, INSERM CIC1406, Grenoble Alpes University Hospital Grenoble France

## Abstract

**Objectives:**

The aim of this study is to generate hypotheses about unknown drugs associated with the onset or worsening of Raynaud's phenomenon (RP) and to explore their potential pathophysiologic mechanisms through a mixed disproportionality/clustering analysis from the World Health Organization (WHO) pharmacovigilance database.

**Methods:**

Using the WHO pharmacovigilance database, we identified cases using the Medical Dictionary for Regulatory Activities Preferred Term “Raynaud's phenomenon,” and we excluded all Individual Case Safety Reports (ICSRs) associated with at least one drug used in RP treatment. To estimate signals of disproportionate reporting (SDR), we calculated information component (IC) values (IC_LB_ >0 deemed significant). We performed several sensitivity analyses to assess the robustness of the results. We evaluated and prioritized the plausibility of signals according to expert review of cases characteristics, robustness of results, and pharmacological hypotheses. Lastly, to explore pathophysiologic mechanisms, we used drug target extraction from DrugBank and a clustering method to identify similar patterns of adverse events reporting.

**Results:**

We included 4,430 ICSRs of RP in our analysis. We found 124 significant SDRs in the primary analysis, of which 52 SDRs were consistent across all sensitivity analyses, and 16 were considered probable after signal evaluation and prioritization, including amphetamine‐like, antimigraine drugs, antineoplastics drugs, and dopaminergic agonists. Most of the targets involved were 5‐HT1A receptors, sodium‐dependent noradrenaline transporters, and beta‐1 and beta‐2 adrenergic receptors. Cluster analyses yielded inconsistent results according to the method used.

**Conclusion:**

This study allowed us to identify robust safety signals (such as solriamfetol, tyrosine kinase inhibitors, and calcitonin gene‐related peptide inhibitors) for drugs associated with RP and potential implicated pathophysiologic mechanisms.

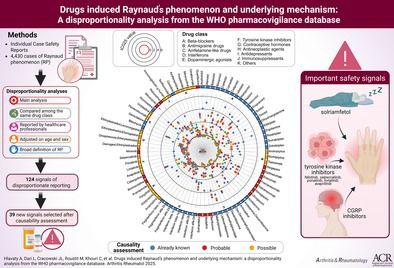

## Graphical abstract

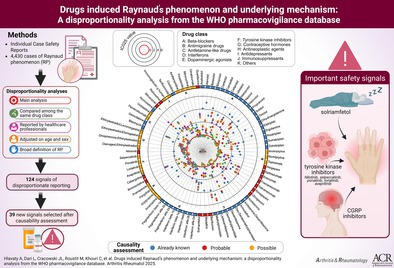

## INTRODUCTION

Raynaud's phenomenon (RP) is a condition characterized by skin color changes (pallor, cyanosis, and reactive hyperemia) of the extremities, mainly fingers or toes, triggered by cold or emotional stress.^1^ RP is classified into primary and secondary subtypes. On the one hand, primary RP is relatively common, affecting 2% to 12% of the general population, and represents the idiopathic form of the syndrome.^2^ On the other hand, secondary RP is associated with various conditions (particularly autoimmune connective tissue disease such as systemic sclerosis), exposures, or drugs.^3^ Although primary RP is a benign condition, secondary forms may lead to peripheral ischemia, digital ulcers, and necrosis. Medications identified as potentially inducing or aggravating RP include estrogens, nonselective beta blockers, vasoconstrictors (eg, ergotamine, nicotine, sympathomimetic drugs), and drugs used for the treatment of attention‐deficit/hyperactivity disorder (eg, atomoxetine, lisdexamfetamine).^4^ Yet, many other drugs are suspected in case reports or based on a common pharmacological mechanism. Moreover, new signals have recently emerged particularly with antimigraine calcitonin gene‐related peptide (CGRP) inhibitors or tyrosine kinase inhibitors (TKIs).^5^, ^6^

We sought to conduct a systematic analysis of suspected adverse events reported in the World Health Organization (WHO) pharmacovigilance database to identify potential new associations and characterize their spectrum, pathophysiology, and relevant clinical characteristics.

## METHODS

We reported this disproportionality analysis according to the REporting of A Disproportionality analysis for drUg Safety signal detection using Individual Case Safety Reports (ICSRs) in PharmacoVigilance (READUS‐PV) guideline.^7^, ^8^ The protocol of the study was preregistered on OSF (https://osf.io/4mqch/), and the READUS‐PV checklist is available in Supplementary Table 1. Because we used retrospective de‐identified data in this study, ethical approval and written informed consent were not required.

The data that support the findings of this study are available on request from VigiBase. The data are not publicly available due to privacy or ethical restrictions. However, all codes are available on request from ahlavaty@chu-grenoble.fr.

### Quantitative analysis of the WHO pharmacovigilance database

#### Data source and population

We extracted all ICSRs of RP in VigiBase from its inception until October 2024. In VigiBase, events are coded using the international Medical Dictionary for Regulatory Activities (MedDRA) terminology, which is hierarchically structured from Lowest Level Terms to System Organ Classes. We used the specific MedDRA Preferred Term “Raynaud's Phenomenon” to identify ICSRs of interest with all drugs considered as suspect or interacting.

We excluded from our analysis all ICSRs with at least one drug recommended in RP‐ and systemic sclerosis–related digital ulcers: calcium channel blockers, PDE5 inhibitors, endothelin receptors agonists, angiotensin receptor blockers, angiotensin‐converting enzyme inhibitors, alpha blockers, and intravenous prostanoids.^9^, ^10^

#### Variables

The following characteristics of ICSRs were extracted: country of primary source, reporting year, reporter type, and severity of the adverse event (defined based on the recording of one of the following outcomes: death, life‐threatening inpatient hospitalization or prolongation of existing hospitalization, persistent or significant disability or incapacity, congenital anomaly, or otherwise medically significant condition), patient age and sex, and time to onset. ICSRs for which the date was missing for drug initiation or onset of adverse event were ignored when calculating the time to onset of the reaction. If the date was not complete, we replaced it by setting the day in the middle of the month (if there is no day) or in the middle of the year (July 2, if there is no month and no day); when completion led to a negative time to onset, data were excluded.

#### Disproportionality analysis

The analyses were performed using a Bayesian disproportionality method the “Bayesian neural network method” developed by the Uppsala Monitoring Centre research team, which displays the best sensitivity and specificity among disproportionality analyses particularly for rare events.^11^, ^12^ In the primary analysis, the comparator group was composed by all remaining ICSRs in the database. The threshold used in this study for defining a significant signal of disproportionate reporting (SDR) was a lower boundary of the 95% credibility interval of the information component (IC_LB_) superior to 0.^11^, ^13^

#### Sensitivity analysis

We performed several sensitivity analyses to evaluate the robustness of SDRs. Information component values were re‐estimated by (1) restricting only to cases reported by health care professionals, hypothesizing that the level of plausibility is higher in these cases; (2) modifying the background/comparator group in using cases pertaining to the third level of the Anatomical Therapeutic Chemical classification instead of the whole database to account for indication bias and disease related confounding factors; (3) adjusting by Mantel‐Haenszel stratification the IC estimates by sex and age to limit confounding by these factors^3^, ^9^; and (4) using a broader definition of RP using a dedicated MedDRA collection of Preferred Terms “Raynaud's Phenomenon,” “Peripheral ischemia,” and “Chilblains” to account for variability in symptoms coding.

### Signal evaluation and prioritization

To identify previous awareness of associations between RP and drugs with a significant SDR, we used the Micromedex database; we retrieved data from the US Food and Drug Administration (FDA) Drug Safety Communications, Pharmacovigilance Risk Assessment Committee, FDA and European Medicines Agency (EMA) Summary of Product Characteristics (SmPc), and published literature reviews.^14^

Moreover, we summarized the key features of the ICSRs supporting the significant SDRs: age, sex, time to onset, dechallenge/rechallenge, concomitant drugs, and underlying disease. We used cross‐referenced databases (DrugBank and UniProt) to identify pharmacological and pathophysiologic hypotheses linking identified drugs and RP.

Based on the disproportionality estimates in the primary analysis, the robustness of the finding in sensitivity analyses, the cases‐by‐case analysis, and the pharmacological plausibility of the associations, all SDR were then independently and blindly assessed for plausibility by two senior experts in pharmacovigilance, vascular disease, and pharmacology (CK and LD).

Drugs identified in our searches on “previous awareness” described above were classified as “already described” (ie, very likely). Other suspected drugs were classified as “probable” or “possible” according to the level of plausibility. Drugs with uncertain associations were classified as “unlikely.”^15^ Discrepancies were resolved through discussion among authors.

### Network and consensus clustering

We performed two clustering methods to identify a pattern of co‐reported symptoms along with RP in hypothesizing that different pathophysiologic mechanisms may provoke heterogenous clinical symptoms associated with RP.

We therefore performed separate network clustering analyses for the four main pharmacological classes associated with RP (beta blockers, antimigraine drugs, amphetamine‐like drugs, and TKIs). In the network clustering, we previously selected only the Preferred Terms that showed a significant SDR using an event‐event disproportionality analysis. This analysis consists, among the same drug class, of comparing the adverse events co‐reported with RP to all other adverse events. Then, network clustering was performed by combining two methods to estimate association between Preferred Terms, Ising's model, and positive pointwise mutual information (PPMI) as described by Fusaroli et al.^16^ All statistical analyses were performed in R^17^ and Python.^18^, ^19^, ^20^

## RESULTS

### Description of cases

**Figure 1:**
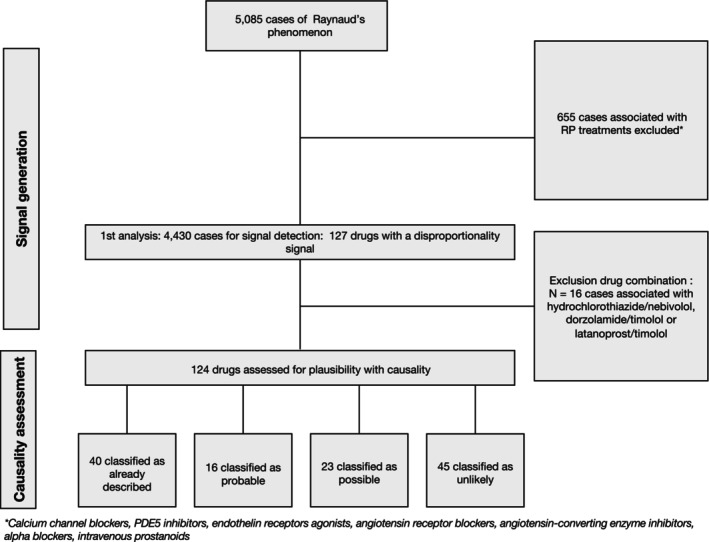
Flowchart of the population of cases selected included in disproportionality analyses and plausibility assessment of signals of disproportionate reporting. RP, Raynaud's phenomenon.

In October 2024, 5,085 ICSRs of RP were reported in the WHO pharmacovigilance database. After exclusion of ICSRs associated with RP‐ or systemic sclerosis–related digital ulcers, 4,430 cases were included in the analysis (Figure 1). Cases were mainly reported in the United States (n = 2005, 45.3%), followed by France (n = 464, 10.5%) and the United Kingdom (n = 426, 9.6%). Cases were reported primarily by health care professionals (n = 2,601, 58.7%). The mean age ± SD of included patients was 47.4 years ± 19.0 years, and there were 3,142 (70.9%) concerned women.

### Primary and sensitivity disproportionality analyses

Among the 843 suspect and interacting drugs retrieved in ICSRs reporting RP, 127 displayed a significant disproportionality signal in the primary analysis. The strongest SDRs were associated with lisdexamfetamine (IC_LB_ = 5.47, n = 188), methylphenidate (IC_LB_ = 4.48, n = 217), dexamfetamine (IC_LB_ = 4.46, n = 35), and atomoxetine (IC_LB_ = 3.89, n = 76).

**Figure 2:**
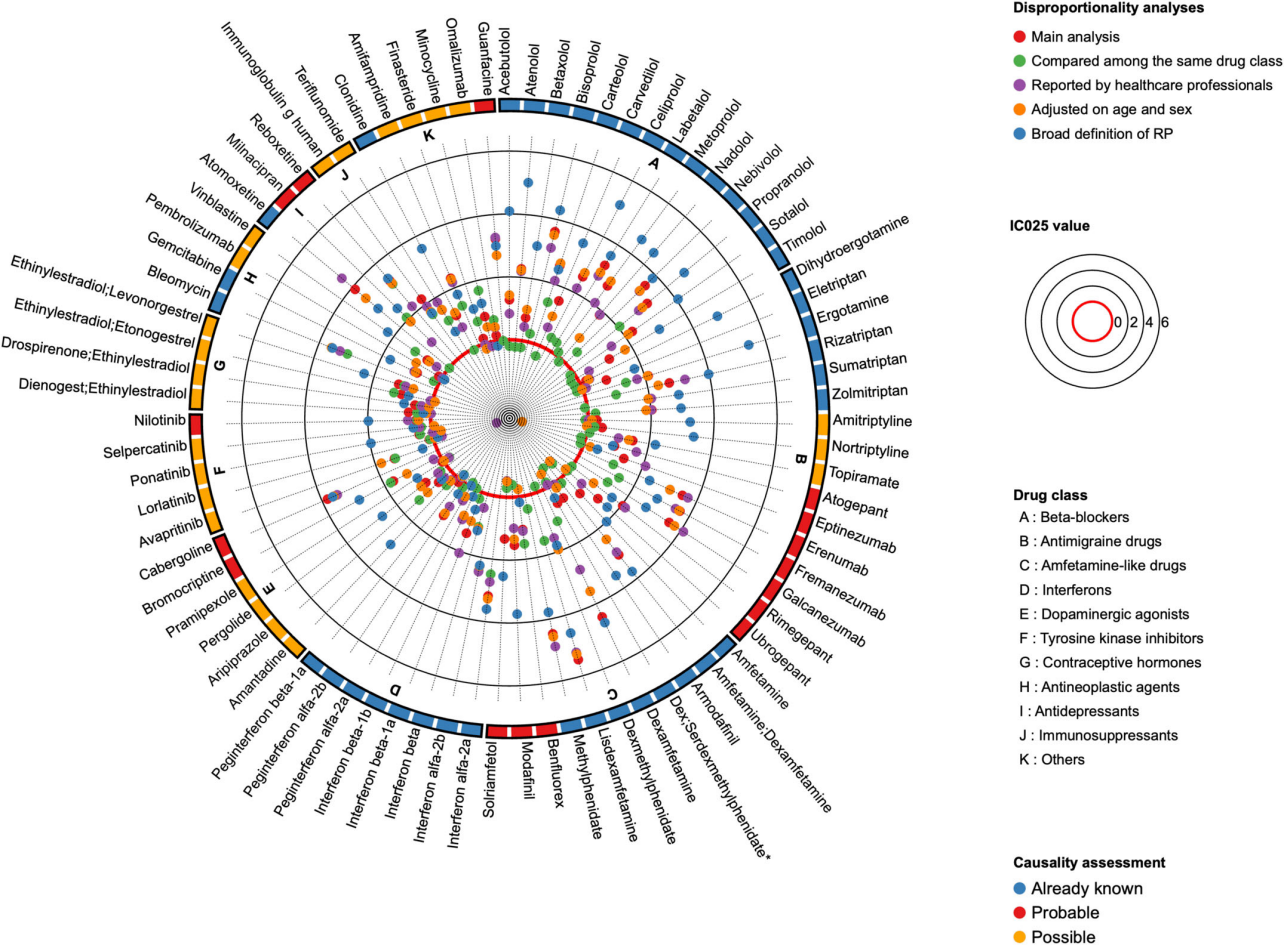
Circular dot plot presenting the results of disproportionality analysis and causality assessment for drugs associated with Raynaud's phenomenon in the WHO pharmacovigilance database. Each dot displays the results of main and sensitivity disproportionality analysis (lower bound of 95% credibility interval of Information Component). *Dexmethylphenidate; serdexmethylphenidate. IC, information component; RP, Raynaud's phenomenon.

After excluding three combined drugs (hydrochlorothiazide/nebivolol, dorzolamide/timolol and latanoprost/timolol), 124 drugs were assessed for plausibility. Among the latter, disproportionality signals remained consistently significant for 52 drugs in all sensitivity analyses (Figure 2). Descriptions of the key features of cases summarized by drug are available in Supplementary Table 2.

### Signal evaluation and prioritization

After retrieving the Micromedex database, FDA safety communications, and FDA and EMA SmPCs and literature reviews on RP, 40 drugs with a significant SDR were classified as “already described,” as shown in Figure 2. Based on the characteristics of the cases, potential pharmacological mechanisms, robustness of SDRs in sensitivity analyses, and review of the preexisting literature, we assessed the plausibility of the identified signals (presented in Supplementary Table 2). Forty‐five were classified as “unlikely,” 23 as possible, and 16 as “probable.” Results of disproportionality analyses and characteristics of cases are presented in Supplementary Table 3. The main reasons for considering drug role to be unlikely were the presence of an indication bias (eg, drug indicated in a disease known to cause RP) or a confounding factor (eg, coprescription with a drug already known to induce RP).

### Exploration of mechanistic hypotheses

All targets of drugs considered already known or probably or possibly associated with RP have been extracted from DrugBank. The most frequent pharmacological targets of these drugs are beta‐ and alpha‐adrenergic receptor inhibition, 5‐HT receptor inhibition (5‐HT_7_, 5‐HT_2C_, 5‐HT_2A_), and 5‐HT receptor agonism (5‐HT_1B_, 5‐HT_1D_, and 5‐HT_1A_). The targeted receptor associated with the higher number of ICSRs was sodium‐dependent noradrenaline transporter inhibitor (ie, amphetamine‐like drugs, nortriptyline, reboxetine, and milnacipran). Targets per class are displayed in Supplementary Figure 1.

### Network clustering

Among the 4,430 cases analyzed, 2,981 cases of RP were reported in association with other symptoms. After estimating event‐event disproportionate reporting, we found 34 adverse events disproportionately co‐reported with RP for beta blockers, 24 with antimigraine drugs, 23 with amphetamine‐like drugs, and 59 with TKIs. Details of all Preferred Terms disproportionally reported with RP with each class are available in Supplementary Table 3.

RP with beta blockers was notably associated with skin discoloration, peripheral coldness, hypoesthesia, neuralgia, and nipple pain. RP with amphetamine‐like drugs was associated with skin discoloration, cyanosis, peripheral coldness, chilblain, and pain in extremity. For antimigraine drugs, RP was associated with peripheral coldness, skin discoloration, poor peripheral circulation, condition aggravated, and feeling cold. Lastly, for TKIs, RP was associated with dry mouth, dry eye, skin discoloration, hypoesthesia, and neuropathy peripheral.

Clusters using PPMI matrix and Ising's model for the four pharmacological classes are available in Supplementary Figures 2 to 5. These clusters illustrate the differences in the pattern of associated symptoms reported along with RP according to drug classes. RP with beta blockers was notably associated with vasospasm and Prinzmetal angina. RP with antimigraine drugs was associated with conditions such as aggravated, systemic lupus erythematosus or extremity necrosis. RP with amphetamine‐like drugs were co‐reported with vasculitis and gangrene but also with adverse events related to autoimmune diseases (eg, vasculitis, systemic lupus erythematosus, scleroderma, or antinuclear antibody positive). Cases of RP reported with TKIs used in oncology were associated with terms more related to cardiovascular disorder (eg, peripheral arterial occlusive disease) or Sjögren disease (eg, dry eye or dry mouth) than a true RP.

## DISCUSSION

In this signal detection study using data from the WHO pharmacovigilance database, we identified 39 drugs with an SDR of RP that were not known to be associated with RP. These SDRs have been prioritized according to predefined criteria such as the robustness of the finding in sensitivity analyses, the characteristics of cases, and pharmacological plausibility of the association.

In particular, the seven antimigraine anti‐CGRP drugs display a robust signal in our study. This signal is pharmacologically plausible, CGRP deficiency having been identified in RP.^21^, ^22^, ^23^ The signal is robust in sensitivity analyses, and some cases show plausible time to onset and improvement after withdrawal, although anti‐CGRP drugs are often coprescribed with other antimigraine drugs known to be associated with RP, such as triptans. This signal is consistent with other pharmacovigilance disproportionality analyses and with a retrospective cohort study suggesting a deterioration of some patients with RP after initiating anti‐CGRP drugs.^5^, ^24^ However, given the increased baseline risk of RP in patients with migraine, regulators did not consider the evidence strong enough to validate the signal.^25^, ^26^ This illustrates the difficulty of validating RP signals given that this outcome is absent from claims databases and often not reported in clinical trials.

We also found robust and plausible signals with sympathomimetic drugs, such as modafinil, benfluorex, or solriamfetol, sharing pharmacological mechanisms with other amphetaminic‐like drugs and stimulants known to be associated with RP.^4^ Central stimulation of the dopaminergic and noradrenergic system is indeed responsible for the peripheral release of catecholamines leading to vasoconstriction. Plausible signals with reboxetine, an inhibitor of norepinephrine reuptake, and milnacipran, a selective serotonin and norepinephrine reuptake inhibitor, could be related shared pharmacological mechanisms.

Drugs with central alpha2A‐adrenergic receptor agonism such as clonidine or guanfacine also displayed strong and robust signals of RP. Clonidine‐induced RP is a well‐known adverse drug reaction that has been described for many years, although its frequency is still unknown.^4^ Relatedly, the increase of cold‐amplified α2c‐adrenoceptor–mediated vasoconstriction, via a pathway involving RhoA‐Rho kinase, is now considered one of the main mechanisms driving RP.^27^ This mechanism may also explain RP in patients exposed to cabergoline and bromocriptine, who also displayed affinity for α2c‐adrenoceptors.

Several TKIs also displayed significant SDRs of RP. Although the pharmacological mechanism is still unknown, TKIs such as nilotinib have been associated with vascular adverse events.^28^ In contrast to other TKIs, nilotinib was considered plausibly associated with RP, particularly because we found case reports describing the onset of PR during the first week of treatment.^29^

For many years, it has been uncertain whether oral contraceptives may induce RP. On the one hand, Jarrett described an improvement in symptoms after discontinuing treatment in three patients, associated with a decrease and normalization of plasma fibrinogen, which supports a causal role of these drugs.^30^ On the other hand, Bartelink and colleagues did not find any significant effect of a single dose of oral contraceptive (17β‐estradiol or progesterone) on finger skin circulation, either in healthy women or in women with a previous diagnosis of RP.^31^ Moreover, initiation of oral contraceptives in young women may also correspond to the natural occurrence of the disease that may be confounded by the role of the drug. We therefore need more robust studies to further explore this association, which could have significant burden in exposed women.

Network clustering with β‐adrenoceptor blockers support the hypothesis of a drug mediated vasoconstriction. Vasospasm of coronary arteries with β‐adrenoceptor blockers have already been described in the SmPC, and vasoconstriction is a pharmacological mechanism that could lead to RP.^4^ The other terms found in the network do not appear to be related to RP and seem to refer more to comorbidities associated with the indication for beta blockers. Network clustering of antimigraine drugs and amphetamine‐like drugs did not suggest any specific mechanism underlying RP. The network clustering of TKIs seems more related to underlying disease and oncology coprescription than a TKI‐specific effect.

Numerous drugs known to be associated with RP also displayed significant SDRs in our study. Yet, the global level of evidence of these “known drugs” is poor, mostly coming from case reports and small retrospective epidemiologic studies.^4^ Even β‐adrenoceptor blockers, one of the first class of drugs identified as associated with RP, are poorly characterized. Indeed, data about the prevalence of RP in exposed patients are scarce; the pharmacological mechanism is unclear, and differences according to their pharmacological action may probably exist. A network meta‐analysis of randomized controlled trials (RCTs) performed by our group found a large heterogeneity in the risk of peripheral vasoconstriction due ancillary properties of β‐adrenoceptor blockers (eg, intrinsic sympathomimetic activity, β1 selectivity, vasodilator activity).^32^ However, our study, like the network meta‐analysis of RCTs, was unable to highlight a significant safety signal for pindolol despite the presence of RP in its SmPC.

The lack of robust data even for well‐known drug classes associated with RP illustrates the difficulty in detecting and validating signals of RP. We were first surprised by the low number of cases of RP reported in the WHO pharmacovigilance database, with only 5,000 cases reported globally, in regard to the high prevalence of RP in the population ranging from 3 to 5%.^33^ In comparison, more than 10,000 cases of pulmonary arterial hypertension are reported, whereas the prevalence of the disease ranges from 20 to 50 cases per million adults. These numbers highlight the difficulty in achieving an objective aggravation of the disease in susceptible individuals given the variability of geographic conditions and complex factors driving the reporting of cases in pharmacovigilance systems such as the novelty of the drug, the severity of the adverse reaction, attitudes of physicians, or alerts in media. There is also a need to improve the coding of such symptoms in pharmacovigilance reporting systems and clinical trials, to avoid nonspecific terms such as cold extremities, poor peripheral circulation, or skin discoloration. In theory, it would be possible to quantify drug‐associated RP in clinical trials, given its prevalence in the population. However, given the coding heterogeneity mentioned above, a likely lack of reporting due to its low severity, and the low representation of women in clinical trials, very few clinical trials report data on RP. After a quick search in ClinicalTrials.gov, among the 65,996 trials reporting their results and using a very broad definition of RP (“Raynaud ulcer,” “Cold extremities,” “Peripheral coldness,” “Raynaud attack,” “Raynaud's syndrome,” “Raynaud's disease,” “Raynaud's,” “Raynaud's phenomenon,” “Raynaud phenomenon,” “Peripheral coldness,” “Raynaud,” “Cold exposure injury”), we only retrieved 94 different clinical trials reporting at least one case of RP. Moreover, although claims databases are one of the primary sources of evidence about drug safety, they are unable to capture RP given the large proportion of untreated patients and the minor proportion of hospitalized patients for this disease.

We therefore need to develop suited methods to identify drug‐associated RP. First, as health professionals, we need to focus more on patients’ experience and to enjoin them to report symptoms such as cold extremities, finger pain or tingling, or unusual aggravation of their disease to pharmacovigilance centers. Moreover, N‐of‐1 trials have already been conducted to assess efficacy of drugs in RP but could also be used to evaluate an aggravation of RP during drug exposure, as what was performed with statin and myalgia.^34^, ^35^ Lastly, prospective observational studies or using general practitioners’ electronic health records, such as the Clinical Practice Research Datalink, may be better suited to identify RP than claims databases.

We also encountered a few issues that we tried to address. First, given the risk of misclassification of RP using nonspecific terms such as cold extremities or poor peripheral circulation, we have chosen to restrict, in our main analysis, the definition of our outcome to the MeDRA Preferred Term “Raynaud's phenomenon.” We therefore could have missed some cases coded with such nonspecific terms. Moreover, we did not have access to narratives of cases, limiting our ability to assess their intrinsic validity and causality. Yet, we tried to leverage all available information about patient age, sex, comedications, and time to onset to assess the plausibility of drugs with significant SDRs. Our study also suffers from biases related to adverse event reporting systems and observational design such as selective reporting, selection bias, Weber effect, indication bias, confounding factors, sparse data bias, or masking effect.^36^ However, we tried to perform several sensitivity analyses to assess the potential impact of these biases on our results.

We can also stress certain limitations of cluster analysis using pharmacovigilance data. First, given non–drug‐related adverse events such as symptoms related to the underlying disease or adverse events due to coprescription are co‐reported, it is difficult to distinguish between drug‐related and these adverse effects in the clusters.^37^, ^38^ Methodologic developments allowing to identify and exclude these misattributed symptoms from pharmacovigilance databases may help improve signal detection and clustering analyses. Second, given the small number of cases reported for each of the classes studied, the clusters were unstable and highly dependent on the method used.

In conclusion, in this pharmacovigilance signal detection study, we found robust and significant SDRs for several drugs unknown to be associated with onset or aggravation of RP, such as CGRP inhibitors, some TKI, or solriamfetol. There is a need to perform purposed deigned studies to evaluate these signals and quantify the risk of RP associated with exposure to these drugs.

## AUTHOR CONTRIBUTIONS

All authors contributed to at least one of the following manuscript preparation roles: conceptualization AND/OR methodology, software, investigation, formal analysis, data curation, visualization, and validation AND drafting or reviewing/editing the final draft. As corresponding author, Dr Khouri confirms that all authors have provided the final approval of the version to be published and takes responsibility for the affirmations regarding article submission (eg, not under consideration by another journal), the integrity of the data presented, and the statements regarding compliance with institutional review board/Declaration of Helsinki requirements.

## Supporting information


**Disclosure Form**:

**Data S1** Supporting Information
