# Supplementary material for: Drug‐Induced Raynaud's Phenomenon and Underlying Mechanism: A Disproportionality Analysis From the World Health Organization Pharmacovigilance Database
Source: Arthritis Rheumatol. 2026 Feb 3;78(4):985–92. doi: 10.1002/art.43442 (PMC13054453; doi:10.1002/art.43442)
Supplement: Supplementary file 2 — Data S1 Supporting Information [file ART-78-985-s002.docx]

**Drugs induced Raynaud’s phenomenon and underlying mechanism: a disproportionality analysis from the WHO pharmacovigilance database**

Alex Hlavaty, Loubna Dari, Jean-Luc Cracowski, Matthieu Roustit, Charles Khouri

**Supplementary Material**

Table of content

[Supplementary Table 1. The READUS-PV checklist 2](#_Toc210745280)

[Supplementary Table 2. Plausibility assessment by experts in pharmacovigilance, vascular disease and pharmacology 4](#_Toc210745281)

[Supplementary Table 3. Cases descriptions for each drug with a SDR in main analysis 5](#_Toc210745282)

[Supplementary Table 4. Preferred terms associated with an event-event disproportionate reporting using narrow definition of Raynaud's phenomenon among each drug class 48](#_Toc210745283)

[Supplementary Figure 1. Sankey diagram representing target of each drug per class using DrugBank database 50](#_Toc210745284)

[Supplementary Figure 2. Network clustering using PPMI matrix and Ising model for PTs associated with beta adrenoreceptor blockers 62](#_Toc210745285)

# Supplementary Table 1. The READUS-PV checklist

| **Section and topic** | **Item #** | **Checklist item** | **Location where item is reported** |
| --- | --- | --- | --- |
| **Title** |  |  |  |
|  | *1a* | *If disproportionality analyses are a prominent component of the published study, the study should be identified as a “disproportionality analysis”. The type of data and name of the database(s) should be specified.* | *Title* |
|  | *1b* | *Report the name of adverse event(s) and/or drug(s) under study, when applicable.* | *Title* |
| **Introduction** |  |  |  |
| Background | *2a* | *Describe the drug(s) and its utilization, the nature of the adverse event(s) under study and its frequency, and the existing knowledge on the drug-event combination.* | *Page 5* |
|  | *2b* | *Specify the rationale for performing the analysis, e.g., as part of routine pharmacovigilance, to investigate an overall safety profile, or to assess a pre-specified hypothesis.* | *Page 5* |
|  | *2c* | *Explain why ICSR databases and disproportionality analysis are suitable to fill the knowledge gap.* | *Page 5* |
| Objectives | *3* | *State specific objectives, identifying the adverse event(s), the drug(s), and the reference group, including any pre-specified hypothesis, if applicable.* | *Page 5* |
| **Methods** |  |  |  |
| Study design | *4a* | *Identify the study (i.e., “disproportionality analysis”) and the type of data used (e.g., “individual case safety reports”).* | *Page 5-8* |
|  | *4b* | *Provide an outline of the entire study design, including primary and sensitivity analyses performed, and other designs such as case-by-case analysis or literature review.* | *Page 5-8* |
| Data description, access, and pre-processing | *5a* | *Specify the name of the database(s), the database(s) custodian, and the coverage. Specify the type/number of drugs included within the database and the thesaurus, taxonomies, or ontologies used for coding drugs and events.* | *Page 5-8* |
|  | *5b* | *Specify the extraction dates and describe and justify all choices used for data pre-processing, including any data transformation or exclusion, if appropriate.* | *Page 5-8* |
| Variables definition | *6a* | *Describe the study population, including any restriction.* | *Page 5-8* |
|  | *6b* | *Describe the nature and the meaning of key variables assessed in the work.* | *Page 5-8* |
|  | *6c* | *Specify and justify any grouping of drugs or events. For drugs, specify and justify whether active ingredients/trade names/salts were considered and/or the selected role.* | *Page 5-8* |
|  | *6d* | *Describe any additional data source used, the type of data, and how they interact with ICSRs.* | *Page 5-8* |
| Statistical methods | *7a* | *Present any descriptive analysis performed, specifying variables investigated, statistical tests, and significance thresholds.* | *Page 5-8* |
|  | *7b* | *Describe the measure(s) selected for the disproportionality analysis including any threshold used to identify signals of disproportionate reporting. Explain the reason for this choice if applicable.* | *Page 5-8* |
|  | *7c* | *Clearly describe any sensitivity analysis and any tool to control confounding, including any restriction, subgroup, stratification, adjustment, or interaction.* | *Page 5-8* |
|  | *7d* | *Specify the variables and methods used for the case-by-case analysis, including any algorithm or criteria used to assess causality, if performed.* | *Page 5-8* |
|  | *7e* | *Specify any statistical methods used for other data sources.* | *Page 5-8* |
| **Results** |  |  |  |
| Participants | *8a* | *Specify the number of individual case safety reports included at each stage, including reasons for exclusion.* | *Page 9* |
|  | *8b* | *Provide key demographic and clinical characteristics of cases, if possible comparing cases with any appropriate reference group.* | *Supplementary materials Table 1* |
| Disproportionality analysis | *9* | *Present all results including confidence intervals. Present also results of sensitivity analyses, if performed.* | *Supplementary materials Table 1 - 2* |
| Case-by-case analysis | *10* | *Present the case-by-case analysis of key variables. Present the causality assessment, if applicable.* | *Page 9-10* |
| **Discussion** |  |  |  |
| Key results | *11* | *Discuss key results with reference to study objectives and contextualize them within the current literature and other consulted sources. Clearly discriminate between expected reactions and emerging safety signals.* | *Page 13-14* |
| External validity | *12a* | *Discuss the external validity of the results to the general population.* | *Page 13-14* |
|  | *12b* | *Discuss the potential relevance of results in clinical practice* | *Page 13-16* |
|  | *12c* | *Propose further study designs if applicable* | *Page 13-16* |
| Limitations | *13* | *Present general limitations, making clear that disproportionality analysis alone cannot prove causation or measure incidence, and specific limitations, including confounding and reporting bias and efforts to mitigate them.* | *Page 17-18* |
| **Declarations** |  |  |  |
|  | *14a* | *Provide the source of funding/sponsorship and the role of the funders/sponsors for the present study and for any original study on which the present article is based.* | *Page 19-20* |
|  | *14b* | *Clearly identify potential commercial and intellectual conflicts of interest (e.g., link to any drug/event investigated, whether financial, legal action, or software used).* | *Page 19-20* |
|  | *14c* | *Declare any institutional approval needed or granted in the investigation.* | *Page 19-20* |
|  | *14d* | *Include a statement on data availability, code availability (including the version of the statistical software used), and protocol registration.* | *Page 19-20* |

Table 1 - READUS-PV Checklist

| **Probable** | **Possible** | **Unlikely** | **Already known** |
| --- | --- | --- | --- |
| Milnacipran | Finasteride | Methotrexate | Sumatriptan |
| Modafinil | Topiramate | Risedronic acid | Gemcitabine |
| Reboxetine | Vinblastine | Alendronic acid | Amfetamine;Dexamfetamine |
| Fremanezumab | Ethinylestradiol;Levonorgestrel | Mycophenolic acid | Nebivolol |
| Bromocriptine | Drospirenone;Ethinylestradiol | Raloxifene | Zolmitriptan |
| Guanfacine | Ethinylestradiol;Etonogestrel | Anastrozole | Rizatriptan |
| Nilotinib | Dienogest;Ethinylestradiol | Alendronic acid;Colecalciferol | Eletriptan |
| Benfluorex | Avapritinib | Rofecoxib | Peginterferon alfa-2b |
| Ubrogepant | Immunoglobulin g human | Rituximab | Peginterferon alfa-2a |
| Rimegepant | Aripiprazole | Leflunomide | Dexmethylphenidate |
| Cabergoline | Minocycline | Zoledronic acid | Atomoxetine |
| Galcanezumab | Amitriptyline | Infliximab | Dihydroergotamine |
| Erenumab | Amantadine | Paricalcitol | Clonidine |
| Eptinezumab | Teriflunomide | Pemetrexed | Dexamfetamine |
| Solriamfetol | Pergolide | Etanercept | Bleomycin |
| Atogepant | Amifampridine | Gamma-hydroxybutyrate;Oxybate sodium | Ergotamine |
|  | Semaglutide | Imatinib | Propranolol |
|  | Nortriptyline | Covid-19 vaccine | Timolol |
|  | Pembrolizumab | Agalsidase alfa | Amfetamine |
|  | Selpercatinib | Tolvaptan | Metoprolol |
|  | Ponatinib | Tocilizumab | Sotalol |
|  | Lorlatinib | Prednisone | Acebutolol |
|  | Omalizumab | Corticotropin | Labetalol |
|  | Pramipexole | Ocrelizumab | Atenolol |
|  |  | Isotretinoin | Nadolol |
|  |  | Golimumab | Carteolol |
|  |  | Belimumab | Interferon beta |
|  |  | Hydroxychloroquine | Interferon alfa-2b |
|  |  | Asfotase alfa | Interferon beta-1b |
|  |  | Pamidronic acid | Interferon beta-1a |
|  |  | Ribavirin | Lisdexamfetamine |
|  |  | Tofacitinib | Interferon alfa-2a |
|  |  | Simvastatin | Armodafinil |
|  |  | Certolizumab pegol | Betaxolol |
|  |  | Voclosporin | Bisoprolol |
|  |  | Melatonin | Celiprolol |
|  |  | Collagenase clostridium histolyticum | Carvedilol |
|  |  | Cinacalcet | Dexmethylphenidate;Serdexmethylphenidate |
|  |  | Adalimumab | Peginterferon beta-1a |
|  |  | Lithium | Methylphenidate |
|  |  | Ofatumumab |  |
|  |  | Agalsidase beta |  |
|  |  | Sulfasalazine |  |
|  |  | Oxybate sodium |  |

# Supplementary Table 2. Plausibility assessment by experts in pharmacovigilance, vascular disease and pharmacology

|  | **Lisdexamfetamine** | **Dexamfetamine** | **Methylphenidate** |
| --- | --- | --- | --- |
| **Number of cases** | 188 | 35 | 217 |
| **IC025_RP_main** | 5.47 | 4.46 | 4.48 |
| **IC025_RP_comparator** | 1.54 | 1.1 | 0.43 |
| **IC025_RP_healthcare** | 5.23 | 2.8 | 4.89 |
| **IC025_RP_malefemale** | 5.29 | 3.56 | 4.57 |
| **IC025_RP-Broad_main** | 4.59 | 4.66 | 3.78 |
| **Sex (Female / Male / Unknown)** | 110 / 58 / 20 | 23 / 11 / 1 | 106 / 109 / 2 |
| **Mean age (SD) in years** | 26.45 (14.52) years | 29.95 (11.14) years | 18.65 (10.19) years |
| **Mean number of suspect/interacting drugs (SD)** | 2.31 (2.15) | 3.62 (5.45) | 1.87 (1.66) |
| **Withdrawn** | 59/188 (31.38 %) | 5/35 (14.29 %) | 67/217 (30.88 %) |
| **Outcome after drug withdrawal** | Recovered: 22/59 (37.29 %) Recovering: 8/59 (13.56 %) Recovered with sequelae: 2/59 (3.39 %) Not recovered: 6/59 (10.17 %) Evolution Unknown: 13/59 (22.03 %) | Recovering: 2/5 (40.0 %) Evolution Unknown: 3/5 (60.0 %) | Recovered: 29/67 (43.28 %) Recovering: 7/67 (10.45 %) Recovered with sequelae: 3/67 (4.48 %) Not recovered: 10/67 (14.93 %) Evolution Unknown: 10/67 (14.93 %) |
| **Country of primary source** | United States of America: 90/188 (47.9 %) Sweden: 24/188 (12.8 %) Netherlands: 18/188 (9.6 %) United Kingdom of Great Britain and Northern Ireland: 16/188 (8.5 %) Germany: 15/188 (8.0 %) | Netherlands: 27/35 (77.1 %) United States of America: 3/35 (8.6 %) United Kingdom of Great Britain and Northern Ireland: 2/35 (5.7 %) France: 2/35 (5.7 %) Sweden: 1/35 (2.9 %) | United States of America: 36/217 (16.6 %) Netherlands: 34/217 (15.7 %) Germany: 29/217 (13.4 %) United Kingdom of Great Britain and Northern Ireland: 28/217 (12.9 %) Sweden: 19/217 (8.8 %) |
| **Co-reported drugs** | Methylphenidate hydrochloride: 11/ 188 (5.9 %) Methylphenidate: 11/ 188 (5.9 %) Amfetamine aspartate;Amfetamine sulfate;Dexamfetamine saccharate;Dexamfetamine sulfate: 9/ 188 (4.8 %) Amfetamine aspartate monohydrate;Amfetamine sulfate;Dexamfetamine saccharate;Dexamfetamine sulfate: 9/ 188 (4.8 %) Guanfacine hydrochloride: 7/ 188 (3.7 %) | Methylphenidate: 3/ 35 (8.6 %) Methylphenidate hydrochloride: 3/ 35 (8.6 %) Salbutamol sulfate: 3/ 35 (8.6 %) Salbutamol: 3/ 35 (8.6 %) Lisdexamfetamine mesilate: 3/ 35 (8.6 %) | Melatonin: 10/ 217 (4.6 %) Lisdexamfetamine mesilate: 8/ 217 (3.7 %) Guanfacine hydrochloride: 6/ 217 (2.8 %) Aripiprazole: 6/ 217 (2.8 %) Modafinil: 5/ 217 (2.3 %) |
| **Co-reported reactions** | Peripheral coldness: 11/188 (5.9 %) Decreased appetite: 9/188 (4.8 %) Skin discolouration: 7/188 (3.7 %) Peripheral swelling: 7/188 (3.7 %) Anxiety: 7/188 (3.7 %) | Therapeutic response unexpected: 7/35 (20.0 %) Palpitations: 5/35 (14.3 %) Bruxism: 3/35 (8.6 %) Anxiety: 3/35 (8.6 %) Headache: 2/35 (5.7 %) | Drug ineffective: 10/217 (4.6 %) Cyanosis: 9/217 (4.1 %) Skin discolouration: 8/217 (3.7 %) Peripheral coldness: 7/217 (3.2 %) Paraesthesia: 7/217 (3.2 %) |
| **Reporter qualification** | Healthcare professional : 89/188 (47.34 %) Non healthcare professional : 99/188 (52.66 %) | Healthcare professional : 9/35 (25.71 %) Non healthcare professional : 26/35 (74.29 %) | Healthcare professional : 161/217 (74.19 %) Non healthcare professional : 56/217 (25.81 %) |
| **Indications** | ADHD : 56/188 (29.8 %) Attention deficit/hyperactivity disorder : 41/188 (21.8 %) Unknown : 31/188 (16.5 %) Product used for unknown indication : 30/188 (16.0 %) Drug use for unknown indication : 17/188 (9.0 %) | ADHD : 19/35 (54.3 %) ADD : 5/35 (14.3 %) Narcolepsy : 3/35 (8.6 %) ADHD, combined type : 2/35 (5.7 %) Product used for unknown indication : 2/35 (5.7 %) | Unknown : 53/217 (24.4 %) ADHD : 49/217 (22.6 %) Attention deficit/hyperactivity disorder : 38/217 (17.5 %) ADD : 15/217 (6.9 %) Attention deficit-hyperactivity disorder : 14/217 (6.5 %) |
| **Mean Time to Onset (std)** | 166 days (277 days) | 273 days (447 days) | 401 days (571 days) |

# Supplementary Table *3*. Cases descriptions for each drug with a SDR in main analysis

|  | **Atomoxetine** | **Cabergoline** | **Oxybate sodium** |
| --- | --- | --- | --- |
| **Number of cases** | 76 | 23 | 141 |
| **IC025_RP_main** | 3.89 | 3.82 | 3.78 |
| **IC025_RP_comparator** | -0.06 | 3.61 | 3.01 |
| **IC025_RP_healthcare** | 4.44 | 3.48 | 3.45 |
| **IC025_RP_malefemale** | 3.45 | 3.6 | 3.19 |
| **IC025_RP-Broad_main** | 2.94 | 3.64 | 2.61 |
| **Sex (Female / Male / Unknown)** | 33 / 41 / 2 | 17 / 5 / 1 | 128 / 11 / 2 |
| **Mean age (SD) in years** | 19.77 (13.56) years | 46.74 (17.54) years | 42.68 (14.0) years |
| **Mean number of suspect/interacting drugs (SD)** | 1.92 (1.38) | 2.17 (1.6) | 11.78 (9.76) |
| **Withdrawn** | 26/76 (34.21 %) | 11/23 (47.83 %) | 53/141 (37.59 %) |
| **Outcome after drug withdrawal** | Recovered: 9/26 (34.62 %) Recovering: 4/26 (15.38 %) Not recovered: 3/26 (11.54 %) Evolution Unknown: 6/26 (23.08 %) | Recovered: 4/11 (36.36 %) Recovering: 3/11 (27.27 %) Not recovered: 3/11 (27.27 %) | Recovering: 1/53 (1.89 %) Not recovered: 6/53 (11.32 %) Evolution Unknown: 12/53 (22.64 %) |
| **Country of primary source** | United States of America: 20/76 (26.3 %) Germany: 11/76 (14.5 %) Netherlands: 8/76 (10.5 %) United Kingdom of Great Britain and Northern Ireland: 6/76 (7.9 %) Denmark: 5/76 (6.6 %) | United Kingdom of Great Britain and Northern Ireland: 5/23 (21.7 %) France: 5/23 (21.7 %) Spain: 4/23 (17.4 %) Netherlands: 3/23 (13.0 %) United States of America: 2/23 (8.7 %) | United States of America: 139/141 (98.6 %) Canada: 1/141 (0.7 %) France: 1/141 (0.7 %) |
| **Co-reported drugs** | Methylphenidate hydrochloride: 10/ 76 (13.2 %) Methylphenidate: 10/ 76 (13.2 %) Omeprazole: 2/ 76 (2.6 %) Propranolol: 2/ 76 (2.6 %) Risperidone: 2/ 76 (2.6 %) | Levothyroxine sodium: 3/ 23 (13.0 %) Levothyroxine: 3/ 23 (13.0 %) Selegiline: 2/ 23 (8.7 %) Diazepam: 1/ 23 (4.3 %) Hydrocortisone: 1/ 23 (4.3 %) | Amfetamine aspartate;Amfetamine sulfate;Dexamfetamine saccharate;Dexamfetamine sulfate: 52/ 141 (36.9 %) Armodafinil: 33/ 141 (23.4 %) Colecalciferol: 24/ 141 (17.0 %) Omeprazole: 23/ 141 (16.3 %) Omeprazole magnesium: 23/ 141 (16.3 %) |
| **Co-reported reactions** | Peripheral coldness: 5/76 (6.6 %) Urinary retention: 3/76 (3.9 %) Fatigue: 3/76 (3.9 %) Cyanosis: 3/76 (3.9 %) Skin discolouration: 2/76 (2.6 %) | Therapeutic product effect incomplete: 1/23 (4.3 %) Vasospasm: 1/23 (4.3 %) Nail dystrophy: 1/23 (4.3 %) Hypertrichosis: 1/23 (4.3 %) Flushing: 1/23 (4.3 %) | Anxiety: 20/141 (14.2 %) Condition aggravated: 16/141 (11.3 %) Blood pressure increased: 12/141 (8.5 %) Depression: 12/141 (8.5 %) Sleep apnoea syndrome: 11/141 (7.8 %) |
| **Reporter qualification** | Healthcare professional : 62/76 (81.58 %) Non healthcare professional : 14/76 (18.42 %) | Healthcare professional : 17/23 (73.91 %) Non healthcare professional : 6/23 (26.09 %) | Healthcare professional : 47/141 (33.33 %) Non healthcare professional : 94/141 (66.67 %) |
| **Indications** | Unknown : 22/76 (28.9 %) ADHD : 21/76 (27.6 %) Attention deficit/hyperactivity disorder : 15/76 (19.7 %) Drug use for unknown indication : 7/76 (9.2 %) Attention deficit-hyperactivity disorder : 4/76 (5.3 %) | Prolactinoma : 6/23 (26.1 %) Unknown : 6/23 (26.1 %) Parkinson's disease : 3/23 (13.0 %) Macroprolactinaemia : 2/23 (8.7 %) Hyperprolactinaemia : 2/23 (8.7 %) | Narcolepsy : 122/141 (86.5 %) Unknown : 100/141 (70.9 %) Cataplexy : 75/141 (53.2 %) Somnolence : 30/141 (21.3 %) Hypersomnia : 9/141 (6.4 %) |
| **Mean Time to Onset (std)** | 275 days (438 days) | 669 days (1293 days) | nan |

|  | **Fremanezumab** | **Bleomycin** | **Galcanezumab** |
| --- | --- | --- | --- |
| **Number of cases** | 42 | 35 | 62 |
| **IC025_RP_main** | 3.66 | 3.55 | 3.55 |
| **IC025_RP_comparator** | 1.46 | 3.04 | 1.22 |
| **IC025_RP_healthcare** | 3.5 | 3.29 | 4.09 |
| **IC025_RP_malefemale** | 3.63 | 3.58 | 3.75 |
| **IC025_RP-Broad_main** | 2.84 | 3.55 | 2.67 |
| **Sex (Female / Male / Unknown)** | 39 / 3 / 0 | 10 / 24 / 1 | 55 / 3 / 4 |
| **Mean age (SD) in years** | 45.36 (11.01) years | 37.03 (12.94) years | 43.42 (13.17) years |
| **Mean number of suspect/interacting drugs (SD)** | 3.74 (3.49) | 3.86 (2.61) | 3.71 (2.78) |
| **Withdrawn** | 9/42 (21.43 %) | 8/35 (22.86 %) | 18/62 (29.03 %) |
| **Outcome after drug withdrawal** | Recovered: 3/9 (33.33 %) Recovering: 1/9 (11.11 %) Not recovered: 3/9 (33.33 %) Evolution Unknown: 2/9 (22.22 %) | Recovered: 2/8 (25.0 %) Not recovered: 6/8 (75.0 %) | Recovered: 5/18 (27.78 %) Recovering: 1/18 (5.56 %) Not recovered: 3/18 (16.67 %) Evolution Unknown: 7/18 (38.89 %) |
| **Country of primary source** | United Kingdom of Great Britain and Northern Ireland: 9/42 (21.4 %) United States of America: 6/42 (14.3 %) Netherlands: 5/42 (11.9 %) Norway: 4/42 (9.5 %) Germany: 3/42 (7.1 %) | France: 20/35 (57.1 %) Germany: 3/35 (8.6 %) United States of America: 2/35 (5.7 %) United Kingdom of Great Britain and Northern Ireland: 2/35 (5.7 %) Switzerland: 2/35 (5.7 %) | United States of America: 37/62 (59.7 %) Spain: 7/62 (11.3 %) Italy: 7/62 (11.3 %) France: 3/62 (4.8 %) United Kingdom of Great Britain and Northern Ireland: 2/62 (3.2 %) |
| **Co-reported drugs** | Candesartan: 4/ 42 (9.5 %) Sumatriptan: 4/ 42 (9.5 %) Amitriptyline: 4/ 42 (9.5 %) Amitriptyline hydrochloride: 4/ 42 (9.5 %) Salbutamol: 3/ 42 (7.1 %) | Doxorubicin hydrochloride: 13/ 35 (37.1 %) Doxorubicin: 13/ 35 (37.1 %) Cisplatin: 12/ 35 (34.3 %) Etoposide: 12/ 35 (34.3 %) Dacarbazine: 10/ 35 (28.6 %) | Sumatriptan succinate: 6/ 62 (9.7 %) Sumatriptan: 6/ 62 (9.7 %) Rizatriptan: 5/ 62 (8.1 %) Rizatriptan benzoate: 5/ 62 (8.1 %) Propranolol hydrochloride: 4/ 62 (6.5 %) |
| **Co-reported reactions** | Arthralgia: 4/42 (9.5 %) Nausea: 4/42 (9.5 %) Alopecia: 4/42 (9.5 %) Weight increased: 4/42 (9.5 %) Constipation: 3/42 (7.1 %) | Osteonecrosis: 3/35 (8.6 %) Pancytopenia: 2/35 (5.7 %) Skin necrosis: 2/35 (5.7 %) Candida infection: 1/35 (2.9 %) Gait disturbance: 1/35 (2.9 %) | Arthralgia: 6/62 (9.7 %) Chills: 4/62 (6.5 %) Alopecia: 4/62 (6.5 %) Constipation: 3/62 (4.8 %) Weight increased: 3/62 (4.8 %) |
| **Reporter qualification** | Healthcare professional : 22/42 (52.38 %) Non healthcare professional : 20/42 (47.62 %) | Healthcare professional : 28/35 (80.0 %) Non healthcare professional : 7/35 (20.0 %) | Healthcare professional : 35/62 (56.45 %) Non healthcare professional : 27/62 (43.55 %) |
| **Indications** | Migraine : 19/42 (45.2 %) Unknown : 10/42 (23.8 %) Chronic migraine : 8/42 (19.0 %) Migraine prophylaxis : 4/42 (9.5 %) Adverse drug reaction NOS : 1/42 (2.4 %) | Unknown : 12/35 (34.3 %) Hodgkin's lymphoma : 4/35 (11.4 %) Testicular cancer : 3/35 (8.6 %) Hodgkin's disease : 3/35 (8.6 %) Drug use for unknown indication : 2/35 (5.7 %) | Migraine : 31/62 (50.0 %) Product used for unknown indication : 13/62 (21.0 %) Chronic migraine : 9/62 (14.5 %) Drug use for unknown indication : 4/62 (6.5 %) Unknown : 4/62 (6.5 %) |
| **Mean Time to Onset (std)** | 72 days (134 days) | 177 days (178 days) | 88 days (146 days) |

|  | **Erenumab** | **Bisoprolol** | **Nebivolol** |
| --- | --- | --- | --- |
| **Number of cases** | 103 | 59 | 26 |
| **IC025_RP_main** | 3.51 | 3.61 | 3.53 |
| **IC025_RP_comparator** | 1.11 | 0.27 | 0.51 |
| **IC025_RP_healthcare** | 3.7 | 3.08 | 3.28 |
| **IC025_RP_malefemale** | 3.08 | 3.5 | 3.3 |
| **IC025_RP-Broad_main** | 2.37 | 4.31 | 3.86 |
| **Sex (Female / Male / Unknown)** | 88 / 5 / 10 | 32 / 25 / 2 | 15 / 11 / 0 |
| **Mean age (SD) in years** | 45.51 (13.14) years | 63.4 (16.07) years | 55.06 (18.49) years |
| **Mean number of suspect/interacting drugs (SD)** | 3.95 (3.35) | 4.0 (2.82) | 4.6 (4.1) |
| **Withdrawn** | 28/103 (27.18 %) | 19/59 (32.2 %) | 13/26 (50.0 %) |
| **Outcome after drug withdrawal** | Recovered: 9/28 (32.14 %) Recovering: 1/28 (3.57 %) Not recovered: 7/28 (25.0 %) Evolution Unknown: 9/28 (32.14 %) | Recovered: 7/19 (36.84 %) Recovering: 4/19 (21.05 %) Not recovered: 2/19 (10.53 %) Evolution Unknown: 5/19 (26.32 %) | Recovered: 5/13 (38.46 %) Recovering: 2/13 (15.38 %) Not recovered: 2/13 (15.38 %) Evolution Unknown: 4/13 (30.77 %) |
| **Country of primary source** | United States of America: 47/103 (45.6 %) Germany: 16/103 (15.5 %) Netherlands: 6/103 (5.8 %) United Kingdom of Great Britain and Northern Ireland: 5/103 (4.9 %) Norway: 4/103 (3.9 %) | France: 22/59 (37.3 %) Germany: 7/59 (11.9 %) United Kingdom of Great Britain and Northern Ireland: 7/59 (11.9 %) Spain: 6/59 (10.2 %) Portugal: 3/59 (5.1 %) | France: 11/26 (42.3 %) United States of America: 4/26 (15.4 %) Belgium: 3/26 (11.5 %) Italy: 2/26 (7.7 %) United Kingdom of Great Britain and Northern Ireland: 2/26 (7.7 %) |
| **Co-reported drugs** | Rizatriptan benzoate: 11/ 103 (10.7 %) Rizatriptan: 11/ 103 (10.7 %) Sumatriptan: 8/ 103 (7.8 %) Propranolol: 7/ 103 (6.8 %) Propranolol hydrochloride: 7/ 103 (6.8 %) | Acetylsalicylic acid: 6/ 59 (10.2 %) Acetylsalicylate lysine: 6/ 59 (10.2 %) Atorvastatin: 6/ 59 (10.2 %) Atorvastatin calcium: 6/ 59 (10.2 %) Lansoprazole: 4/ 59 (6.8 %) | Acetylsalicylic acid: 4/ 26 (15.4 %) Acetylsalicylate lysine: 4/ 26 (15.4 %) Lisinopril: 3/ 26 (11.5 %) Esomeprazole: 3/ 26 (11.5 %) Furosemide: 3/ 26 (11.5 %) |
| **Co-reported reactions** | Constipation: 9/103 (8.7 %) Paraesthesia: 6/103 (5.8 %) Peripheral coldness: 6/103 (5.8 %) Condition aggravated: 6/103 (5.8 %) Hypoaesthesia: 5/103 (4.9 %) | Product use in unapproved indication: 4/59 (6.8 %) Bradycardia: 3/59 (5.1 %) Dizziness: 3/59 (5.1 %) Dry eye: 2/59 (3.4 %) Restlessness: 2/59 (3.4 %) | Chest pain: 2/26 (7.7 %) Nausea: 1/26 (3.8 %) Diarrhoea: 1/26 (3.8 %) Hypoaesthesia: 1/26 (3.8 %) Paraesthesia: 1/26 (3.8 %) |
| **Reporter qualification** | Healthcare professional : 69/103 (66.99 %) Non healthcare professional : 34/103 (33.01 %) | Healthcare professional : 37/59 (62.71 %) Non healthcare professional : 22/59 (37.29 %) | Healthcare professional : 19/26 (73.08 %) Non healthcare professional : 7/26 (26.92 %) |
| **Indications** | Migraine : 50/103 (48.5 %) Migraine prophylaxis : 15/103 (14.6 %) Product used for unknown indication : 14/103 (13.6 %) Chronic migraine : 10/103 (9.7 %) Migraine with aura : 6/103 (5.8 %) | Unknown : 13/59 (22.0 %) Drug use for unknown indication : 7/59 (11.9 %) Product used for unknown indication : 4/59 (6.8 %) Hypertension : 3/59 (5.1 %) Tachycardia : 3/59 (5.1 %) | Unknown : 9/26 (34.6 %) Hypertension : 5/26 (19.2 %) Hypertension arterial : 4/26 (15.4 %) Drug use for unknown indication : 2/26 (7.7 %) Cardiac arrhythmia : 1/26 (3.8 %) |
| **Mean Time to Onset (std)** | 204 days (305 days) | 374 days (828 days) | 65 days (101 days) |

|  | **Reboxetine** | **Labetalol** | **Guanfacine** |
| --- | --- | --- | --- |
| **Number of cases** | 14 | 19 | 18 |
| **IC025_RP_main** | 3.29 | 3.23 | 3.22 |
| **IC025_RP_comparator** | 3.16 | 0.34 | -0.12 |
| **IC025_RP_healthcare** | 2.22 | 2.84 | 3.27 |
| **IC025_RP_malefemale** | 3.19 | 2.97 | 2.7 |
| **IC025_RP-Broad_main** | 3.39 | 3.59 | 3.0 |
| **Sex (Female / Male / Unknown)** | 9 / 5 / 0 | 17 / 1 / 1 | 7 / 9 / 2 |
| **Mean age (SD) in years** | 50.08 (14.9) years | 35.47 (14.02) years | 14.83 (10.62) years |
| **Mean number of suspect/interacting drugs (SD)** | 2.1 (1.1) | 2.7 (2.16) | 1.79 (0.97) |
| **Withdrawn** | 2/14 (14.29 %) | 5/19 (26.32 %) | 5/18 (27.78 %) |
| **Outcome after drug withdrawal** | Recovered: 1/2 (50.0 %) Evolution Unknown: 1/2 (50.0 %) | Recovered: 2/5 (40.0 %) Recovering: 2/5 (40.0 %) Evolution Unknown: 1/5 (20.0 %) | Recovered: 3/5 (60.0 %) Evolution Unknown: 1/5 (20.0 %) |
| **Country of primary source** | United Kingdom of Great Britain and Northern Ireland: 8/14 (57.1 %) Germany: 2/14 (14.3 %) Australia: 2/14 (14.3 %) Sweden: 1/14 (7.1 %) Switzerland: 1/14 (7.1 %) | United Kingdom of Great Britain and Northern Ireland: 11/19 (57.9 %) Netherlands: 2/19 (10.5 %) Norway: 1/19 (5.3 %) Sweden: 1/19 (5.3 %) Iceland: 1/19 (5.3 %) | Germany: 6/18 (33.3 %) United States of America: 5/18 (27.8 %) United Kingdom of Great Britain and Northern Ireland: 2/18 (11.1 %) Sweden: 2/18 (11.1 %) Belgium: 1/18 (5.6 %) |
| **Co-reported drugs** | Zopiclone: 2/ 14 (14.3 %) Citalopram hydrobromide: 2/ 14 (14.3 %) Citalopram: 2/ 14 (14.3 %) Escitalopram oxalate: 1/ 14 (7.1 %) Sulfasalazine: 1/ 14 (7.1 %) | Folic acid: 3/ 19 (15.8 %) Acetylsalicylic acid: 2/ 19 (10.5 %) Paracetamol: 2/ 19 (10.5 %) Omeprazole: 2/ 19 (10.5 %) Vitamins nos: 2/ 19 (10.5 %) | Lisdexamfetamine mesilate: 7/ 18 (38.9 %) Methylphenidate: 7/ 18 (38.9 %) Methylphenidate hydrochloride: 7/ 18 (38.9 %) Amfetamine aspartate;Amfetamine sulfate;Dexamfetamine saccharate;Dexamfetamine sulfate: 2/ 18 (11.1 %) Fluoxetine hydrochloride: 1/ 18 (5.6 %) |
| **Co-reported reactions** | Hyperhidrosis: 2/14 (14.3 %) Adrenal gland cancer: 1/14 (7.1 %) Blood pressure fluctuation: 1/14 (7.1 %) Hypertension: 1/14 (7.1 %) Colitis ulcerative: 1/14 (7.1 %) | Maternal exposure during pregnancy: 6/19 (31.6 %) Nipple pain: 5/19 (26.3 %) Breast pain: 3/19 (15.8 %) Hypoaesthesia: 2/19 (10.5 %) Skin discolouration: 2/19 (10.5 %) | Skin discolouration: 3/18 (16.7 %) Drug ineffective: 2/18 (11.1 %) Decreased appetite: 1/18 (5.6 %) Condition aggravated: 1/18 (5.6 %) Hypotension: 1/18 (5.6 %) |
| **Reporter qualification** | Healthcare professional : 7/14 (50.0 %) Non healthcare professional : 7/14 (50.0 %) | Healthcare professional : 12/19 (63.16 %) Non healthcare professional : 7/19 (36.84 %) | Healthcare professional : 16/18 (88.89 %) Non healthcare professional : 2/18 (11.11 %) |
| **Indications** | Unknown : 8/14 (57.1 %) Depression : 5/14 (35.7 %) Depressed mood : 1/14 (7.1 %) MODERATE DEPRESSIVE EPISODE : 1/14 (7.1 %) | Pregnancy induced hypertension : 6/19 (31.6 %) Hypertension : 4/19 (21.1 %) Gestational hypertension : 3/19 (15.8 %) Unknown : 1/19 (5.3 %) Adverse drug reaction NOS : 1/19 (5.3 %) | ADHD : 6/18 (33.3 %) Drug use for unknown indication : 4/18 (22.2 %) Product used for unknown indication : 3/18 (16.7 %) Attention deficit/hyperactivity disorder : 2/18 (11.1 %) Tic : 1/18 (5.6 %) |
| **Mean Time to Onset (std)** | 277 days (644 days) | 117 days (295 days) | 158 days (179 days) |

|  | **Interferon alfa-2a** | **Gamma-hydroxybutyrate;Oxybate sodium** | **Propranolol** |
| --- | --- | --- | --- |
| **Number of cases** | 15 | 17 | 54 |
| **IC025_RP_main** | 3.18 | 3.08 | 3.2 |
| **IC025_RP_comparator** | 2.48 | 1.68 | 2.06 |
| **IC025_RP_healthcare** | 2.73 | 1.9 | 2.85 |
| **IC025_RP_malefemale** | 3.27 | 2.61 | 3.0 |
| **IC025_RP-Broad_main** | 3.6 | 2.39 | 4.73 |
| **Sex (Female / Male / Unknown)** | 6 / 9 / 0 | 14 / 3 / 0 | 44 / 9 / 1 |
| **Mean age (SD) in years** | 45.43 (11.01) years | 38.3 (17.97) years | 44.74 (22.99) years |
| **Mean number of suspect/interacting drugs (SD)** | 4.8 (1.48) | 9.25 (6.7) | 3.33 (2.92) |
| **Withdrawn** | 1/15 (6.67 %) | 16/17 (94.12 %) | 18/54 (33.33 %) |
| **Outcome after drug withdrawal** | Evolution Unknown: 1/1 (100.0 %) | Recovered: 1/16 (6.25 %) Not recovered: 1/16 (6.25 %) Evolution Unknown: 4/16 (25.0 %) | Recovered: 9/18 (50.0 %) Recovered with sequelae: 1/18 (5.56 %) Not recovered: 7/18 (38.89 %) |
| **Country of primary source** | France: 7/15 (46.7 %) United Kingdom of Great Britain and Northern Ireland: 3/15 (20.0 %) United States of America: 2/15 (13.3 %) Spain: 2/15 (13.3 %) Australia: 1/15 (6.7 %) | United States of America: 17/17 (100.0 %) | France: 17/54 (31.5 %) United States of America: 11/54 (20.4 %) United Kingdom of Great Britain and Northern Ireland: 6/54 (11.1 %) Netherlands: 5/54 (9.3 %) Belgium: 2/54 (3.7 %) |
| **Co-reported drugs** | Metoprolol tartrate: 1/ 15 (6.7 %) Allopurinol: 1/ 15 (6.7 %) Phenoxymethylpenicillin: 1/ 15 (6.7 %) Buclizine hydrochloride;Codeine phosphate;Paracetamol: 1/ 15 (6.7 %) Chlorphenamine: 1/ 15 (6.7 %) | Amfetamine aspartate;Amfetamine sulfate;Dexamfetamine saccharate;Dexamfetamine sulfate: 5/ 17 (29.4 %) Solriamfetol hydrochloride: 4/ 17 (23.5 %) Solriamfetol: 4/ 17 (23.5 %) Ondansetron: 4/ 17 (23.5 %) Ondansetron hydrochloride: 4/ 17 (23.5 %) | Erenumab: 6/ 54 (11.1 %) Galcanezumab: 4/ 54 (7.4 %) Rizatriptan: 4/ 54 (7.4 %) Paracetamol: 3/ 54 (5.6 %) Zolmitriptan: 3/ 54 (5.6 %) |
| **Co-reported reactions** | Malignant neoplasm progression: 1/15 (6.7 %) Coombs direct test positive: 1/15 (6.7 %) Autoimmune haemolytic anaemia: 1/15 (6.7 %) Arthralgia: 1/15 (6.7 %) Rheumatoid factor positive: 1/15 (6.7 %) | Depression: 5/17 (29.4 %) Product administration interrupted: 4/17 (23.5 %) Migraine: 4/17 (23.5 %) Intentional dose omission: 4/17 (23.5 %) Neuropathy peripheral: 4/17 (23.5 %) | Peripheral coldness: 5/54 (9.3 %) Headache: 4/54 (7.4 %) Nightmare: 4/54 (7.4 %) Dizziness: 4/54 (7.4 %) Malaise: 3/54 (5.6 %) |
| **Reporter qualification** | Healthcare professional : 11/15 (73.33 %) Non healthcare professional : 4/15 (26.67 %) | Healthcare professional : 7/17 (41.18 %) Non healthcare professional : 10/17 (58.82 %) | Healthcare professional : 35/54 (64.81 %) Non healthcare professional : 19/54 (35.19 %) |
| **Indications** | Unknown : 11/15 (73.3 %) Chronic myeloid leukaemia : 2/15 (13.3 %) Renal cell carcinoma : 1/15 (6.7 %) Hepatitis in viral disease classified elsewhere : 1/15 (6.7 %) | Narcolepsy : 11/17 (64.7 %) Cataplexy : 10/17 (58.8 %) Somnolence : 9/17 (52.9 %) Unknown : 6/17 (35.3 %) Hypersomnia : 4/17 (23.5 %) | Unknown : 11/54 (20.4 %) Migraine : 8/54 (14.8 %) Product used for unknown indication : 3/54 (5.6 %) Migraine with aura : 2/54 (3.7 %) Tachycardia : 2/54 (3.7 %) |
| **Mean Time to Onset (std)** | 347 days (337 days) | nan | 689 days (1186 days) |

|  | **Amfetamine;Dexamfetamine** | **Alendronic acid** | **Rizatriptan** |
| --- | --- | --- | --- |
| **Number of cases** | 36 | 79 | 12 |
| **IC025_RP_main** | 3.05 | 2.89 | 2.7 |
| **IC025_RP_comparator** | 0.14 | 1.58 | 0.97 |
| **IC025_RP_healthcare** | 2.94 | 2.89 | 3.21 |
| **IC025_RP_malefemale** | 2.42 | 2.31 | 2.34 |
| **IC025_RP-Broad_main** | 3.73 | 2.65 | 3.98 |
| **Sex (Female / Male / Unknown)** | 28 / 4 / 4 | 71 / 3 / 5 | 12 / 0 / 0 |
| **Mean age (SD) in years** | 35.17 (18.52) years | 59.11 (11.44) years | 35.67 (13.18) years |
| **Mean number of suspect/interacting drugs (SD)** | 5.56 (4.98) | 4.4 (3.94) | 6.36 (4.41) |
| **Withdrawn** | 5/36 (13.89 %) | 11/79 (13.92 %) | 2/12 (16.67 %) |
| **Outcome after drug withdrawal** | Recovered: 1/5 (20.0 %) Not recovered: 2/5 (40.0 %) Evolution Unknown: 2/5 (40.0 %) | Recovered: 2/11 (18.18 %) Evolution Unknown: 5/11 (45.45 %) | Recovered: 1/2 (50.0 %) |
| **Country of primary source** | United States of America: 34/36 (94.4 %) Canada: 2/36 (5.6 %) | United States of America: 72/79 (91.1 %) United Kingdom of Great Britain and Northern Ireland: 2/79 (2.5 %) Canada: 2/79 (2.5 %) Japan: 1/79 (1.3 %) Spain: 1/79 (1.3 %) | United States of America: 10/12 (83.3 %) France: 1/12 (8.3 %) Germany: 1/12 (8.3 %) |
| **Co-reported drugs** | Oxybate sodium: 9/ 36 (25.0 %) Lisdexamfetamine mesilate: 7/ 36 (19.4 %) Methylphenidate: 5/ 36 (13.9 %) Methylphenidate hydrochloride: 5/ 36 (13.9 %) Bupropion: 4/ 36 (11.1 %) | Calcium: 19/ 79 (24.1 %) Risedronate sodium: 12/ 79 (15.2 %) Vitamin d nos: 11/ 79 (13.9 %) Alendronate sodium;Colecalciferol: 11/ 79 (13.9 %) Corticosteroids: 9/ 79 (11.4 %) | Erenumab: 8/ 12 (66.7 %) Nortriptyline: 5/ 12 (41.7 %) Topiramate: 5/ 12 (41.7 %) Nadolol: 4/ 12 (33.3 %) Propranolol: 4/ 12 (33.3 %) |
| **Co-reported reactions** | Drug ineffective: 8/36 (22.2 %) Headache: 6/36 (16.7 %) Nausea: 5/36 (13.9 %) Prescribed overdose: 4/36 (11.1 %) Pain: 4/36 (11.1 %) | Femur fracture: 46/79 (58.2 %) Osteoarthritis: 44/79 (55.7 %) Hypertension: 37/79 (46.8 %) Fall: 36/79 (45.6 %) Anxiety: 33/79 (41.8 %) | Sedation: 1/12 (8.3 %) Drug ineffective: 1/12 (8.3 %) Drug interaction: 1/12 (8.3 %) Skin exfoliation: 1/12 (8.3 %) Gangrene: 1/12 (8.3 %) |
| **Reporter qualification** | Healthcare professional : 17/36 (47.22 %) Non healthcare professional : 19/36 (52.78 %) | Healthcare professional : 48/79 (60.76 %) Non healthcare professional : 31/79 (39.24 %) | Healthcare professional : 12/12 (100.0 %) Non healthcare professional : 0/12 (0.0 %) |
| **Indications** | Attention deficit/hyperactivity disorder : 13/36 (36.1 %) Product used for unknown indication : 11/36 (30.6 %) Attention deficit hyperactivity disorder : 5/36 (13.9 %) Unknown : 4/36 (11.1 %) Somnolence : 2/36 (5.6 %) | Osteoporosis : 59/79 (74.7 %) Unknown : 45/79 (57.0 %) Osteopenia : 27/79 (34.2 %) Senile osteoporosis : 3/79 (3.8 %) Osteoporosis prophylaxis : 3/79 (3.8 %) | Migraine : 8/12 (66.7 %) Migraine without aura : 3/12 (25.0 %) Migraine with aura : 3/12 (25.0 %) Product used for unknown indication : 2/12 (16.7 %) |
| **Mean Time to Onset (std)** | nan | 1320 days (1135 days) | 70 days (98 days) |

|  | **Carteolol** | **Celiprolol** | **Metoprolol** |
| --- | --- | --- | --- |
| **Number of cases** | 8 | 9 | 61 |
| **IC025_RP_main** | 2.61 | 2.57 | 2.74 |
| **IC025_RP_comparator** | 1.39 | 0.39 | 0.12 |
| **IC025_RP_healthcare** | 2.11 | 2.16 | 2.07 |
| **IC025_RP_malefemale** | 2.69 | 2.71 | 2.92 |
| **IC025_RP-Broad_main** | 2.48 | 5.14 | 4.57 |
| **Sex (Female / Male / Unknown)** | 2 / 6 / 0 | 3 / 6 / 0 | 40 / 20 / 1 |
| **Mean age (SD) in years** | 65.75 (10.47) years | 57.44 (14.59) years | 58.51 (14.5) years |
| **Mean number of suspect/interacting drugs (SD)** | 1.67 (1.15) | 1.0 (0.0) | 5.53 (7.12) |
| **Withdrawn** | 3/8 (37.5 %) | 1/9 (11.11 %) | 26/61 (42.62 %) |
| **Outcome after drug withdrawal** | Recovering: 1/3 (33.33 %) Not recovered: 1/3 (33.33 %) Evolution Unknown: 1/3 (33.33 %) | Recovered: 1/1 (100.0 %) | Recovered: 6/26 (23.08 %) Recovering: 3/26 (11.54 %) Not recovered: 3/26 (11.54 %) Evolution Unknown: 12/26 (46.15 %) |
| **Country of primary source** | France: 8/8 (100.0 %) | France: 7/9 (77.8 %) United Kingdom of Great Britain and Northern Ireland: 2/9 (22.2 %) | United States of America: 29/61 (47.5 %) Netherlands: 13/61 (21.3 %) France: 3/61 (4.9 %) Germany: 3/61 (4.9 %) Sweden: 3/61 (4.9 %) |
| **Co-reported drugs** | Febuxostat: 1/ 8 (12.5 %) Acebutolol hydrochloride: 1/ 8 (12.5 %) Latanoprost;Timolol maleate: 1/ 8 (12.5 %) Latanoprost: 1/ 8 (12.5 %) Dorzolamide hydrochloride: 1/ 8 (12.5 %) | Lercanidipine hydrochloride: 1/ 9 (11.1 %) Topiramate: 1/ 9 (11.1 %) Lorazepam: 1/ 9 (11.1 %) | Acetylsalicylic acid: 12/ 61 (19.7 %) Rosuvastatin: 6/ 61 (9.8 %) Rosuvastatin calcium: 6/ 61 (9.8 %) Lisinopril: 6/ 61 (9.8 %) Prednisone: 5/ 61 (8.2 %) |
| **Co-reported reactions** | Treatment noncompliance: 2/8 (25.0 %) Dry eye: 1/8 (12.5 %) Ageusia: 1/8 (12.5 %) Oedema peripheral: 1/8 (12.5 %) Pain in extremity: 1/8 (12.5 %) | Asthenia: 1/9 (11.1 %) Muscle spasms: 1/9 (11.1 %) Peripheral coldness: 1/9 (11.1 %) Pain: 1/9 (11.1 %) | Peripheral coldness: 8/61 (13.1 %) Dyspnoea: 7/61 (11.5 %) Dizziness: 7/61 (11.5 %) Fatigue: 7/61 (11.5 %) Pain in extremity: 6/61 (9.8 %) |
| **Reporter qualification** | Healthcare professional : 6/8 (75.0 %) Non healthcare professional : 2/8 (25.0 %) | Healthcare professional : 7/9 (77.78 %) Non healthcare professional : 2/9 (22.22 %) | Healthcare professional : 25/61 (40.98 %) Non healthcare professional : 36/61 (59.02 %) |
| **Indications** | Glaucoma : 3/8 (37.5 %) Unknown : 2/8 (25.0 %) Open angle glaucoma : 1/8 (12.5 %) Prophylaxis : 1/8 (12.5 %) Hypertension ocular : 1/8 (12.5 %) | Unknown : 8/9 (88.9 %) Essential hypertension, benign : 1/9 (11.1 %) | Unknown : 18/61 (29.5 %) Hypertension : 16/61 (26.2 %) Product used for unknown indication : 4/61 (6.6 %) Acute myocardial infarction : 3/61 (4.9 %) Atrial fibrillation : 3/61 (4.9 %) |
| **Mean Time to Onset (std)** | 409 days (536 days) | 173 days (275 days) | 496 days (845 days) |

|  | **Interferon alfa-2b** | **Amifampridine** | **Ergotamine** |
| --- | --- | --- | --- |
| **Number of cases** | 24 | 8 | 6 |
| **IC025_RP_main** | 2.45 | 2.33 | 2.11 |
| **IC025_RP_comparator** | 2.43 | 1.38 | 1.37 |
| **IC025_RP_healthcare** | 2.46 | 2.19 | 1.74 |
| **IC025_RP_malefemale** | 2.28 | 2.29 | 2.09 |
| **IC025_RP-Broad_main** | 2.26 | 1.77 | 5.45 |
| **Sex (Female / Male / Unknown)** | 15 / 8 / 1 | 8 / 0 / 0 | 5 / 1 / 0 |
| **Mean age (SD) in years** | 51.39 (15.93) years | 58.14 (12.35) years | 40.0 (10.79) years |
| **Mean number of suspect/interacting drugs (SD)** | 2.31 (1.75) | 4.0 (3.74) | 2.8 (1.92) |
| **Withdrawn** | 2/24 (8.33 %) | 5/8 (62.5 %) | 3/6 (50.0 %) |
| **Outcome after drug withdrawal** | Recovering: 1/2 (50.0 %) Recovered with sequelae: 1/2 (50.0 %) | Recovered: 1/5 (20.0 %) Not recovered: 1/5 (20.0 %) Evolution Unknown: 2/5 (40.0 %) | Recovered: 1/3 (33.33 %) Recovering: 1/3 (33.33 %) Not recovered: 1/3 (33.33 %) |
| **Country of primary source** | United States of America: 9/24 (37.5 %) France: 7/24 (29.2 %) Canada: 3/24 (12.5 %) Netherlands: 2/24 (8.3 %) Australia: 1/24 (4.2 %) | France: 5/8 (62.5 %) United States of America: 2/8 (25.0 %) Netherlands: 1/8 (12.5 %) | France: 4/6 (66.7 %) United States of America: 1/6 (16.7 %) Thailand: 1/6 (16.7 %) |
| **Co-reported drugs** | Ribavirin: 5/ 24 (20.8 %) Acetylsalicylate lysine: 2/ 24 (8.3 %) Acetylsalicylic acid: 2/ 24 (8.3 %) Hydroxycarbamide: 2/ 24 (8.3 %) Levothyroxine sodium: 2/ 24 (8.3 %) | Pyridostigmine bromide: 2/ 8 (25.0 %) Baclofen: 2/ 8 (25.0 %) Zolpidem tartrate: 1/ 8 (12.5 %) Carvedilol: 1/ 8 (12.5 %) Amantadine: 1/ 8 (12.5 %) | Ritonavir: 2/ 6 (33.3 %) Saquinavir: 2/ 6 (33.3 %) Propranolol hydrochloride: 2/ 6 (33.3 %) Propranolol: 2/ 6 (33.3 %) Abacavir;Lamivudine: 1/ 6 (16.7 %) |
| **Co-reported reactions** | Systemic lupus erythematosus: 3/24 (12.5 %) Alopecia: 2/24 (8.3 %) Skin ulcer: 2/24 (8.3 %) Product use in unapproved indication: 1/24 (4.2 %) Erythema: 1/24 (4.2 %) | Paraesthesia: 6/8 (75.0 %) Paraesthesia oral: 3/8 (37.5 %) Limb discomfort: 2/8 (25.0 %) Burning sensation: 2/8 (25.0 %) Muscular weakness: 2/8 (25.0 %) | Ergot poisoning: 2/6 (33.3 %) Arterial thrombosis: 1/6 (16.7 %) Drug interaction: 1/6 (16.7 %) Vasculitis: 1/6 (16.7 %) Embolism: 1/6 (16.7 %) |
| **Reporter qualification** | Healthcare professional : 18/24 (75.0 %) Non healthcare professional : 6/24 (25.0 %) | Healthcare professional : 5/8 (62.5 %) Non healthcare professional : 3/8 (37.5 %) | Healthcare professional : 5/6 (83.33 %) Non healthcare professional : 1/6 (16.67 %) |
| **Indications** | Unknown : 17/24 (70.8 %) Malignant melanoma of skin, unspecified : 2/24 (8.3 %) Mycosis fungoides : 1/24 (4.2 %) Essential thrombocythaemia : 1/24 (4.2 %) T-cell lymphoma : 1/24 (4.2 %) | Unknown : 3/8 (37.5 %) Myasthenic syndrome : 2/8 (25.0 %) Lambert-Eaton myasthenic syndrome : 1/8 (12.5 %) Myasthenia gravis : 1/8 (12.5 %) Asthenia : 1/8 (12.5 %) | Migraine : 2/6 (33.3 %) Unknown : 2/6 (33.3 %) Classical migraine : 1/6 (16.7 %) Migraine, unspecified : 1/6 (16.7 %) |
| **Mean Time to Onset (std)** | 181 days (160 days) | 180 days (354 days) | 8 days (9 days) |

|  | **Immunoglobulin g human** | **Alendronic acid;Colecalciferol** | **Sotalol** |
| --- | --- | --- | --- |
| **Number of cases** | 14 | 11 | 12 |
| **IC025_RP_main** | 2.21 | 2.17 | 2.28 |
| **IC025_RP_comparator** | 1.61 | 1.19 | -0.66 |
| **IC025_RP_healthcare** | 1.92 | 1.82 | 1.47 |
| **IC025_RP_malefemale** | 1.71 | 0.45 | 2.12 |
| **IC025_RP-Broad_main** | 1.66 | 1.85 | 4.06 |
| **Sex (Female / Male / Unknown)** | 14 / 0 / 0 | 9 / 0 / 2 | 7 / 5 / 0 |
| **Mean age (SD) in years** | 60.89 (10.12) years | 55.0 (5.6) years | 69.78 (11.99) years |
| **Mean number of suspect/interacting drugs (SD)** | 12.0 (12.89) | 5.45 (3.98) | 4.83 (3.97) |
| **Withdrawn** | 1/14 (7.14 %) | 1/11 (9.09 %) | 2/12 (16.67 %) |
| **Outcome after drug withdrawal** | Evolution Unknown: 1/1 (100.0 %) | Evolution Unknown: 1/1 (100.0 %) | Recovered: 1/2 (50.0 %) Evolution Unknown: 1/2 (50.0 %) |
| **Country of primary source** | United States of America: 14/14 (100.0 %) | United States of America: 11/11 (100.0 %) | France: 6/12 (50.0 %) Netherlands: 3/12 (25.0 %) Belgium: 1/12 (8.3 %) Sweden: 1/12 (8.3 %) United States of America: 1/12 (8.3 %) |
| **Co-reported drugs** | Diphenhydramine: 9/ 14 (64.3 %) Diphenhydramine hydrochloride: 9/ 14 (64.3 %) Immunoglobulin human normal: 5/ 14 (35.7 %) Salbutamol: 5/ 14 (35.7 %) Salbutamol sulfate: 5/ 14 (35.7 %) | Alendronate sodium: 11/ 11 (100.0 %) Calcium: 5/ 11 (45.5 %) Corticosteroids: 3/ 11 (27.3 %) Vitamin d nos: 3/ 11 (27.3 %) Levothyroxine sodium: 3/ 11 (27.3 %) | Zolpidem tartrate: 2/ 12 (16.7 %) Estriol: 1/ 12 (8.3 %) Paracetamol: 1/ 12 (8.3 %) Macrogol 3350;Potassium chloride;Sodium bicarbonate;Sodium chloride: 1/ 12 (8.3 %) Levothyroxine sodium: 1/ 12 (8.3 %) |
| **Co-reported reactions** | Fatigue: 9/14 (64.3 %) Headache: 7/14 (50.0 %) Malaise: 6/14 (42.9 %) Illness: 5/14 (35.7 %) COVID-19: 5/14 (35.7 %) | Femur fracture: 9/11 (81.8 %) Fall: 7/11 (63.6 %) Low turnover osteopathy: 7/11 (63.6 %) Osteoarthritis: 7/11 (63.6 %) Anxiety: 6/11 (54.5 %) | Skin discolouration: 2/12 (16.7 %) Pain in extremity: 2/12 (16.7 %) Peripheral coldness: 2/12 (16.7 %) Palpitations: 1/12 (8.3 %) Visual acuity reduced: 1/12 (8.3 %) |
| **Reporter qualification** | Healthcare professional : 11/14 (78.57 %) Non healthcare professional : 3/14 (21.43 %) | Healthcare professional : 8/11 (72.73 %) Non healthcare professional : 3/11 (27.27 %) | Healthcare professional : 7/12 (58.33 %) Non healthcare professional : 5/12 (41.67 %) |
| **Indications** | Primary immunodeficiency syndrome : 9/14 (64.3 %) Immunodeficiency common variable : 8/14 (57.1 %) Unknown : 3/14 (21.4 %) Hypogammaglobulinaemia : 2/14 (14.3 %) Selective IgG subclass deficiency : 2/14 (14.3 %) | Osteoporosis : 7/11 (63.6 %) Senile osteoporosis : 1/11 (9.1 %) Unknown : 1/11 (9.1 %) Osteopenia : 1/11 (9.1 %) Postmenopause : 1/11 (9.1 %) | Unknown : 5/12 (41.7 %) Atrial fibrillation : 3/12 (25.0 %) Arrhythmia NOS : 1/12 (8.3 %) Fibrillation atrial : 1/12 (8.3 %) Insufficiency cardiac : 1/12 (8.3 %) |
| **Mean Time to Onset (std)** | nan | 167 days (244 days) | 159 days (161 days) |

|  | **Atenolol** | **Belimumab** | **Zolmitriptan** |
| --- | --- | --- | --- |
| **Number of cases** | 30 | 24 | 9 |
| **IC025_RP_main** | 2.27 | 2.16 | 2.03 |
| **IC025_RP_comparator** | -1.11 | 1.78 | 0.24 |
| **IC025_RP_healthcare** | 2.19 | 2.47 | 2.01 |
| **IC025_RP_malefemale** | 2.23 | 1.51 | 1.85 |
| **IC025_RP-Broad_main** | 5.02 | 1.45 | 2.96 |
| **Sex (Female / Male / Unknown)** | 18 / 12 / 0 | 18 / 1 / 5 | 8 / 1 / 0 |
| **Mean age (SD) in years** | 52.7 (15.09) years | 42.75 (8.35) years | 41.25 (8.73) years |
| **Mean number of suspect/interacting drugs (SD)** | 4.94 (3.38) | 6.44 (2.5) | 12.8 (16.65) |
| **Withdrawn** | 14/30 (46.67 %) | 6/24 (25.0 %) | 3/9 (33.33 %) |
| **Outcome after drug withdrawal** | Recovered: 4/14 (28.57 %) Recovering: 1/14 (7.14 %) Recovered with sequelae: 1/14 (7.14 %) Not recovered: 3/14 (21.43 %) Evolution Unknown: 2/14 (14.29 %) | Recovering: 1/6 (16.67 %) Not recovered: 1/6 (16.67 %) Evolution Unknown: 2/6 (33.33 %) | Recovered: 1/3 (33.33 %) Not recovered: 1/3 (33.33 %) Evolution Unknown: 1/3 (33.33 %) |
| **Country of primary source** | France: 14/30 (46.7 %) United States of America: 9/30 (30.0 %) Brazil: 1/30 (3.3 %) Spain: 1/30 (3.3 %) Italy: 1/30 (3.3 %) | United States of America: 13/24 (54.2 %) Canada: 6/24 (25.0 %) Korea (the Republic of): 2/24 (8.3 %) Colombia: 1/24 (4.2 %) France: 1/24 (4.2 %) | United States of America: 5/9 (55.6 %) France: 3/9 (33.3 %) United Kingdom of Great Britain and Northern Ireland: 1/9 (11.1 %) |
| **Co-reported drugs** | Simvastatin: 4/ 30 (13.3 %) Naproxen: 3/ 30 (10.0 %) Acetylsalicylate lysine: 3/ 30 (10.0 %) Acetylsalicylic acid: 3/ 30 (10.0 %) Metoprolol: 3/ 30 (10.0 %) | Hydroxychloroquine sulfate: 11/ 24 (45.8 %) Hydroxychloroquine: 11/ 24 (45.8 %) Methotrexate: 6/ 24 (25.0 %) Prednisone: 6/ 24 (25.0 %) Prednisolone: 4/ 24 (16.7 %) | Nadolol: 2/ 9 (22.2 %) Rizatriptan: 2/ 9 (22.2 %) Erenumab: 2/ 9 (22.2 %) Salbutamol sulfate: 2/ 9 (22.2 %) Salbutamol: 2/ 9 (22.2 %) |
| **Co-reported reactions** | Hypoaesthesia: 3/30 (10.0 %) Ventricular extrasystoles: 2/30 (6.7 %) Anxiety: 2/30 (6.7 %) Hyperhidrosis: 2/30 (6.7 %) Peripheral swelling: 2/30 (6.7 %) | Fatigue: 12/24 (50.0 %) Pain: 8/24 (33.3 %) Malaise: 6/24 (25.0 %) Arthralgia: 6/24 (25.0 %) Drug ineffective: 6/24 (25.0 %) | Overdose: 1/9 (11.1 %) Dyspnoea: 1/9 (11.1 %) Blood potassium abnormal: 1/9 (11.1 %) Sinusitis: 1/9 (11.1 %) Sinus disorder: 1/9 (11.1 %) |
| **Reporter qualification** | Healthcare professional : 22/30 (73.33 %) Non healthcare professional : 8/30 (26.67 %) | Healthcare professional : 12/24 (50.0 %) Non healthcare professional : 12/24 (50.0 %) | Healthcare professional : 7/9 (77.78 %) Non healthcare professional : 2/9 (22.22 %) |
| **Indications** | Unknown : 9/30 (30.0 %) Hypertension : 3/30 (10.0 %) Migraine : 3/30 (10.0 %) Hypertension arterial : 3/30 (10.0 %) Migraine with aura : 2/30 (6.7 %) | Systemic lupus erythematosus : 15/24 (62.5 %) Product used for unknown indication : 9/24 (37.5 %) Unknown : 4/24 (16.7 %) Systemic lupus erythematosis : 2/24 (8.3 %) Arthritis : 1/24 (4.2 %) | Unknown : 5/9 (55.6 %) Migraine without aura : 2/9 (22.2 %) Tension headache : 1/9 (11.1 %) Migraine : 1/9 (11.1 %) |
| **Mean Time to Onset (std)** | 859 days (1961 days) | 343 days (522 days) | 128 days (110 days) |

|  | **Rimegepant** | **Dexmethylphenidate** | **Milnacipran** |
| --- | --- | --- | --- |
| **Number of cases** | 12 | 7 | 9 |
| **IC025_RP_main** | 2.04 | 1.93 | 1.94 |
| **IC025_RP_comparator** | -0.0 | -0.98 | 2.08 |
| **IC025_RP_healthcare** | 2.14 | 1.55 | 0.96 |
| **IC025_RP_malefemale** | 0.87 | 1.94 | 1.89 |
| **IC025_RP-Broad_main** | 1.61 | 1.8 | 2.49 |
| **Sex (Female / Male / Unknown)** | 8 / 0 / 4 | 1 / 6 / 0 | 7 / 2 / 0 |
| **Mean age (SD) in years** | 48.0 (14.85) years | 11.5 (2.07) years | 50.25 (17.27) years |
| **Mean number of suspect/interacting drugs (SD)** | 2.67 (2.66) | 3.0 (2.31) | 2.4 (1.52) |
| **Withdrawn** | 0/12 (0.0 %) | 4/7 (57.14 %) | 2/9 (22.22 %) |
| **Outcome after drug withdrawal** |  | Recovered: 1/4 (25.0 %) Not recovered: 1/4 (25.0 %) Evolution Unknown: 2/4 (50.0 %) | Evolution Unknown: 2/2 (100.0 %) |
| **Country of primary source** | United States of America: 10/12 (83.3 %) France: 1/12 (8.3 %) Lithuania: 1/12 (8.3 %) | United States of America: 6/7 (85.7 %) Switzerland: 1/7 (14.3 %) | United States of America: 4/9 (44.4 %) Germany: 2/9 (22.2 %) France: 2/9 (22.2 %) Mexico: 1/9 (11.1 %) |
| **Co-reported drugs** | Galcanezumab gnlm: 3/ 12 (25.0 %) Nortriptyline: 2/ 12 (16.7 %) Metformin: 1/ 12 (8.3 %) Spironolactone: 1/ 12 (8.3 %) Phentermine: 1/ 12 (8.3 %) | Methylphenidate hydrochloride: 4/ 7 (57.1 %) Quetiapine fumarate: 2/ 7 (28.6 %) Aripiprazole: 2/ 7 (28.6 %) Amfetamine aspartate;Amfetamine sulfate;Dexamfetamine saccharate;Dexamfetamine sulfate: 1/ 7 (14.3 %) Carbamazepine: 1/ 7 (14.3 %) | Zolpidem tartrate: 2/ 9 (22.2 %) Quetiapine: 1/ 9 (11.1 %) Olanzapine: 1/ 9 (11.1 %) Simvastatin: 1/ 9 (11.1 %) Acetylsalicylic acid: 1/ 9 (11.1 %) |
| **Co-reported reactions** | Skin discolouration: 3/12 (25.0 %) Drug ineffective: 2/12 (16.7 %) Peripheral coldness: 1/12 (8.3 %) Feeling cold: 1/12 (8.3 %) Paraesthesia: 1/12 (8.3 %) | Skin discolouration: 4/7 (57.1 %) Erythema: 3/7 (42.9 %) Feeling cold: 2/7 (28.6 %) Skin abrasion: 2/7 (28.6 %) Paraesthesia: 2/7 (28.6 %) | Collagen disorder: 1/9 (11.1 %) Somnolence: 1/9 (11.1 %) Paraesthesia: 1/9 (11.1 %) Peripheral coldness: 1/9 (11.1 %) Abdominal pain upper: 1/9 (11.1 %) |
| **Reporter qualification** | Healthcare professional : 7/12 (58.33 %) Non healthcare professional : 5/12 (41.67 %) | Healthcare professional : 5/7 (71.43 %) Non healthcare professional : 2/7 (28.57 %) | Healthcare professional : 5/9 (55.56 %) Non healthcare professional : 4/9 (44.44 %) |
| **Indications** | Migraine : 8/12 (66.7 %) Unknown : 3/12 (25.0 %) Migraine prophylaxis : 1/12 (8.3 %) Prophylaxis : 1/12 (8.3 %) | Attention deficit/hyperactivity disorder : 6/7 (85.7 %) Unknown : 2/7 (28.6 %) Attention deficit hyperactivity disorder : 1/7 (14.3 %) | Fibromyalgia : 4/9 (44.4 %) Unknown : 2/9 (22.2 %) Depression : 1/9 (11.1 %) Depressive symptom : 1/9 (11.1 %) Drug use for unknown indication : 1/9 (11.1 %) |
| **Mean Time to Onset (std)** | nan | nan | 6 days (6 days) |

|  | **Sumatriptan** | **Nadolol** | **Solriamfetol** |
| --- | --- | --- | --- |
| **Number of cases** | 31 | 7 | 5 |
| **IC025_RP_main** | 1.95 | 1.86 | 1.33 |
| **IC025_RP_comparator** | -0.42 | -0.58 | -1.42 |
| **IC025_RP_healthcare** | 2.05 | 1.88 | -2.55 |
| **IC025_RP_malefemale** | 1.95 | 0.89 | -2.44 |
| **IC025_RP-Broad_main** | 2.93 | 4.48 | 2.52 |
| **Sex (Female / Male / Unknown)** | 25 / 4 / 2 | 6 / 0 / 1 | 4 / 0 / 1 |
| **Mean age (SD) in years** | 41.72 (10.04) years | 51.0 (20.83) years | 49.0 (nan) years |
| **Mean number of suspect/interacting drugs (SD)** | 4.75 (4.56) | 5.5 (5.24) | 4.5 (5.74) |
| **Withdrawn** | 11/31 (35.48 %) | 1/7 (14.29 %) | 0/5 (0.0 %) |
| **Outcome after drug withdrawal** | Recovered: 2/11 (18.18 %) Not recovered: 5/11 (45.45 %) Evolution Unknown: 4/11 (36.36 %) | Recovered: 1/1 (100.0 %) |  |
| **Country of primary source** | United States of America: 18/31 (58.1 %) United Kingdom of Great Britain and Northern Ireland: 6/31 (19.4 %) Germany: 3/31 (9.7 %) France: 2/31 (6.5 %) Norway: 1/31 (3.2 %) | United States of America: 6/7 (85.7 %) France: 1/7 (14.3 %) | United States of America: 5/5 (100.0 %) |
| **Co-reported drugs** | Paracetamol: 4/ 31 (12.9 %) Naproxen: 4/ 31 (12.9 %) Naproxen sodium: 4/ 31 (12.9 %) Erenumab: 3/ 31 (9.7 %) Magnesium: 3/ 31 (9.7 %) | Rizatriptan: 4/ 7 (57.1 %) Erenumab: 4/ 7 (57.1 %) Magnesium: 3/ 7 (42.9 %) Zolmitriptan: 2/ 7 (28.6 %) Propranolol: 2/ 7 (28.6 %) | Methylphenidate hydrochloride: 1/ 5 (20.0 %) Minerals nos;Vitamins nos: 1/ 5 (20.0 %) Xantofyl: 1/ 5 (20.0 %) Ferrous sulfate: 1/ 5 (20.0 %) Fish oil: 1/ 5 (20.0 %) |
| **Co-reported reactions** | Paraesthesia: 4/31 (12.9 %) Hypoaesthesia: 4/31 (12.9 %) Peripheral coldness: 4/31 (12.9 %) Pain: 3/31 (9.7 %) Ventricular tachycardia: 3/31 (9.7 %) | Prinzmetal angina: 1/7 (14.3 %) Bradycardia: 1/7 (14.3 %) Electrocardiogram T wave abnormal: 1/7 (14.3 %) Hyperhidrosis: 1/7 (14.3 %) Ventricular tachycardia: 1/7 (14.3 %) | Depression: 2/5 (40.0 %) Hallucination, auditory: 1/5 (20.0 %) Arrhythmia: 1/5 (20.0 %) Weight increased: 1/5 (20.0 %) Asthma exercise induced: 1/5 (20.0 %) |
| **Reporter qualification** | Healthcare professional : 16/31 (51.61 %) Non healthcare professional : 15/31 (48.39 %) | Healthcare professional : 6/7 (85.71 %) Non healthcare professional : 1/7 (14.29 %) | Healthcare professional : 1/5 (20.0 %) Non healthcare professional : 4/5 (80.0 %) |
| **Indications** | Migraine : 22/31 (71.0 %) Unknown : 4/31 (12.9 %) Migraine with aura : 3/31 (9.7 %) Product used for unknown indication : 2/31 (6.5 %) Migraine without aura : 1/31 (3.2 %) | Unknown : 3/7 (42.9 %) Migraine without aura : 2/7 (28.6 %) Product used for unknown indication : 2/7 (28.6 %) | Product used for unknown indication : 4/5 (80.0 %) Somnolence : 1/5 (20.0 %) |
| **Mean Time to Onset (std)** | 446 days (870 days) | nan | nan |

|  | **Covid-19 vaccine** | **Bromocriptine** | **Voclosporin** |
| --- | --- | --- | --- |
| **Number of cases** | 15 | 9 | 4 |
| **IC025_RP_main** | 1.37 | 1.69 | 0.38 |
| **IC025_RP_comparator** | -0.64 | 1.79 | 0.13 |
| **IC025_RP_healthcare** | 1.21 | 0.24 | -0.53 |
| **IC025_RP_malefemale** | 1.4 | 1.2 | -0.91 |
| **IC025_RP-Broad_main** | 2.42 | 2.92 | -0.42 |
| **Sex (Female / Male / Unknown)** | 12 / 3 / 0 | 7 / 2 / 0 | 2 / 2 / 0 |
| **Mean age (SD) in years** | 55.85 (18.96) years | 44.86 (14.21) years | 32.0 (4.24) years |
| **Mean number of suspect/interacting drugs (SD)** | 4.13 (7.52) | 1.0 (0.0) | 3.0 (2.83) |
| **Withdrawn** | 1/15 (6.67 %) | 1/9 (11.11 %) | 2/4 (50.0 %) |
| **Outcome after drug withdrawal** | Not recovered: 1/1 (100.0 %) | Recovered: 1/1 (100.0 %) | Evolution Unknown: 1/2 (50.0 %) |
| **Country of primary source** | United States of America: 4/15 (26.7 %) Italy: 3/15 (20.0 %) United Kingdom of Great Britain and Northern Ireland: 2/15 (13.3 %) France: 2/15 (13.3 %) Germany: 2/15 (13.3 %) | France: 3/9 (33.3 %) United Kingdom of Great Britain and Northern Ireland: 3/9 (33.3 %) Switzerland: 1/9 (11.1 %) Belgium: 1/9 (11.1 %) Sweden: 1/9 (11.1 %) | United States of America: 4/4 (100.0 %) |
| **Co-reported drugs** | Acetylsalicylic acid: 3/ 15 (20.0 %) Gabapentin: 2/ 15 (13.3 %) Doxazosin: 2/ 15 (13.3 %) Morphine: 2/ 15 (13.3 %) Morphine sulfate: 2/ 15 (13.3 %) | Benserazide hydrochloride;Levodopa: 1/ 9 (11.1 %) Dextropropoxyphene hydrochloride;Paracetamol: 1/ 9 (11.1 %) | Hydroxychloroquine sulfate: 1/ 4 (25.0 %) Belimumab: 1/ 4 (25.0 %) Colecalciferol: 1/ 4 (25.0 %) Losartan: 1/ 4 (25.0 %) Mycophenolate mofetil: 1/ 4 (25.0 %) |
| **Co-reported reactions** | Arthralgia: 5/15 (33.3 %) COVID-19: 4/15 (26.7 %) Fatigue: 4/15 (26.7 %) Nasopharyngitis: 3/15 (20.0 %) Pain in extremity: 3/15 (20.0 %) | Constipation: 1/9 (11.1 %) Malaise: 1/9 (11.1 %) Headache: 1/9 (11.1 %) Ejaculation disorder: 1/9 (11.1 %) Vasospasm: 1/9 (11.1 %) | Arthralgia: 3/4 (75.0 %) Nephritis: 1/4 (25.0 %) White blood cell count increased: 1/4 (25.0 %) Insurance issue: 1/4 (25.0 %) Therapy interrupted: 1/4 (25.0 %) |
| **Reporter qualification** | Healthcare professional : 8/15 (53.33 %) Non healthcare professional : 7/15 (46.67 %) | Healthcare professional : 4/9 (44.44 %) Non healthcare professional : 5/9 (55.56 %) | Healthcare professional : 2/4 (50.0 %) Non healthcare professional : 2/4 (50.0 %) |
| **Indications** | COVID-19 vaccination : 5/15 (33.3 %) COVID-19 immunisation : 5/15 (33.3 %) Unknown : 3/15 (20.0 %) COVID-19 prophylaxis : 3/15 (20.0 %) Product used for unknown indication : 1/15 (6.7 %) | Unknown : 5/9 (55.6 %) Paralysis agitans : 2/9 (22.2 %) Hyperprolactinaemia : 1/9 (11.1 %) Prolactinoma : 1/9 (11.1 %) | Lupus nephritis : 2/4 (50.0 %) Product used for unknown indication : 1/4 (25.0 %) Unknown : 1/4 (25.0 %) Nephrotic syndrome : 1/4 (25.0 %) Proteinuria : 1/4 (25.0 %) |
| **Mean Time to Onset (std)** | 9 days (6 days) | 101 days (193 days) | nan |

|  | **Acebutolol** | **Modafinil** | **Risedronic acid** |
| --- | --- | --- | --- |
| **Number of cases** | 5 | 8 | 14 |
| **IC025_RP_main** | 1.27 | 1.53 | 1.58 |
| **IC025_RP_comparator** | -1.03 | -1.96 | 0.38 |
| **IC025_RP_healthcare** | 0.83 | 0.99 | 1.57 |
| **IC025_RP_malefemale** | 1.42 | 1.34 | 1.13 |
| **IC025_RP-Broad_main** | 4.09 | 3.72 | 1.65 |
| **Sex (Female / Male / Unknown)** | 2 / 3 / 0 | 8 / 0 / 0 | 13 / 1 / 0 |
| **Mean age (SD) in years** | 61.6 (8.38) years | 29.33 (9.67) years | 61.0 (17.83) years |
| **Mean number of suspect/interacting drugs (SD)** | 2.2 (0.84) | 1.71 (1.25) | 4.38 (3.4) |
| **Withdrawn** | 2/5 (40.0 %) | 0/8 (0.0 %) | 1/14 (7.14 %) |
| **Outcome after drug withdrawal** | Recovered: 2/2 (100.0 %) |  | Recovered: 1/1 (100.0 %) |
| **Country of primary source** | France: 4/5 (80.0 %) Netherlands: 1/5 (20.0 %) | United States of America: 7/8 (87.5 %) France: 1/8 (12.5 %) | United States of America: 10/14 (71.4 %) Germany: 1/14 (7.1 %) United Kingdom of Great Britain and Northern Ireland: 1/14 (7.1 %) Japan: 1/14 (7.1 %) France: 1/14 (7.1 %) |
| **Co-reported drugs** | Carteolol hydrochloride: 1/ 5 (20.0 %) Cisplatin: 1/ 5 (20.0 %) Gemcitabine hydrochloride: 1/ 5 (20.0 %) Digoxin: 1/ 5 (20.0 %) Phenprocoumon: 1/ 5 (20.0 %) | Methylphenidate: 4/ 8 (50.0 %) Oxybate sodium: 2/ 8 (25.0 %) Lisinopril: 1/ 8 (12.5 %) Venlafaxine hydrochloride: 1/ 8 (12.5 %) Amfetamine aspartate;Amfetamine sulfate;Dexamfetamine saccharate;Dexamfetamine sulfate: 1/ 8 (12.5 %) | Alendronate sodium: 10/ 14 (71.4 %) Prednisone: 3/ 14 (21.4 %) Hydroxychloroquine: 2/ 14 (14.3 %) Calcium: 2/ 14 (14.3 %) Folic acid: 2/ 14 (14.3 %) |
| **Co-reported reactions** | Skin necrosis: 1/5 (20.0 %) Blood pressure increased: 1/5 (20.0 %) | Drug ineffective: 3/8 (37.5 %) Drug ineffective for unapproved indication: 1/8 (12.5 %) General physical health deterioration: 1/8 (12.5 %) Mouth ulceration: 1/8 (12.5 %) Genital ulceration: 1/8 (12.5 %) | Arthralgia: 7/14 (50.0 %) Anxiety: 6/14 (42.9 %) Nausea: 6/14 (42.9 %) Headache: 6/14 (42.9 %) Fall: 5/14 (35.7 %) |
| **Reporter qualification** | Healthcare professional : 4/5 (80.0 %) Non healthcare professional : 1/5 (20.0 %) | Healthcare professional : 5/8 (62.5 %) Non healthcare professional : 3/8 (37.5 %) | Healthcare professional : 11/14 (78.57 %) Non healthcare professional : 3/14 (21.43 %) |
| **Indications** | Unknown : 2/5 (40.0 %) Essential hypertension, unspecified : 2/5 (40.0 %) Arrhythmia NOS : 1/5 (20.0 %) | Narcolepsy : 5/8 (62.5 %) Unknown : 2/8 (25.0 %) Product used for unknown indication : 1/8 (12.5 %) | Osteoporosis : 9/14 (64.3 %) Unknown : 4/14 (28.6 %) Product used for unknown indication : 2/14 (14.3 %) Osteopenia : 2/14 (14.3 %) Bone density decreased : 1/14 (7.1 %) |
| **Mean Time to Onset (std)** | 347 days (544 days) | nan | 676 days (809 days) |

|  | **Rofecoxib** | **Teriflunomide** | **Tolvaptan** |
| --- | --- | --- | --- |
| **Number of cases** | 39 | 33 | 12 |
| **IC025_RP_main** | 1.54 | 1.56 | 1.56 |
| **IC025_RP_comparator** | 2.23 | 1.16 | -0.23 |
| **IC025_RP_healthcare** | 2.35 | 1.84 | 1.52 |
| **IC025_RP_malefemale** | 2.17 | 1.57 | 0.73 |
| **IC025_RP-Broad_main** | 1.51 | 0.75 | 0.64 |
| **Sex (Female / Male / Unknown)** | 28 / 11 / 0 | 28 / 5 / 0 | 10 / 2 / 0 |
| **Mean age (SD) in years** | 57.82 (11.17) years | 46.93 (13.73) years | 42.33 (9.31) years |
| **Mean number of suspect/interacting drugs (SD)** | 7.31 (5.52) | 3.95 (3.61) | 5.43 (8.46) |
| **Withdrawn** | 14/39 (35.9 %) | 11/33 (33.33 %) | 0/12 (0.0 %) |
| **Outcome after drug withdrawal** | Not recovered: 2/14 (14.29 %) Evolution Unknown: 5/14 (35.71 %) | Recovered: 3/11 (27.27 %) Recovering: 2/11 (18.18 %) Not recovered: 1/11 (9.09 %) Died: 1/11 (9.09 %) Evolution Unknown: 3/11 (27.27 %) |  |
| **Country of primary source** | United States of America: 32/39 (82.1 %) United Kingdom of Great Britain and Northern Ireland: 3/39 (7.7 %) Germany: 2/39 (5.1 %) Canada: 2/39 (5.1 %) | United States of America: 13/33 (39.4 %) Spain: 4/33 (12.1 %) France: 4/33 (12.1 %) Germany: 4/33 (12.1 %) United Kingdom of Great Britain and Northern Ireland: 3/33 (9.1 %) | France: 4/12 (33.3 %) Spain: 3/12 (25.0 %) United States of America: 1/12 (8.3 %) Netherlands: 1/12 (8.3 %) Sweden: 1/12 (8.3 %) |
| **Co-reported drugs** | Prednisone: 10/ 39 (25.6 %) Acetylsalicylic acid: 8/ 39 (20.5 %) Hydrocodone bitartrate;Paracetamol: 6/ 39 (15.4 %) Levothyroxine sodium: 6/ 39 (15.4 %) Nifedipine: 6/ 39 (15.4 %) | Levothyroxine sodium: 4/ 33 (12.1 %) Methylprednisolone: 3/ 33 (9.1 %) Gabapentin: 3/ 33 (9.1 %) Losartan potassium: 2/ 33 (6.1 %) Zolpidem tartrate: 2/ 33 (6.1 %) | Ferrimannitol ovalbumin: 2/ 12 (16.7 %) Iron: 2/ 12 (16.7 %) Levothyroxine sodium: 2/ 12 (16.7 %) Levothyroxine: 2/ 12 (16.7 %) Atorvastatin calcium: 2/ 12 (16.7 %) |
| **Co-reported reactions** | Myocardial infarction: 16/39 (41.0 %) Chest pain: 11/39 (28.2 %) Hypertension: 11/39 (28.2 %) Coronary artery disease: 10/39 (25.6 %) Pain: 8/39 (20.5 %) | Alopecia: 11/33 (33.3 %) Paraesthesia: 8/33 (24.2 %) Pain in extremity: 6/33 (18.2 %) Diarrhoea: 6/33 (18.2 %) Nausea: 6/33 (18.2 %) | Polyuria: 2/12 (16.7 %) Fatigue: 2/12 (16.7 %) Dry mouth: 2/12 (16.7 %) Systemic scleroderma: 1/12 (8.3 %) Influenza: 1/12 (8.3 %) |
| **Reporter qualification** | Healthcare professional : 32/39 (82.05 %) Non healthcare professional : 7/39 (17.95 %) | Healthcare professional : 22/33 (66.67 %) Non healthcare professional : 11/33 (33.33 %) | Healthcare professional : 11/12 (91.67 %) Non healthcare professional : 1/12 (8.33 %) |
| **Indications** | Unknown : 17/39 (43.6 %) Rheumatoid arthritis : 10/39 (25.6 %) Osteoarthritis : 9/39 (23.1 %) Arthritis : 7/39 (17.9 %) Pain : 5/39 (12.8 %) | Multiple sclerosis : 25/33 (75.8 %) Unknown : 6/33 (18.2 %) Relapsing-remitting multiple sclerosis : 4/33 (12.1 %) MS : 1/33 (3.0 %) | Polycystic kidney, autosomal dominant : 5/12 (41.7 %) Unknown : 3/12 (25.0 %) Polycystic kidney : 3/12 (25.0 %) Product used for unknown indication : 2/12 (16.7 %) Congenital cystic kidney disease : 1/12 (8.3 %) |
| **Mean Time to Onset (std)** | 576 days (526 days) | 151 days (134 days) | 412 days (495 days) |

|  | **Topiramate** | **Avapritinib** | **Eptinezumab** |
| --- | --- | --- | --- |
| **Number of cases** | 23 | 8 | 7 |
| **IC025_RP_main** | 1.48 | 1.37 | 1.35 |
| **IC025_RP_comparator** | 0.09 | 1.34 | -0.55 |
| **IC025_RP_healthcare** | 1.02 | -10.35 | 2.08 |
| **IC025_RP_malefemale** | 1.35 | 1.81 | 0.22 |
| **IC025_RP-Broad_main** | 2.21 | 0.42 | 0.46 |
| **Sex (Female / Male / Unknown)** | 21 / 2 / 0 | 8 / 0 / 0 | 5 / 1 / 1 |
| **Mean age (SD) in years** | 40.89 (18.31) years | 68.14 (11.54) years | 53.5 (17.21) years |
| **Mean number of suspect/interacting drugs (SD)** | 3.41 (3.41) | 1.5 (0.71) | 1.33 (0.58) |
| **Withdrawn** | 4/23 (17.39 %) | 0/8 (0.0 %) | 2/7 (28.57 %) |
| **Outcome after drug withdrawal** | Recovering: 1/4 (25.0 %) Evolution Unknown: 2/4 (50.0 %) |  | Recovering: 1/2 (50.0 %) Not recovered: 1/2 (50.0 %) |
| **Country of primary source** | United States of America: 11/23 (47.8 %) United Kingdom of Great Britain and Northern Ireland: 4/23 (17.4 %) France: 3/23 (13.0 %) Netherlands: 3/23 (13.0 %) Belgium: 1/23 (4.3 %) | United States of America: 8/8 (100.0 %) | France: 2/7 (28.6 %) Denmark: 2/7 (28.6 %) Switzerland: 1/7 (14.3 %) Ireland: 1/7 (14.3 %) United States of America: 1/7 (14.3 %) |
| **Co-reported drugs** | Paracetamol: 2/ 23 (8.7 %) Magnesium: 2/ 23 (8.7 %) Levothyroxine: 2/ 23 (8.7 %) Levothyroxine sodium: 2/ 23 (8.7 %) Carbamazepine: 2/ 23 (8.7 %) | Probiotics nos: 1/ 8 (12.5 %) Benzodiazepine derivatives: 1/ 8 (12.5 %) | Omeprazole: 1/ 7 (14.3 %) Bupropion hydrochloride: 1/ 7 (14.3 %) Diphenhydramine hydrochloride: 1/ 7 (14.3 %) Oxetorone fumarate: 1/ 7 (14.3 %) |
| **Co-reported reactions** | Anxiety: 4/23 (17.4 %) Dizziness: 3/23 (13.0 %) Disturbance in attention: 3/23 (13.0 %) Drug ineffective: 3/23 (13.0 %) Depression: 3/23 (13.0 %) | Off label use: 5/8 (62.5 %) Back pain: 4/8 (50.0 %) Feeling abnormal: 4/8 (50.0 %) Drug ineffective: 4/8 (50.0 %) Neoplasm: 4/8 (50.0 %) | Peripheral coldness: 2/7 (28.6 %) Abdominal pain: 1/7 (14.3 %) Pallor: 1/7 (14.3 %) Paraesthesia: 1/7 (14.3 %) |
| **Reporter qualification** | Healthcare professional : 14/23 (60.87 %) Non healthcare professional : 9/23 (39.13 %) | Healthcare professional : 0/8 (0.0 %) Non healthcare professional : 8/8 (100.0 %) | Healthcare professional : 7/7 (100.0 %) Non healthcare professional : 0/7 (0.0 %) |
| **Indications** | Migraine : 9/23 (39.1 %) Unknown : 6/23 (26.1 %) Epilepsy : 3/23 (13.0 %) Migraine prophylaxis : 2/23 (8.7 %) Prophylaxis : 1/23 (4.3 %) | Gastrointestinal stromal tumour : 6/8 (75.0 %) Unknown : 4/8 (50.0 %) Systemic mastocytosis : 1/8 (12.5 %) Indolent systemic mastocytosis : 1/8 (12.5 %) Advanced systemic mastocytosis : 1/8 (12.5 %) | Migraine : 3/7 (42.9 %) Migraine prophylaxis : 2/7 (28.6 %) Product used for unknown indication : 1/7 (14.3 %) Drug use for unknown indication : 1/7 (14.3 %) |
| **Mean Time to Onset (std)** | 281 days (283 days) | nan | 79 days (107 days) |

|  | **Ubrogepant** | **Interferon beta-1a** | **Hydrochlorothiazide;Nebivolol** |
| --- | --- | --- | --- |
| **Number of cases** | 5 | 102 | 4 |
| **IC025_RP_main** | 1.29 | 1.39 | 1.27 |
| **IC025_RP_comparator** | -0.1 | 0.22 | -0.03 |
| **IC025_RP_healthcare** | 0.59 | 2.06 | 1.27 |
| **IC025_RP_malefemale** | -0.48 | 0.98 | -0.39 |
| **IC025_RP-Broad_main** | 1.69 | 0.59 | 1.58 |
| **Sex (Female / Male / Unknown)** | 3 / 2 / 0 | 92 / 10 / 0 | 3 / 1 / 0 |
| **Mean age (SD) in years** | 53.0 (0.0) years | 46.03 (12.09) years | 64.0 (7.07) years |
| **Mean number of suspect/interacting drugs (SD)** | 6.0 (5.96) | 5.0 (4.93) | 6.67 (8.08) |
| **Withdrawn** | 1/5 (20.0 %) | 30/102 (29.41 %) | 4/4 (100.0 %) |
| **Outcome after drug withdrawal** | Not recovered: 1/1 (100.0 %) | Recovered: 4/30 (13.33 %) Recovering: 2/30 (6.67 %) Not recovered: 10/30 (33.33 %) Evolution Unknown: 11/30 (36.67 %) | Recovered: 2/4 (50.0 %) Recovering: 1/4 (25.0 %) Evolution Unknown: 1/4 (25.0 %) |
| **Country of primary source** | United States of America: 5/5 (100.0 %) | United States of America: 63/102 (61.8 %) Germany: 8/102 (7.8 %) United Kingdom of Great Britain and Northern Ireland: 7/102 (6.9 %) Spain: 4/102 (3.9 %) France: 4/102 (3.9 %) | France: 3/4 (75.0 %) Portugal: 1/4 (25.0 %) |
| **Co-reported drugs** | Botulinum toxin type a: 3/ 5 (60.0 %) Linaclotide: 2/ 5 (40.0 %) Atogepant: 2/ 5 (40.0 %) Nortriptyline: 2/ 5 (40.0 %) Salbutamol: 2/ 5 (40.0 %) | Gabapentin: 7/ 102 (6.9 %) Levothyroxine sodium: 7/ 102 (6.9 %) Levothyroxine: 7/ 102 (6.9 %) Fampridine: 6/ 102 (5.9 %) Baclofen: 5/ 102 (4.9 %) | Nebivolol hydrochloride: 3/ 4 (75.0 %) Pyridoxine: 2/ 4 (50.0 %) Pyridoxine hydrochloride: 2/ 4 (50.0 %) Furosemide: 1/ 4 (25.0 %) Potassium chloride: 1/ 4 (25.0 %) |
| **Co-reported reactions** | Pain: 1/5 (20.0 %) Dementia: 1/5 (20.0 %) Muscle spasms: 1/5 (20.0 %) Restless legs syndrome: 1/5 (20.0 %) Arthritis: 1/5 (20.0 %) | Influenza like illness: 11/102 (10.8 %) Fatigue: 10/102 (9.8 %) Multiple sclerosis: 7/102 (6.9 %) Multiple sclerosis relapse: 7/102 (6.9 %) Injection site erythema: 6/102 (5.9 %) | Hypokalaemia: 1/4 (25.0 %) Hypoaesthesia: 1/4 (25.0 %) Paraesthesia: 1/4 (25.0 %) Hyponatraemia: 1/4 (25.0 %) Clubbing: 1/4 (25.0 %) |
| **Reporter qualification** | Healthcare professional : 3/5 (60.0 %) Non healthcare professional : 2/5 (40.0 %) | Healthcare professional : 52/102 (50.98 %) Non healthcare professional : 50/102 (49.02 %) | Healthcare professional : 4/4 (100.0 %) Non healthcare professional : 0/4 (0.0 %) |
| **Indications** | Migraine : 4/5 (80.0 %) Unknown : 1/5 (20.0 %) Migraine without aura : 1/5 (20.0 %) Headache : 1/5 (20.0 %) | Multiple sclerosis : 76/102 (74.5 %) Unknown : 22/102 (21.6 %) Product used for unknown indication : 8/102 (7.8 %) Relapsing-remitting multiple sclerosis : 4/102 (3.9 %) Drug use for unknown indication : 3/102 (2.9 %) | Hypertension arterial : 3/4 (75.0 %) Unknown : 1/4 (25.0 %) |
| **Mean Time to Onset (std)** | nan | 1449 days (1327 days) | 75 days (94 days) |

|  | **Dorzolamide;Timolol** | **Interferon beta** | **Peginterferon alfa-2b** |
| --- | --- | --- | --- |
| **Number of cases** | 8 | 4 | 15 |
| **IC025_RP_main** | 1.22 | 1.1 | 1.22 |
| **IC025_RP_comparator** | -0.12 | 1.08 | 0.15 |
| **IC025_RP_healthcare** | 1.55 | 0.45 | 0.88 |
| **IC025_RP_malefemale** | 1.44 | 1.13 | 1.14 |
| **IC025_RP-Broad_main** | 0.47 | 1.2 | 0.4 |
| **Sex (Female / Male / Unknown)** | 5 / 3 / 0 | 2 / 2 / 0 | 9 / 6 / 0 |
| **Mean age (SD) in years** | 65.5 (18.84) years | 59.0 (5.1) years | 49.18 (7.31) years |
| **Mean number of suspect/interacting drugs (SD)** | 3.5 (3.11) | 1.0 (0.0) | 5.33 (7.75) |
| **Withdrawn** | 4/8 (50.0 %) | 0/4 (0.0 %) | 3/15 (20.0 %) |
| **Outcome after drug withdrawal** | Recovered: 3/4 (75.0 %) Recovering: 1/4 (25.0 %) |  | Recovering: 1/3 (33.33 %) Not recovered: 2/3 (66.67 %) |
| **Country of primary source** | Spain: 2/8 (25.0 %) United Kingdom of Great Britain and Northern Ireland: 1/8 (12.5 %) France: 1/8 (12.5 %) United States of America: 1/8 (12.5 %) Belgium: 1/8 (12.5 %) | Switzerland: 3/4 (75.0 %) Italy: 1/4 (25.0 %) | United States of America: 9/15 (60.0 %) Norway: 1/15 (6.7 %) Italy: 1/15 (6.7 %) Germany: 1/15 (6.7 %) Spain: 1/15 (6.7 %) |
| **Co-reported drugs** | Brimonidine tartrate: 2/ 8 (25.0 %) Atorvastatin: 1/ 8 (12.5 %) Simvastatin: 1/ 8 (12.5 %) Acetylsalicylic acid: 1/ 8 (12.5 %) Sodium picosulfate: 1/ 8 (12.5 %) | Corticosteroids: 1/ 4 (25.0 %) Methylprednisolone acetate: 1/ 4 (25.0 %) | Ribavirin: 12/ 15 (80.0 %) Amitriptyline hydrochloride: 2/ 15 (13.3 %) Prednisone: 2/ 15 (13.3 %) Venlafaxine hydrochloride: 2/ 15 (13.3 %) Nortriptyline hydrochloride: 1/ 15 (6.7 %) |
| **Co-reported reactions** | Paraesthesia: 2/8 (25.0 %) Lymphoedema: 1/8 (12.5 %) Drug ineffective: 1/8 (12.5 %) Arthralgia: 1/8 (12.5 %) Musculoskeletal stiffness: 1/8 (12.5 %) | Skin disorder: 3/4 (75.0 %) Skin ulcer: 2/4 (50.0 %) Oedema: 1/4 (25.0 %) Gastric cancer: 1/4 (25.0 %) Arthralgia: 1/4 (25.0 %) | Pyrexia: 5/15 (33.3 %) Alopecia: 4/15 (26.7 %) Fatigue: 3/15 (20.0 %) Viral load increased: 3/15 (20.0 %) Nausea: 3/15 (20.0 %) |
| **Reporter qualification** | Healthcare professional : 7/8 (87.5 %) Non healthcare professional : 1/8 (12.5 %) | Healthcare professional : 3/4 (75.0 %) Non healthcare professional : 1/4 (25.0 %) | Healthcare professional : 11/15 (73.33 %) Non healthcare professional : 4/15 (26.67 %) |
| **Indications** | Unknown : 3/8 (37.5 %) Ocular hypertension : 2/8 (25.0 %) Glaucoma : 1/8 (12.5 %) Glaucoma (excl congenital) : 1/8 (12.5 %) Glaucoma simplex : 1/8 (12.5 %) | Multiple sclerosis : 2/4 (50.0 %) Unknown : 1/4 (25.0 %) Relapsing-remitting multiple sclerosis : 1/4 (25.0 %) | Hepatitis C : 7/15 (46.7 %) Chronic hepatitis C : 3/15 (20.0 %) Unknown : 3/15 (20.0 %) Malignant melanoma : 1/15 (6.7 %) Drug use for unknown indication : 1/15 (6.7 %) |
| **Mean Time to Onset (std)** | 1516 days (1180 days) | nan | 188 days (115 days) |

|  | **Timolol** | **Ethinylestradiol;Levonorgestrel** | **Carvedilol** |
| --- | --- | --- | --- |
| **Number of cases** | 11 | 18 | 15 |
| **IC025_RP_main** | 1.19 | 1.18 | 1.55 |
| **IC025_RP_comparator** | -1.12 | 1.0 | -1.74 |
| **IC025_RP_healthcare** | 0.44 | 0.94 | 0.64 |
| **IC025_RP_malefemale** | 1.77 | 0.69 | 1.02 |
| **IC025_RP-Broad_main** | 3.04 | 2.33 | 3.54 |
| **Sex (Female / Male / Unknown)** | 9 / 2 / 0 | 17 / 1 / 0 | 6 / 6 / 3 |
| **Mean age (SD) in years** | 64.11 (10.29) years | 27.68 (12.47) years | 58.0 (16.43) years |
| **Mean number of suspect/interacting drugs (SD)** | 4.4 (4.51) | 3.0 (2.0) | 2.5 (1.22) |
| **Withdrawn** | 8/11 (72.73 %) | 8/18 (44.44 %) | 2/15 (13.33 %) |
| **Outcome after drug withdrawal** | Recovered: 5/8 (62.5 %) Not recovered: 1/8 (12.5 %) Evolution Unknown: 2/8 (25.0 %) | Recovered: 3/8 (37.5 %) Recovering: 1/8 (12.5 %) Not recovered: 1/8 (12.5 %) Evolution Unknown: 2/8 (25.0 %) | Recovered: 1/2 (50.0 %) Recovering: 1/2 (50.0 %) |
| **Country of primary source** | France: 3/11 (27.3 %) Germany: 2/11 (18.2 %) United States of America: 1/11 (9.1 %) Switzerland: 1/11 (9.1 %) Netherlands: 1/11 (9.1 %) | United Kingdom of Great Britain and Northern Ireland: 7/18 (38.9 %) France: 4/18 (22.2 %) United States of America: 2/18 (11.1 %) Germany: 2/18 (11.1 %) Canada: 1/18 (5.6 %) | United States of America: 8/15 (53.3 %) Italy: 2/15 (13.3 %) Spain: 1/15 (6.7 %) Sweden: 1/15 (6.7 %) Canada: 1/15 (6.7 %) |
| **Co-reported drugs** | Acetylsalicylic acid: 2/ 11 (18.2 %) Fexofenadine hydrochloride;Pseudoephedrine hydrochloride: 1/ 11 (9.1 %) Netarsudil mesilate: 1/ 11 (9.1 %) Betaxolol hydrochloride: 1/ 11 (9.1 %) Simvastatin: 1/ 11 (9.1 %) | Propranolol: 2/ 18 (11.1 %) Propranolol hydrochloride: 2/ 18 (11.1 %) Valproate sodium: 1/ 18 (5.6 %) Clozapine: 1/ 18 (5.6 %) Interferon beta-1b: 1/ 18 (5.6 %) | Tozinameran: 2/ 15 (13.3 %) Hydrochlorothiazide;Lisinopril: 2/ 15 (13.3 %) Doxazosin: 2/ 15 (13.3 %) Sumatriptan succinate: 1/ 15 (6.7 %) Prednisone: 1/ 15 (6.7 %) |
| **Co-reported reactions** | Dyspnoea: 2/11 (18.2 %) Eye irritation: 2/11 (18.2 %) Peripheral coldness: 2/11 (18.2 %) Muscular weakness: 1/11 (9.1 %) Chest discomfort: 1/11 (9.1 %) | Skin discolouration: 3/18 (16.7 %) Hypoaesthesia: 2/18 (11.1 %) Thrombosis: 2/18 (11.1 %) Pain in extremity: 2/18 (11.1 %) Skin hypertrophy: 1/18 (5.6 %) | Condition aggravated: 2/15 (13.3 %) Drug hypersensitivity: 1/15 (6.7 %) Sedation: 1/15 (6.7 %) Dry eye: 1/15 (6.7 %) Muscle spasms: 1/15 (6.7 %) |
| **Reporter qualification** | Healthcare professional : 5/11 (45.45 %) Non healthcare professional : 6/11 (54.55 %) | Healthcare professional : 10/18 (55.56 %) Non healthcare professional : 8/18 (44.44 %) | Healthcare professional : 7/15 (46.67 %) Non healthcare professional : 8/15 (53.33 %) |
| **Indications** | Glaucoma : 5/11 (45.5 %) Unknown : 2/11 (18.2 %) Product used for unknown indication : 1/11 (9.1 %) Ocular hypertension : 1/11 (9.1 %) Hypertension ocular : 1/11 (9.1 %) | Unknown : 9/18 (50.0 %) Contraception : 5/18 (27.8 %) Oral contraception : 2/18 (11.1 %) Birth control : 1/18 (5.6 %) Drug use for unknown indication : 1/18 (5.6 %) | Unknown : 4/15 (26.7 %) Drug use for unknown indication : 3/15 (20.0 %) Hypertension : 3/15 (20.0 %) Hypertension arterial : 2/15 (13.3 %) Product used for unknown indication : 1/15 (6.7 %) |
| **Mean Time to Onset (std)** | 128 days (155 days) | 1560 days (2329 days) | 6 days (7 days) |

|  | **Melatonin** | **Benfluorex** | **Asfotase alfa** |
| --- | --- | --- | --- |
| **Number of cases** | 4 | 6 | 7 |
| **IC025_RP_main** | 0.19 | 1.05 | 1.04 |
| **IC025_RP_comparator** | 0.49 | 1.51 | 1.16 |
| **IC025_RP_healthcare** | -1.27 | 1.1 | -2.78 |
| **IC025_RP_malefemale** | -1.36 | -1.18 | -2.76 |
| **IC025_RP-Broad_main** | 4.13 | 0.18 | 0.08 |
| **Sex (Female / Male / Unknown)** | 1 / 3 / 0 | 4 / 2 / 0 | 7 / 0 / 0 |
| **Mean age (SD) in years** | 9.0 (0.0) years | 46.5 (23.33) years | 55.0 (nan) years |
| **Mean number of suspect/interacting drugs (SD)** | 2.67 (1.53) | 1.0 (nan) | 7.0 (4.24) |
| **Withdrawn** | 1/4 (25.0 %) | 1/6 (16.67 %) | 0/7 (0.0 %) |
| **Outcome after drug withdrawal** | Recovered: 1/1 (100.0 %) | Not recovered: 1/1 (100.0 %) |  |
| **Country of primary source** | Australia: 1/4 (25.0 %) France: 1/4 (25.0 %) Netherlands: 1/4 (25.0 %) United Kingdom of Great Britain and Northern Ireland: 1/4 (25.0 %) | France: 6/6 (100.0 %) | United States of America: 7/7 (100.0 %) |
| **Co-reported drugs** | Methylphenidate hydrochloride: 4/ 4 (100.0 %) Methylphenidate: 4/ 4 (100.0 %) Budesonide;Formoterol fumarate: 1/ 4 (25.0 %) Bilastine: 1/ 4 (25.0 %) Salbutamol: 1/ 4 (25.0 %) | Glipizide: 1/ 6 (16.7 %) | Ibuprofen: 2/ 7 (28.6 %) Paracetamol: 1/ 7 (14.3 %) Acetylsalicylic acid: 1/ 7 (14.3 %) Gabapentin: 1/ 7 (14.3 %) Thyroid: 1/ 7 (14.3 %) |
| **Co-reported reactions** | Autoimmune disorder: 1/4 (25.0 %) Rash: 1/4 (25.0 %) Peripheral swelling: 1/4 (25.0 %) Amaurosis: 1/4 (25.0 %) Paraesthesia: 1/4 (25.0 %) | Aortic valve incompetence: 4/6 (66.7 %) Mitral valve incompetence: 4/6 (66.7 %) Aortic valve thickening: 4/6 (66.7 %) Mitral valve stenosis: 2/6 (33.3 %) Hypertension: 2/6 (33.3 %) | Neuropathy peripheral: 3/7 (42.9 %) Fatigue: 3/7 (42.9 %) Peripheral swelling: 2/7 (28.6 %) Condition aggravated: 2/7 (28.6 %) Headache: 2/7 (28.6 %) |
| **Reporter qualification** | Healthcare professional : 2/4 (50.0 %) Non healthcare professional : 2/4 (50.0 %) | Healthcare professional : 6/6 (100.0 %) Non healthcare professional : 0/6 (0.0 %) | Healthcare professional : 1/7 (14.29 %) Non healthcare professional : 6/7 (85.71 %) |
| **Indications** | Drug use for unknown indication : 2/4 (50.0 %) Sleep disorder : 1/4 (25.0 %) Sleep problem : 1/4 (25.0 %) | Unknown : 2/6 (33.3 %) Product used for unknown indication : 1/6 (16.7 %) Overweight : 1/6 (16.7 %) Diabetes : 1/6 (16.7 %) Hypercholesterolemia : 1/6 (16.7 %) | Product used for unknown indication : 4/7 (57.1 %) Hypophosphatasia : 3/7 (42.9 %) Unknown : 2/7 (28.6 %) |
| **Mean Time to Onset (std)** | 163 days (281 days) | 1988 days (1346 days) | nan |

|  | **Clonidine** | **Agalsidase alfa** | **Atogepant** |
| --- | --- | --- | --- |
| **Number of cases** | 12 | 5 | 5 |
| **IC025_RP_main** | 1.18 | 0.98 | 0.95 |
| **IC025_RP_comparator** | -0.26 | 1.05 | -0.76 |
| **IC025_RP_healthcare** | 0.76 | 0.99 | 1.67 |
| **IC025_RP_malefemale** | 1.28 | -0.95 | -10.42 |
| **IC025_RP-Broad_main** | 3.57 | 1.21 | 0.9 |
| **Sex (Female / Male / Unknown)** | 6 / 6 / 0 | 1 / 4 / 0 | 3 / 1 / 1 |
| **Mean age (SD) in years** | 48.6 (21.7) years | 68.0 (14.14) years | nan (nan) years |
| **Mean number of suspect/interacting drugs (SD)** | 2.33 (2.34) | 13.0 (7.35) | 6.0 (nan) |
| **Withdrawn** | 5/12 (41.67 %) | 1/5 (20.0 %) | 3/5 (60.0 %) |
| **Outcome after drug withdrawal** | Recovered: 2/5 (40.0 %) Recovering: 1/5 (20.0 %) Not recovered: 2/5 (40.0 %) | Evolution Unknown: 1/1 (100.0 %) | Recovering: 2/3 (66.67 %) Evolution Unknown: 1/3 (33.33 %) |
| **Country of primary source** | United States of America: 3/12 (25.0 %) Netherlands: 2/12 (16.7 %) United Kingdom of Great Britain and Northern Ireland: 2/12 (16.7 %) France: 2/12 (16.7 %) New Zealand: 1/12 (8.3 %) | Germany: 5/5 (100.0 %) | United States of America: 4/5 (80.0 %) United Kingdom of Great Britain and Northern Ireland: 1/5 (20.0 %) |
| **Co-reported drugs** | Methylphenidate hydrochloride: 1/ 12 (8.3 %) Lisdexamfetamine mesilate: 1/ 12 (8.3 %) Acetylsalicylic acid;Butalbital;Caffeine;Codeine phosphate;Phenacetin: 1/ 12 (8.3 %) Enalapril maleate: 1/ 12 (8.3 %) Hydrochlorothiazide: 1/ 12 (8.3 %) | Paracetamol: 5/ 5 (100.0 %) Metamizole sodium: 4/ 5 (80.0 %) Metamizole: 4/ 5 (80.0 %) Ramipril: 3/ 5 (60.0 %) Acetylsalicylic acid: 3/ 5 (60.0 %) | Botulinum toxin type a: 1/ 5 (20.0 %) Ubrogepant: 1/ 5 (20.0 %) Atorvastatin: 1/ 5 (20.0 %) Thioctic acid: 1/ 5 (20.0 %) Linaclotide: 1/ 5 (20.0 %) |
| **Co-reported reactions** | Scleroderma: 1/12 (8.3 %) Hallucination, visual: 1/12 (8.3 %) Palpitations: 1/12 (8.3 %) Pollakiuria: 1/12 (8.3 %) Therapeutic product effect decreased: 1/12 (8.3 %) | Angiokeratoma: 3/5 (60.0 %) Tinnitus: 3/5 (60.0 %) Temperature intolerance: 2/5 (40.0 %) Dyspnoea: 2/5 (40.0 %) Palpitations: 2/5 (40.0 %) | Surgery: 1/5 (20.0 %) Pain: 1/5 (20.0 %) Peripheral coldness: 1/5 (20.0 %) Skin discolouration: 1/5 (20.0 %) Pain in extremity: 1/5 (20.0 %) |
| **Reporter qualification** | Healthcare professional : 7/12 (58.33 %) Non healthcare professional : 5/12 (41.67 %) | Healthcare professional : 5/5 (100.0 %) Non healthcare professional : 0/5 (0.0 %) | Healthcare professional : 5/5 (100.0 %) Non healthcare professional : 0/5 (0.0 %) |
| **Indications** | Hypertension : 3/12 (25.0 %) Unknown : 2/12 (16.7 %) ADHD : 1/12 (8.3 %) Post-traumatic stress disorder : 1/12 (8.3 %) Product used for unknown indication : 1/12 (8.3 %) | Fabry's disease : 5/5 (100.0 %) | Migraine : 3/5 (60.0 %) Unknown : 1/5 (20.0 %) Product used for unknown indication : 1/5 (20.0 %) |
| **Mean Time to Onset (std)** | 378 days (723 days) | 2355 days (946 days) | nan |

|  | **Ethinylestradiol;Etonogestrel** | **Latanoprost;Timolol** | **Paricalcitol** |
| --- | --- | --- | --- |
| **Number of cases** | 16 | 4 | 5 |
| **IC025_RP_main** | 1.02 | 0.89 | 0.93 |
| **IC025_RP_comparator** | 1.26 | 0.12 | 0.2 |
| **IC025_RP_healthcare** | 0.07 | -2.55 | -0.96 |
| **IC025_RP_malefemale** | -0.39 | 1.09 | -2.74 |
| **IC025_RP-Broad_main** | 0.78 | 0.85 | 2.09 |
| **Sex (Female / Male / Unknown)** | 16 / 0 / 0 | 3 / 1 / 0 | 1 / 4 / 0 |
| **Mean age (SD) in years** | 31.67 (5.09) years | 70.5 (1.29) years | 56.0 (nan) years |
| **Mean number of suspect/interacting drugs (SD)** | 3.33 (2.87) | 1.67 (1.15) | nan (nan) |
| **Withdrawn** | 5/16 (31.25 %) | 0/4 (0.0 %) | 0/5 (0.0 %) |
| **Outcome after drug withdrawal** | Evolution Unknown: 4/5 (80.0 %) |  |  |
| **Country of primary source** | United States of America: 15/16 (93.8 %) Belgium: 1/16 (6.2 %) | Germany: 1/4 (25.0 %) France: 1/4 (25.0 %) Canada: 1/4 (25.0 %) United Kingdom of Great Britain and Northern Ireland: 1/4 (25.0 %) | Colombia: 5/5 (100.0 %) |
| **Co-reported drugs** | Salbutamol: 3/ 16 (18.8 %) Salbutamol sulfate: 3/ 16 (18.8 %) Amfetamine aspartate;Amfetamine sulfate;Dexamfetamine saccharate;Dexamfetamine sulfate: 2/ 16 (12.5 %) Ethanol: 1/ 16 (6.2 %) Mometasone furoate: 1/ 16 (6.2 %) | Carteolol hydrochloride: 1/ 4 (25.0 %) Latanoprost: 1/ 4 (25.0 %) Dorzolamide hydrochloride: 1/ 4 (25.0 %) Acetylsalicylic acid: 1/ 4 (25.0 %) Beclometasone dipropionate: 1/ 4 (25.0 %) |  |
| **Co-reported reactions** | Pulmonary embolism: 8/16 (50.0 %) Fatigue: 6/16 (37.5 %) Deep vein thrombosis: 6/16 (37.5 %) Anxiety: 5/16 (31.2 %) Oedema peripheral: 4/16 (25.0 %) | Dyspnoea: 2/4 (50.0 %) Paraesthesia: 1/4 (25.0 %) Peripheral sensory neuropathy: 1/4 (25.0 %) Abnormal behaviour: 1/4 (25.0 %) Aggression: 1/4 (25.0 %) | Localised oedema: 1/5 (20.0 %) |
| **Reporter qualification** | Healthcare professional : 6/16 (37.5 %) Non healthcare professional : 10/16 (62.5 %) | Healthcare professional : 1/4 (25.0 %) Non healthcare professional : 3/4 (75.0 %) | Healthcare professional : 2/5 (40.0 %) Non healthcare professional : 3/5 (60.0 %) |
| **Indications** | Contraception : 11/16 (68.8 %) Unknown : 4/16 (25.0 %) Polycystic ovaries : 2/16 (12.5 %) Menorrhagia : 1/16 (6.2 %) Ovarian cyst : 1/16 (6.2 %) | Unknown : 2/4 (50.0 %) Drug use for unknown indication : 1/4 (25.0 %) Glaucoma : 1/4 (25.0 %) | Primary hyperparathyroidism : 4/5 (80.0 %) Hyperparathyroidism : 1/5 (20.0 %) |
| **Mean Time to Onset (std)** | nan | nan | 585 days (515 days) |

|  | **Tofacitinib** | **Isotretinoin** | **Pergolide** |
| --- | --- | --- | --- |
| **Number of cases** | 52 | 35 | 4 |
| **IC025_RP_main** | 1.0 | 1.03 | 0.85 |
| **IC025_RP_comparator** | 0.58 | 1.69 | 0.26 |
| **IC025_RP_healthcare** | 1.3 | 1.19 | 0.36 |
| **IC025_RP_malefemale** | 0.67 | 0.78 | 1.04 |
| **IC025_RP-Broad_main** | 0.31 | 1.11 | 2.59 |
| **Sex (Female / Male / Unknown)** | 47 / 4 / 1 | 22 / 13 / 0 | 3 / 1 / 0 |
| **Mean age (SD) in years** | 57.05 (14.18) years | 22.92 (6.4) years | 75.25 (4.99) years |
| **Mean number of suspect/interacting drugs (SD)** | 10.33 (9.13) | 2.6 (3.47) | 2.67 (2.08) |
| **Withdrawn** | 21/52 (40.38 %) | 7/35 (20.0 %) | 0/4 (0.0 %) |
| **Outcome after drug withdrawal** | Recovered: 2/21 (9.52 %) Recovering: 3/21 (14.29 %) Not recovered: 2/21 (9.52 %) Evolution Unknown: 10/21 (47.62 %) | Recovered: 1/7 (14.29 %) Not recovered: 3/7 (42.86 %) Evolution Unknown: 3/7 (42.86 %) |  |
| **Country of primary source** | United States of America: 47/52 (90.4 %) Canada: 2/52 (3.8 %) Portugal: 1/52 (1.9 %) France: 1/52 (1.9 %) United Kingdom of Great Britain and Northern Ireland: 1/52 (1.9 %) | United States of America: 18/35 (51.4 %) France: 7/35 (20.0 %) United Kingdom of Great Britain and Northern Ireland: 4/35 (11.4 %) Norway: 2/35 (5.7 %) Spain: 1/35 (2.9 %) | United Kingdom of Great Britain and Northern Ireland: 2/4 (50.0 %) France: 1/4 (25.0 %) Netherlands: 1/4 (25.0 %) |
| **Co-reported drugs** | Hydroxychloroquine: 10/ 52 (19.2 %) Hydroxychloroquine sulfate: 10/ 52 (19.2 %) Methotrexate sodium: 8/ 52 (15.4 %) Methotrexate: 8/ 52 (15.4 %) Prednisone: 7/ 52 (13.5 %) | Ethinylestradiol;Norgestimate: 2/ 35 (5.7 %) Intrauterine contraceptive device: 1/ 35 (2.9 %) Methadone: 1/ 35 (2.9 %) Methylprednisolone acetate: 1/ 35 (2.9 %) Cetirizine hydrochloride: 1/ 35 (2.9 %) | Carbidopa Monohydrate;Levodopa: 2/ 4 (50.0 %) Zopiclone: 1/ 4 (25.0 %) Heptaminol hydrochloride: 1/ 4 (25.0 %) Viloxazine: 1/ 4 (25.0 %) Domperidone: 1/ 4 (25.0 %) |
| **Co-reported reactions** | Arthralgia: 19/52 (36.5 %) Fatigue: 15/52 (28.8 %) Dry mouth: 9/52 (17.3 %) Headache: 9/52 (17.3 %) Musculoskeletal stiffness: 9/52 (17.3 %) | Inflammatory bowel disease: 6/35 (17.1 %) Arthralgia: 6/35 (17.1 %) Colitis: 5/35 (14.3 %) Headache: 5/35 (14.3 %) Crohn's disease: 5/35 (14.3 %) | Pain: 1/4 (25.0 %) |
| **Reporter qualification** | Healthcare professional : 40/52 (76.92 %) Non healthcare professional : 12/52 (23.08 %) | Healthcare professional : 23/35 (65.71 %) Non healthcare professional : 12/35 (34.29 %) | Healthcare professional : 2/4 (50.0 %) Non healthcare professional : 2/4 (50.0 %) |
| **Indications** | Unknown : 31/52 (59.6 %) Rheumatoid arthritis : 19/52 (36.5 %) Product used for unknown indication : 2/52 (3.8 %) Arthritis : 2/52 (3.8 %) Endometrial cancer stage IV : 1/52 (1.9 %) | Acne : 17/35 (48.6 %) Unknown : 17/35 (48.6 %) Acne NOS : 2/35 (5.7 %) Other acne of sebaceous glands : 1/35 (2.9 %) Diseases of sebaceous glands : 1/35 (2.9 %) | Parkinson's disease : 2/4 (50.0 %) Unknown : 1/4 (25.0 %) Other specified extrapyramidal and movement disorders : 1/4 (25.0 %) |
| **Mean Time to Onset (std)** | 122 days (42 days) | 956 days (1504 days) | nan |

|  | **Pemetrexed** | **Hydroxychloroquine** | **Interferon beta-1b** |
| --- | --- | --- | --- |
| **Number of cases** | 17 | 18 | 29 |
| **IC025_RP_main** | 0.94 | 0.84 | 0.92 |
| **IC025_RP_comparator** | 0.38 | 0.45 | -0.22 |
| **IC025_RP_healthcare** | 1.31 | 0.28 | 1.04 |
| **IC025_RP_malefemale** | 1.53 | 0.37 | 0.9 |
| **IC025_RP-Broad_main** | 1.44 | 3.2 | 0.24 |
| **Sex (Female / Male / Unknown)** | 14 / 3 / 0 | 15 / 2 / 1 | 23 / 5 / 1 |
| **Mean age (SD) in years** | 63.29 (4.19) years | 46.0 (14.63) years | 43.69 (8.04) years |
| **Mean number of suspect/interacting drugs (SD)** | 3.64 (6.86) | 5.18 (3.78) | 4.47 (3.7) |
| **Withdrawn** | 5/17 (29.41 %) | 3/18 (16.67 %) | 6/29 (20.69 %) |
| **Outcome after drug withdrawal** | Recovered: 1/5 (20.0 %) Not recovered: 4/5 (80.0 %) | Recovering: 1/3 (33.33 %) Not recovered: 1/3 (33.33 %) Evolution Unknown: 1/3 (33.33 %) | Recovering: 3/6 (50.0 %) Evolution Unknown: 3/6 (50.0 %) |
| **Country of primary source** | United States of America: 10/17 (58.8 %) Australia: 2/17 (11.8 %) Finland: 2/17 (11.8 %) France: 1/17 (5.9 %) United Kingdom of Great Britain and Northern Ireland: 1/17 (5.9 %) | United States of America: 8/18 (44.4 %) Canada: 4/18 (22.2 %) Germany: 1/18 (5.6 %) Paraguay: 1/18 (5.6 %) Finland: 1/18 (5.6 %) | Germany: 14/29 (48.3 %) United States of America: 6/29 (20.7 %) Canada: 5/29 (17.2 %) Spain: 1/29 (3.4 %) United Kingdom of Great Britain and Northern Ireland: 1/29 (3.4 %) |
| **Co-reported drugs** | Carboplatin: 11/ 17 (64.7 %) Pembrolizumab: 3/ 17 (17.6 %) Metoclopramide hydrochloride: 2/ 17 (11.8 %) Dexamethasone: 2/ 17 (11.8 %) Pantoprazole: 2/ 17 (11.8 %) | Prednisone: 7/ 18 (38.9 %) Methotrexate: 5/ 18 (27.8 %) Mycophenolate mofetil: 4/ 18 (22.2 %) Belimumab: 3/ 18 (16.7 %) Rituximab: 3/ 18 (16.7 %) | Amantadine: 3/ 29 (10.3 %) Amantadine hydrochloride: 3/ 29 (10.3 %) Cyclobenzaprine: 3/ 29 (10.3 %) Cyclobenzaprine hydrochloride: 3/ 29 (10.3 %) Paracetamol: 2/ 29 (6.9 %) |
| **Co-reported reactions** | Cyanosis: 2/17 (11.8 %) Gangrene: 2/17 (11.8 %) Skin ulcer: 2/17 (11.8 %) Pain: 2/17 (11.8 %) Anaemia macrocytic: 1/17 (5.9 %) | Headache: 7/18 (38.9 %) Arthralgia: 7/18 (38.9 %) Nausea: 7/18 (38.9 %) Fatigue: 7/18 (38.9 %) Weight decreased: 6/18 (33.3 %) | Skin discolouration: 6/29 (20.7 %) Injection site reaction: 4/29 (13.8 %) Condition aggravated: 3/29 (10.3 %) Peripheral vascular disorder: 3/29 (10.3 %) Fatigue: 3/29 (10.3 %) |
| **Reporter qualification** | Healthcare professional : 17/17 (100.0 %) Non healthcare professional : 0/17 (0.0 %) | Healthcare professional : 12/18 (66.67 %) Non healthcare professional : 6/18 (33.33 %) | Healthcare professional : 20/29 (68.97 %) Non healthcare professional : 9/29 (31.03 %) |
| **Indications** | Lung adenocarcinoma : 10/17 (58.8 %) Lung cancer : 2/17 (11.8 %) Non-small cell lung cancer metastatic : 1/17 (5.9 %) Lung adenocarcinoma stage IV : 1/17 (5.9 %) Non-small cell lung cancer : 1/17 (5.9 %) | Product used for unknown indication : 6/18 (33.3 %) Systemic lupus erythematosus : 3/18 (16.7 %) Connective tissue disorder : 2/18 (11.1 %) Lupus nephritis : 1/18 (5.6 %) Psoriatic arthritis : 1/18 (5.6 %) | Multiple sclerosis : 16/29 (55.2 %) Unknown : 7/29 (24.1 %) Relapsing-remitting multiple sclerosis : 3/29 (10.3 %) MS : 2/29 (6.9 %) Necrosis NOS : 1/29 (3.4 %) |
| **Mean Time to Onset (std)** | 18 days (28 days) | 596 days (417 days) | 1742 days (1944 days) |

|  | **Eletriptan** | **Prednisone** | **Minocycline** |
| --- | --- | --- | --- |
| **Number of cases** | 5 | 30 | 10 |
| **IC025_RP_main** | 0.81 | 0.59 | 0.83 |
| **IC025_RP_comparator** | -0.99 | 1.12 | 1.68 |
| **IC025_RP_healthcare** | 0.12 | 0.37 | -0.72 |
| **IC025_RP_malefemale** | -0.1 | 0.7 | 0.49 |
| **IC025_RP-Broad_main** | 1.77 | 3.13 | 1.26 |
| **Sex (Female / Male / Unknown)** | 5 / 0 / 0 | 26 / 3 / 1 | 6 / 3 / 1 |
| **Mean age (SD) in years** | 48.33 (4.04) years | 45.48 (17.15) years | 25.5 (11.33) years |
| **Mean number of suspect/interacting drugs (SD)** | 4.33 (3.06) | 4.92 (3.75) | 1.5 (1.22) |
| **Withdrawn** | 3/5 (60.0 %) | 0/30 (0.0 %) | 3/10 (30.0 %) |
| **Outcome after drug withdrawal** | Recovered: 2/3 (66.67 %) |  | Recovering: 1/3 (33.33 %) Recovered with sequelae: 1/3 (33.33 %) Evolution Unknown: 1/3 (33.33 %) |
| **Country of primary source** | United States of America: 4/5 (80.0 %) United Kingdom of Great Britain and Northern Ireland: 1/5 (20.0 %) | United States of America: 18/30 (60.0 %) Canada: 6/30 (20.0 %) United Kingdom of Great Britain and Northern Ireland: 2/30 (6.7 %) Spain: 2/30 (6.7 %) Czechia: 1/30 (3.3 %) | United States of America: 3/10 (30.0 %) United Kingdom of Great Britain and Northern Ireland: 3/10 (30.0 %) France: 2/10 (20.0 %) Ireland: 1/10 (10.0 %) Belgium: 1/10 (10.0 %) |
| **Co-reported drugs** | Vitamin d nos: 1/ 5 (20.0 %) Fish oil: 1/ 5 (20.0 %) Ondansetron hydrochloride: 1/ 5 (20.0 %) Fluoxetine: 1/ 5 (20.0 %) Estradiol;Estriol: 1/ 5 (20.0 %) | Methotrexate: 8/ 30 (26.7 %) Methotrexate sodium: 8/ 30 (26.7 %) Mycophenolate mofetil: 5/ 30 (16.7 %) Azathioprine: 5/ 30 (16.7 %) Hydroxychloroquine: 5/ 30 (16.7 %) | Desogestrel: 1/ 10 (10.0 %) Isotretinoin: 1/ 10 (10.0 %) Oral contraceptive nos: 1/ 10 (10.0 %) Erythromycin;Zinc acetate: 1/ 10 (10.0 %) Mitoxantrone hydrochloride: 1/ 10 (10.0 %) |
| **Co-reported reactions** | Chest discomfort: 1/5 (20.0 %) Hypoaesthesia oral: 1/5 (20.0 %) Hypoaesthesia: 1/5 (20.0 %) Pallor: 1/5 (20.0 %) Nasopharyngitis: 1/5 (20.0 %) | Arthralgia: 16/30 (53.3 %) Fatigue: 11/30 (36.7 %) Pain: 10/30 (33.3 %) Nausea: 8/30 (26.7 %) Pain in extremity: 7/30 (23.3 %) | Arthralgia: 5/10 (50.0 %) Systemic lupus erythematosus: 3/10 (30.0 %) Fatigue: 2/10 (20.0 %) Rash: 1/10 (10.0 %) Weight decreased: 1/10 (10.0 %) |
| **Reporter qualification** | Healthcare professional : 3/5 (60.0 %) Non healthcare professional : 2/5 (40.0 %) | Healthcare professional : 20/30 (66.67 %) Non healthcare professional : 10/30 (33.33 %) | Healthcare professional : 4/10 (40.0 %) Non healthcare professional : 6/10 (60.0 %) |
| **Indications** | Migraine : 4/5 (80.0 %) Unknown : 2/5 (40.0 %) | Unknown : 13/30 (43.3 %) Product used for unknown indication : 4/30 (13.3 %) Systemic lupus erythematosus : 3/30 (10.0 %) Drug use for unknown indication : 3/30 (10.0 %) Lupus nephritis : 2/30 (6.7 %) | Unknown : 6/10 (60.0 %) Acne : 3/10 (30.0 %) Arthritis reactive : 1/10 (10.0 %) |
| **Mean Time to Onset (std)** | nan | nan | 460 days (353 days) |

|  | **Methotrexate** | **Amfetamine** | **Mycophenolic acid** |
| --- | --- | --- | --- |
| **Number of cases** | 69 | 5 | 25 |
| **IC025_RP_main** | 0.82 | 0.77 | 0.58 |
| **IC025_RP_comparator** | 0.45 | -2.57 | 0.17 |
| **IC025_RP_healthcare** | 0.66 | -3.15 | 0.3 |
| **IC025_RP_malefemale** | 0.5 | -3.15 | 0.67 |
| **IC025_RP-Broad_main** | 1.79 | 2.0 | 1.17 |
| **Sex (Female / Male / Unknown)** | 58 / 8 / 3 | 3 / 1 / 1 | 19 / 5 / 1 |
| **Mean age (SD) in years** | 48.09 (15.32) years | 19.0 (nan) years | 48.0 (13.55) years |
| **Mean number of suspect/interacting drugs (SD)** | 4.73 (4.61) | 7.0 (nan) | 7.64 (7.14) |
| **Withdrawn** | 13/69 (18.84 %) | 2/5 (40.0 %) | 0/25 (0.0 %) |
| **Outcome after drug withdrawal** | Recovered: 3/13 (23.08 %) Not recovered: 2/13 (15.38 %) Evolution Unknown: 8/13 (61.54 %) | Recovering: 1/2 (50.0 %) Not recovered: 1/2 (50.0 %) |  |
| **Country of primary source** | United States of America: 34/69 (49.3 %) Germany: 11/69 (15.9 %) Canada: 7/69 (10.1 %) France: 5/69 (7.2 %) United Kingdom of Great Britain and Northern Ireland: 2/69 (2.9 %) | United States of America: 5/5 (100.0 %) | United States of America: 15/25 (60.0 %) Canada: 6/25 (24.0 %) France: 2/25 (8.0 %) Austria: 1/25 (4.0 %) Poland: 1/25 (4.0 %) |
| **Co-reported drugs** | Etanercept: 21/ 69 (30.4 %) Folic acid: 15/ 69 (21.7 %) Prednisone: 11/ 69 (15.9 %) Adalimumab: 9/ 69 (13.0 %) Hydroxychloroquine: 8/ 69 (11.6 %) | Propranolol: 1/ 5 (20.0 %) Erenumab: 1/ 5 (20.0 %) Ondansetron: 1/ 5 (20.0 %) Galcanezumab: 1/ 5 (20.0 %) Oxycodone: 1/ 5 (20.0 %) | Rituximab: 9/ 25 (36.0 %) Prednisone: 9/ 25 (36.0 %) Hydroxychloroquine: 7/ 25 (28.0 %) Hydroxychloroquine sulfate: 7/ 25 (28.0 %) Tocilizumab: 5/ 25 (20.0 %) |
| **Co-reported reactions** | Fatigue: 21/69 (30.4 %) Arthralgia: 18/69 (26.1 %) Drug ineffective: 14/69 (20.3 %) Nausea: 13/69 (18.8 %) Pain: 13/69 (18.8 %) | Burning sensation: 2/5 (40.0 %) Skin discolouration: 2/5 (40.0 %) Sensory loss: 2/5 (40.0 %) Mobility decreased: 1/5 (20.0 %) Off label use: 1/5 (20.0 %) | Off label use: 9/25 (36.0 %) Arthralgia: 8/25 (32.0 %) Fatigue: 7/25 (28.0 %) Weight decreased: 5/25 (20.0 %) Dyspnoea: 5/25 (20.0 %) |
| **Reporter qualification** | Healthcare professional : 54/69 (78.26 %) Non healthcare professional : 15/69 (21.74 %) | Healthcare professional : 1/5 (20.0 %) Non healthcare professional : 4/5 (80.0 %) | Healthcare professional : 20/25 (80.0 %) Non healthcare professional : 5/25 (20.0 %) |
| **Indications** | Product used for unknown indication : 22/69 (31.9 %) Rheumatoid arthritis : 19/69 (27.5 %) Unknown : 12/69 (17.4 %) Drug use for unknown indication : 3/69 (4.3 %) Psoriatic arthropathy : 2/69 (2.9 %) | Product used for unknown indication : 3/5 (60.0 %) Attention deficit hyperactivity disorder : 1/5 (20.0 %) Unknown : 1/5 (20.0 %) | Product used for unknown indication : 6/25 (24.0 %) Unknown : 5/25 (20.0 %) Drug use for unknown indication : 5/25 (20.0 %) Scleroderma : 4/25 (16.0 %) Interstitial lung disease : 3/25 (12.0 %) |
| **Mean Time to Onset (std)** | 1019 days (1047 days) | nan | 463 days (310 days) |

|  | **Betaxolol** | **Raloxifene** | **Dienogest;Ethinylestradiol** |
| --- | --- | --- | --- |
| **Number of cases** | 4 | 9 | 5 |
| **IC025_RP_main** | 0.74 | 0.79 | 0.71 |
| **IC025_RP_comparator** | -1.09 | 0.2 | 0.44 |
| **IC025_RP_healthcare** | 0.45 | -0.16 | -0.03 |
| **IC025_RP_malefemale** | 1.0 | -2.19 | 0.14 |
| **IC025_RP-Broad_main** | 3.05 | 1.1 | 0.24 |
| **Sex (Female / Male / Unknown)** | 2 / 2 / 0 | 9 / 0 / 0 | 5 / 0 / 0 |
| **Mean age (SD) in years** | 59.25 (5.74) years | 68.5 (6.36) years | 27.5 (7.05) years |
| **Mean number of suspect/interacting drugs (SD)** | 1.0 (0.0) | 3.0 (1.73) | 1.0 (nan) |
| **Withdrawn** | 3/4 (75.0 %) | 3/9 (33.33 %) | 2/5 (40.0 %) |
| **Outcome after drug withdrawal** | Recovered: 1/3 (33.33 %) Not recovered: 2/3 (66.67 %) | Recovering: 1/3 (33.33 %) Not recovered: 1/3 (33.33 %) Evolution Unknown: 1/3 (33.33 %) | Recovered: 1/2 (50.0 %) Recovering: 1/2 (50.0 %) |
| **Country of primary source** | France: 2/4 (50.0 %) Tunisia: 1/4 (25.0 %) Sweden: 1/4 (25.0 %) | United States of America: 8/9 (88.9 %) Belgium: 1/9 (11.1 %) | Germany: 3/5 (60.0 %) Spain: 2/5 (40.0 %) |
| **Co-reported drugs** | Timolol maleate: 1/ 4 (25.0 %) Hydrochlorothiazide;Quinapril hydrochloride: 1/ 4 (25.0 %) | Vitamin b complex: 1/ 9 (11.1 %) Fish oil: 1/ 9 (11.1 %) Magnesium: 1/ 9 (11.1 %) Calcium: 1/ 9 (11.1 %) Colecalciferol: 1/ 9 (11.1 %) | Drospirenone;Ethinylestradiol: 1/ 5 (20.0 %) |
| **Co-reported reactions** | Depression: 1/4 (25.0 %) Suicidal ideation: 1/4 (25.0 %) Morbid thoughts: 1/4 (25.0 %) Chest discomfort: 1/4 (25.0 %) Muscular weakness: 1/4 (25.0 %) | Insomnia: 1/9 (11.1 %) Muscle spasms: 1/9 (11.1 %) White blood cell count increased: 1/9 (11.1 %) White blood cells urine positive: 1/9 (11.1 %) Tremor: 1/9 (11.1 %) | Hypoaesthesia: 1/5 (20.0 %) Peripheral coldness: 1/5 (20.0 %) Peripheral vascular disorder: 1/5 (20.0 %) Pallor: 1/5 (20.0 %) Cystitis: 1/5 (20.0 %) |
| **Reporter qualification** | Healthcare professional : 3/4 (75.0 %) Non healthcare professional : 1/4 (25.0 %) | Healthcare professional : 4/9 (44.44 %) Non healthcare professional : 5/9 (55.56 %) | Healthcare professional : 2/5 (40.0 %) Non healthcare professional : 3/5 (60.0 %) |
| **Indications** | Essential hypertension, unspecified : 2/4 (50.0 %) Glaucoma : 1/4 (25.0 %) Essential benign hypertension : 1/4 (25.0 %) | Unknown : 6/9 (66.7 %) Prophylaxis : 2/9 (22.2 %) Breast cancer : 1/9 (11.1 %) Osteoporosis : 1/9 (11.1 %) | Contraception : 2/5 (40.0 %) Oral contraception : 2/5 (40.0 %) Acne : 1/5 (20.0 %) |
| **Mean Time to Onset (std)** | 321 days (277 days) | nan | 150 days (77 days) |

|  | **Nortriptyline** | **Dexmethylphenidate;Serdexmethylphenidate** | **Gemcitabine** |
| --- | --- | --- | --- |
| **Number of cases** | 6 | 3 | 33 |
| **IC025_RP_main** | 0.46 | 0.59 | 0.77 |
| **IC025_RP_comparator** | 0.22 | -0.81 | 0.18 |
| **IC025_RP_healthcare** | -0.07 | -0.35 | 0.87 |
| **IC025_RP_malefemale** | -0.16 | -2.28 | 0.82 |
| **IC025_RP-Broad_main** | 3.38 | 0.4 | 1.72 |
| **Sex (Female / Male / Unknown)** | 5 / 1 / 0 | 1 / 0 / 2 | 17 / 15 / 1 |
| **Mean age (SD) in years** | 51.75 (17.11) years | 29.0 (nan) years | 64.19 (9.43) years |
| **Mean number of suspect/interacting drugs (SD)** | 3.83 (3.06) | nan (nan) | 3.25 (3.3) |
| **Withdrawn** | 3/6 (50.0 %) | 2/3 (66.67 %) | 6/33 (18.18 %) |
| **Outcome after drug withdrawal** | Recovered: 1/3 (33.33 %) Evolution Unknown: 2/3 (66.67 %) | Evolution Unknown: 2/2 (100.0 %) | Recovering: 1/6 (16.67 %) Not recovered: 2/6 (33.33 %) Died: 1/6 (16.67 %) Evolution Unknown: 2/6 (33.33 %) |
| **Country of primary source** | Netherlands: 3/6 (50.0 %) United States of America: 1/6 (16.7 %) Belgium: 1/6 (16.7 %) Germany: 1/6 (16.7 %) | United States of America: 3/3 (100.0 %) | United States of America: 10/33 (30.3 %) France: 8/33 (24.2 %) Canada: 6/33 (18.2 %) United Kingdom of Great Britain and Northern Ireland: 4/33 (12.1 %) Switzerland: 2/33 (6.1 %) |
| **Co-reported drugs** | Drospirenone;Ethinylestradiol: 1/ 6 (16.7 %) Valproate sodium: 1/ 6 (16.7 %) Calcium carbonate;Colecalciferol: 1/ 6 (16.7 %) Indapamide: 1/ 6 (16.7 %) Atorvastatin calcium: 1/ 6 (16.7 %) |  | Carboplatin: 7/ 33 (21.2 %) Cisplatin: 3/ 33 (9.1 %) Furosemide: 2/ 33 (6.1 %) Ondansetron: 2/ 33 (6.1 %) Paracetamol: 2/ 33 (6.1 %) |
| **Co-reported reactions** | Orthostatic hypotension: 2/6 (33.3 %) Product use in unapproved indication: 2/6 (33.3 %) Dizziness: 1/6 (16.7 %) Electric shock sensation: 1/6 (16.7 %) Tinnitus: 1/6 (16.7 %) |  | Condition aggravated: 4/33 (12.1 %) Malignant neoplasm progression: 3/33 (9.1 %) Anaemia: 3/33 (9.1 %) Skin discolouration: 3/33 (9.1 %) Necrosis: 3/33 (9.1 %) |
| **Reporter qualification** | Healthcare professional : 4/6 (66.67 %) Non healthcare professional : 2/6 (33.33 %) | Healthcare professional : 2/3 (66.67 %) Non healthcare professional : 1/3 (33.33 %) | Healthcare professional : 27/33 (81.82 %) Non healthcare professional : 6/33 (18.18 %) |
| **Indications** | Depression : 2/6 (33.3 %) Prophylaxis : 1/6 (16.7 %) Polyneuropathy : 1/6 (16.7 %) Migraine : 1/6 (16.7 %) Chronic migraine : 1/6 (16.7 %) | Product used for unknown indication : 2/3 (66.7 %) Attention deficit hyperactivity disorder : 1/3 (33.3 %) Anxiety : 1/3 (33.3 %) | Unknown : 14/33 (42.4 %) Cholangiocarcinoma : 6/33 (18.2 %) Pancreatic carcinoma : 3/33 (9.1 %) Pancreas cancer : 1/33 (3.0 %) Pancreatic adenocarcinoma metastatic : 1/33 (3.0 %) |
| **Mean Time to Onset (std)** | 1 days (2 days) | nan | 163 days (223 days) |

|  | **Vinblastine** | **Pamidronic acid** | **Collagenase clostridium histolyticum** |
| --- | --- | --- | --- |
| **Number of cases** | 6 | 6 | 5 |
| **IC025_RP_main** | 0.7 | 0.7 | 0.59 |
| **IC025_RP_comparator** | 1.17 | -0.34 | 0.25 |
| **IC025_RP_healthcare** | 0.34 | -1.59 | 0.76 |
| **IC025_RP_malefemale** | 0.62 | -1.54 | 0.21 |
| **IC025_RP-Broad_main** | 2.24 | 0.96 | 0.41 |
| **Sex (Female / Male / Unknown)** | 3 / 3 / 0 | 5 / 1 / 0 | 1 / 4 / 0 |
| **Mean age (SD) in years** | 38.6 (8.91) years | 69.5 (3.54) years | 63.0 (7.0) years |
| **Mean number of suspect/interacting drugs (SD)** | 5.5 (3.33) | 25.0 (19.83) | 8.0 (nan) |
| **Withdrawn** | 3/6 (50.0 %) | 4/6 (66.67 %) | 1/5 (20.0 %) |
| **Outcome after drug withdrawal** | Not recovered: 3/3 (100.0 %) | Evolution Unknown: 3/4 (75.0 %) | Recovering: 1/1 (100.0 %) |
| **Country of primary source** | France: 4/6 (66.7 %) Finland: 1/6 (16.7 %) Switzerland: 1/6 (16.7 %) | United States of America: 5/6 (83.3 %) Germany: 1/6 (16.7 %) | United States of America: 4/5 (80.0 %) Japan: 1/5 (20.0 %) |
| **Co-reported drugs** | Doxorubicin hydrochloride: 6/ 6 (100.0 %) Doxorubicin: 6/ 6 (100.0 %) Dacarbazine: 6/ 6 (100.0 %) Dacarbazine citrate: 6/ 6 (100.0 %) Bleomycin: 6/ 6 (100.0 %) | Zoledronic acid monohydrate: 4/ 6 (66.7 %) Zolpidem tartrate: 4/ 6 (66.7 %) Zolpidem: 4/ 6 (66.7 %) Warfarin sodium: 4/ 6 (66.7 %) Warfarin: 4/ 6 (66.7 %) | Losartan potassium: 2/ 5 (40.0 %) Losartan: 2/ 5 (40.0 %) Hydrochlorothiazide: 1/ 5 (20.0 %) Acetylsalicylic acid: 1/ 5 (20.0 %) Influenza vaccine: 1/ 5 (20.0 %) |
| **Co-reported reactions** | Paraesthesia: 1/6 (16.7 %) Skin lesion: 1/6 (16.7 %) Oedema peripheral: 1/6 (16.7 %) Hypoacusis: 1/6 (16.7 %) Chest pain: 1/6 (16.7 %) | Osteonecrosis of jaw: 5/6 (83.3 %) Injury: 5/6 (83.3 %) Spinal osteoarthritis: 5/6 (83.3 %) Pain in extremity: 5/6 (83.3 %) Dyspnoea: 5/6 (83.3 %) | Off label use: 1/5 (20.0 %) Anaphylactic reaction: 1/5 (20.0 %) |
| **Reporter qualification** | Healthcare professional : 5/6 (83.33 %) Non healthcare professional : 1/6 (16.67 %) | Healthcare professional : 2/6 (33.33 %) Non healthcare professional : 4/6 (66.67 %) | Healthcare professional : 4/5 (80.0 %) Non healthcare professional : 1/5 (20.0 %) |
| **Indications** | Hodgkin's lymphoma : 3/6 (50.0 %) Classical Hodgkin lymphoma : 1/6 (16.7 %) Unknown : 1/6 (16.7 %) Hodgkin's disease : 1/6 (16.7 %) | Unknown : 3/6 (50.0 %) Plasma cell myeloma : 1/6 (16.7 %) Osteoporosis : 1/6 (16.7 %) Breast cancer recurrent : 1/6 (16.7 %) Neoplasm malignant : 1/6 (16.7 %) | Dupuytren's contracture : 5/5 (100.0 %) Unknown : 2/5 (40.0 %) |
| **Mean Time to Onset (std)** | 157 days (85 days) | nan | nan |

|  | **Ribavirin** | **Armodafinil** | **Etanercept** |
| --- | --- | --- | --- |
| **Number of cases** | 32 | 5 | 148 |
| **IC025_RP_main** | 0.63 | 0.55 | 0.57 |
| **IC025_RP_comparator** | 1.67 | -2.93 | 0.15 |
| **IC025_RP_healthcare** | 0.77 | 0.04 | 0.76 |
| **IC025_RP_malefemale** | 0.66 | 0.16 | 0.39 |
| **IC025_RP-Broad_main** | 0.04 | 3.53 | -0.27 |
| **Sex (Female / Male / Unknown)** | 20 / 12 / 0 | 4 / 1 / 0 | 120 / 19 / 9 |
| **Mean age (SD) in years** | 51.26 (9.37) years | 35.0 (11.53) years | 52.6 (14.03) years |
| **Mean number of suspect/interacting drugs (SD)** | 3.75 (5.12) | 13.4 (11.87) | 5.69 (6.82) |
| **Withdrawn** | 7/32 (21.88 %) | 2/5 (40.0 %) | 29/148 (19.59 %) |
| **Outcome after drug withdrawal** | Recovered: 1/7 (14.29 %) Recovering: 1/7 (14.29 %) Not recovered: 2/7 (28.57 %) Evolution Unknown: 2/7 (28.57 %) | Recovered: 1/2 (50.0 %) Evolution Unknown: 1/2 (50.0 %) | Recovered: 8/29 (27.59 %) Recovering: 1/29 (3.45 %) Not recovered: 5/29 (17.24 %) Evolution Unknown: 12/29 (41.38 %) |
| **Country of primary source** | United States of America: 18/32 (56.2 %) France: 7/32 (21.9 %) Germany: 2/32 (6.2 %) Norway: 1/32 (3.1 %) Spain: 1/32 (3.1 %) | United States of America: 5/5 (100.0 %) | United States of America: 119/148 (80.4 %) Germany: 7/148 (4.7 %) United Kingdom of Great Britain and Northern Ireland: 6/148 (4.1 %) Canada: 4/148 (2.7 %) Colombia: 3/148 (2.0 %) |
| **Co-reported drugs** | Peginterferon alfa-2a: 14/ 32 (43.8 %) Peginterferon alfa-2b: 12/ 32 (37.5 %) Interferon alfa-2b: 5/ 32 (15.6 %) Prednisone: 4/ 32 (12.5 %) Mirtazapine: 3/ 32 (9.4 %) | Oxybate sodium: 4/ 5 (80.0 %) Naproxen sodium: 2/ 5 (40.0 %) Naproxen: 2/ 5 (40.0 %) Amfetamine aspartate;Amfetamine sulfate;Dexamfetamine saccharate;Dexamfetamine sulfate: 2/ 5 (40.0 %) Curcuma longa rhizome: 2/ 5 (40.0 %) | Methotrexate: 50/ 148 (33.8 %) Methotrexate sodium: 50/ 148 (33.8 %) Prednisone: 18/ 148 (12.2 %) Folic acid: 17/ 148 (11.5 %) Adalimumab: 14/ 148 (9.5 %) |
| **Co-reported reactions** | Fatigue: 7/32 (21.9 %) Headache: 6/32 (18.8 %) Fibromyalgia: 6/32 (18.8 %) Alopecia: 5/32 (15.6 %) Pyrexia: 4/32 (12.5 %) | Back pain: 2/5 (40.0 %) Headache: 2/5 (40.0 %) Ovarian cyst: 2/5 (40.0 %) Spinal stenosis: 1/5 (20.0 %) Dry mouth: 1/5 (20.0 %) | Arthralgia: 35/148 (23.6 %) Fatigue: 25/148 (16.9 %) Pain in extremity: 21/148 (14.2 %) Drug ineffective: 20/148 (13.5 %) Rheumatoid arthritis: 19/148 (12.8 %) |
| **Reporter qualification** | Healthcare professional : 23/32 (71.88 %) Non healthcare professional : 9/32 (28.12 %) | Healthcare professional : 3/5 (60.0 %) Non healthcare professional : 2/5 (40.0 %) | Healthcare professional : 109/148 (73.65 %) Non healthcare professional : 39/148 (26.35 %) |
| **Indications** | Hepatitis C : 14/32 (43.8 %) Unknown : 13/32 (40.6 %) Chronic hepatitis C : 4/32 (12.5 %) Hepatitis C virus test : 1/32 (3.1 %) Hepatitis viral : 1/32 (3.1 %) | Product used for unknown indication : 4/5 (80.0 %) Unknown : 1/5 (20.0 %) | Rheumatoid arthritis : 91/148 (61.5 %) Unknown : 19/148 (12.8 %) Psoriasis : 15/148 (10.1 %) Product used for unknown indication : 14/148 (9.5 %) Psoriatic arthropathy : 13/148 (8.8 %) |
| **Mean Time to Onset (std)** | 179 days (147 days) | nan | 924 days (1096 days) |

|  | **Tocilizumab** | **Drospirenone;Ethinylestradiol** | **Aripiprazole** |
| --- | --- | --- | --- |
| **Number of cases** | 26 | 20 | 26 |
| **IC025_RP_main** | 0.6 | 0.53 | 0.53 |
| **IC025_RP_comparator** | 0.19 | 0.35 | 1.36 |
| **IC025_RP_healthcare** | 0.62 | 0.31 | 0.3 |
| **IC025_RP_malefemale** | 0.77 | 0.08 | 1.0 |
| **IC025_RP-Broad_main** | 0.57 | 0.88 | 0.4 |
| **Sex (Female / Male / Unknown)** | 25 / 1 / 0 | 20 / 0 / 0 | 21 / 5 / 0 |
| **Mean age (SD) in years** | 58.94 (16.12) years | 26.19 (6.27) years | 39.62 (19.27) years |
| **Mean number of suspect/interacting drugs (SD)** | 6.95 (5.25) | 3.4 (4.83) | 3.17 (3.16) |
| **Withdrawn** | 8/26 (30.77 %) | 11/20 (55.0 %) | 15/26 (57.69 %) |
| **Outcome after drug withdrawal** | Recovered: 1/8 (12.5 %) Recovering: 1/8 (12.5 %) Not recovered: 1/8 (12.5 %) Evolution Unknown: 3/8 (37.5 %) | Recovered: 3/11 (27.27 %) Recovering: 2/11 (18.18 %) Not recovered: 2/11 (18.18 %) Evolution Unknown: 2/11 (18.18 %) | Recovered: 10/15 (66.67 %) Recovering: 1/15 (6.67 %) Recovered with sequelae: 1/15 (6.67 %) Not recovered: 2/15 (13.33 %) |
| **Country of primary source** | United States of America: 11/26 (42.3 %) Canada: 7/26 (26.9 %) France: 3/26 (11.5 %) Australia: 2/26 (7.7 %) Austria: 1/26 (3.8 %) | United States of America: 6/20 (30.0 %) Germany: 4/20 (20.0 %) Australia: 2/20 (10.0 %) Canada: 2/20 (10.0 %) United Kingdom of Great Britain and Northern Ireland: 2/20 (10.0 %) | Spain: 8/26 (30.8 %) United States of America: 6/26 (23.1 %) France: 3/26 (11.5 %) Türkiye: 2/26 (7.7 %) Netherlands: 2/26 (7.7 %) |
| **Co-reported drugs** | Prednisone: 9/ 26 (34.6 %) Hydroxychloroquine: 7/ 26 (26.9 %) Hydroxychloroquine sulfate: 7/ 26 (26.9 %) Methotrexate: 7/ 26 (26.9 %) Paracetamol: 7/ 26 (26.9 %) | Amfetamine aspartate;Amfetamine sulfate;Dexamfetamine saccharate;Dexamfetamine sulfate: 2/ 20 (10.0 %) Finasteride: 1/ 20 (5.0 %) Leuprorelin acetate: 1/ 20 (5.0 %) Dienogest;Ethinylestradiol: 1/ 20 (5.0 %) Levonorgestrel: 1/ 20 (5.0 %) | Methylphenidate hydrochloride: 3/ 26 (11.5 %) Bupropion hydrobromide: 3/ 26 (11.5 %) Bupropion hydrochloride: 3/ 26 (11.5 %) Escitalopram oxalate: 2/ 26 (7.7 %) Sertraline hydrochloride: 2/ 26 (7.7 %) |
| **Co-reported reactions** | Arthralgia: 7/26 (26.9 %) Rheumatoid arthritis: 7/26 (26.9 %) Pain: 6/26 (23.1 %) Drug ineffective: 6/26 (23.1 %) Malaise: 5/26 (19.2 %) | Arthralgia: 4/20 (20.0 %) Paraesthesia: 3/20 (15.0 %) Systemic lupus erythematosus: 3/20 (15.0 %) Migraine: 3/20 (15.0 %) Alopecia: 3/20 (15.0 %) | Pain in extremity: 2/26 (7.7 %) Cough: 2/26 (7.7 %) Vomiting: 2/26 (7.7 %) Abdominal pain: 2/26 (7.7 %) Obsessive-compulsive disorder: 2/26 (7.7 %) |
| **Reporter qualification** | Healthcare professional : 19/26 (73.08 %) Non healthcare professional : 7/26 (26.92 %) | Healthcare professional : 10/20 (50.0 %) Non healthcare professional : 10/20 (50.0 %) | Healthcare professional : 16/26 (61.54 %) Non healthcare professional : 10/26 (38.46 %) |
| **Indications** | Rheumatoid arthritis : 17/26 (65.4 %) Unknown : 13/26 (50.0 %) Horton's disease : 2/26 (7.7 %) Osteoarthritis : 1/26 (3.8 %) Felty's syndrome : 1/26 (3.8 %) | Unknown : 6/20 (30.0 %) Contraception : 5/20 (25.0 %) Oral contraception : 4/20 (20.0 %) Drug use for unknown indication : 2/20 (10.0 %) Acne : 2/20 (10.0 %) | Bipolar disorder : 4/26 (15.4 %) Schizoaffective disorder : 4/26 (15.4 %) Psychotic disorder : 3/26 (11.5 %) Depression : 2/26 (7.7 %) Psychotic episode : 2/26 (7.7 %) |
| **Mean Time to Onset (std)** | 509 days (640 days) | 474 days (581 days) | 253 days (437 days) |

|  | **Golimumab** | **Nilotinib** | **Selpercatinib** |
| --- | --- | --- | --- |
| **Number of cases** | 19 | 13 | 3 |
| **IC025_RP_main** | 0.49 | 0.47 | 0.33 |
| **IC025_RP_comparator** | 0.09 | 0.43 | 0.31 |
| **IC025_RP_healthcare** | -0.02 | 0.73 | 0.38 |
| **IC025_RP_malefemale** | -0.29 | 0.39 | -0.48 |
| **IC025_RP-Broad_main** | -0.32 | 1.98 | 0.57 |
| **Sex (Female / Male / Unknown)** | 12 / 4 / 3 | 6 / 6 / 1 | 1 / 1 / 1 |
| **Mean age (SD) in years** | 49.92 (8.39) years | 52.75 (13.87) years | 58.0 (2.83) years |
| **Mean number of suspect/interacting drugs (SD)** | 3.5 (2.32) | 2.67 (1.53) | 1.0 (nan) |
| **Withdrawn** | 7/19 (36.84 %) | 5/13 (38.46 %) | 1/3 (33.33 %) |
| **Outcome after drug withdrawal** | Recovered: 2/7 (28.57 %) Not recovered: 2/7 (28.57 %) Evolution Unknown: 3/7 (42.86 %) | Recovered: 1/5 (20.0 %) Recovering: 1/5 (20.0 %) Evolution Unknown: 2/5 (40.0 %) | Recovered: 1/1 (100.0 %) |
| **Country of primary source** | United States of America: 7/19 (36.8 %) United Kingdom of Great Britain and Northern Ireland: 5/19 (26.3 %) Canada: 2/19 (10.5 %) Ireland: 2/19 (10.5 %) Bulgaria: 1/19 (5.3 %) | Germany: 3/13 (23.1 %) United States of America: 3/13 (23.1 %) Spain: 2/13 (15.4 %) France: 2/13 (15.4 %) Malaysia: 1/13 (7.7 %) | United States of America: 2/3 (66.7 %) Netherlands: 1/3 (33.3 %) |
| **Co-reported drugs** | Methotrexate: 5/ 19 (26.3 %) Pantoprazole: 2/ 19 (10.5 %) Adalimumab: 2/ 19 (10.5 %) Etanercept: 2/ 19 (10.5 %) Terbinafine hydrochloride: 1/ 19 (5.3 %) | Vitamin d nos: 1/ 13 (7.7 %) Levothyroxine sodium;Potassium iodide: 1/ 13 (7.7 %) Ibandronate sodium: 1/ 13 (7.7 %) Imatinib mesilate: 1/ 13 (7.7 %) Hydroxycarbamide: 1/ 13 (7.7 %) | Levothyroxine sodium: 1/ 3 (33.3 %) |
| **Co-reported reactions** | Rash: 3/19 (15.8 %) Off label use: 2/19 (10.5 %) Urinary tract infection: 2/19 (10.5 %) Pain: 2/19 (10.5 %) Depression: 2/19 (10.5 %) | Fatigue: 2/13 (15.4 %) Intermittent claudication: 2/13 (15.4 %) Hyperbilirubinaemia: 2/13 (15.4 %) Peripheral arterial occlusive disease: 2/13 (15.4 %) Pain: 2/13 (15.4 %) | Abdominal distension: 1/3 (33.3 %) Incorrect dose administered: 1/3 (33.3 %) Fat necrosis: 1/3 (33.3 %) Enteritis: 1/3 (33.3 %) Chest pain: 1/3 (33.3 %) |
| **Reporter qualification** | Healthcare professional : 10/19 (52.63 %) Non healthcare professional : 9/19 (47.37 %) | Healthcare professional : 11/13 (84.62 %) Non healthcare professional : 2/13 (15.38 %) | Healthcare professional : 3/3 (100.0 %) Non healthcare professional : 0/3 (0.0 %) |
| **Indications** | Rheumatoid arthritis : 8/19 (42.1 %) Drug use for unknown indication : 4/19 (21.1 %) Crohn's disease : 2/19 (10.5 %) Product used for unknown indication : 2/19 (10.5 %) Raynaud's phenomenon : 1/19 (5.3 %) | Unknown : 4/13 (30.8 %) Chronic myeloid leukemia : 3/13 (23.1 %) Chronic myeloid leukaemia : 2/13 (15.4 %) CML : 2/13 (15.4 %) Chronic myelocytic leukemia : 1/13 (7.7 %) | Product used for unknown indication : 1/3 (33.3 %) Thyroid carcinoma : 1/3 (33.3 %) Non-small cell lung cancer : 1/3 (33.3 %) |
| **Mean Time to Onset (std)** | 290 days (340 days) | 130 days (214 days) | 224 days (79 days) |

|  | **Zoledronic acid** | **Ponatinib** | **Anastrozole** |
| --- | --- | --- | --- |
| **Number of cases** | 21 | 6 | 9 |
| **IC025_RP_main** | 0.39 | 0.41 | 0.45 |
| **IC025_RP_comparator** | -0.93 | 0.38 | 0.35 |
| **IC025_RP_healthcare** | -0.29 | -0.12 | -0.62 |
| **IC025_RP_malefemale** | -0.18 | -1.48 | 0.52 |
| **IC025_RP-Broad_main** | 0.28 | 1.97 | -0.29 |
| **Sex (Female / Male / Unknown)** | 18 / 3 / 0 | 3 / 2 / 1 | 9 / 0 / 0 |
| **Mean age (SD) in years** | 72.27 (12.63) years | 61.5 (7.78) years | 61.62 (10.11) years |
| **Mean number of suspect/interacting drugs (SD)** | 15.0 (14.68) | 9.0 (7.55) | 4.67 (4.76) |
| **Withdrawn** | 4/21 (19.05 %) | 3/6 (50.0 %) | 5/9 (55.56 %) |
| **Outcome after drug withdrawal** | Evolution Unknown: 3/4 (75.0 %) | Evolution Unknown: 1/3 (33.33 %) | Recovered: 2/5 (40.0 %) Not recovered: 1/5 (20.0 %) Evolution Unknown: 2/5 (40.0 %) |
| **Country of primary source** | United States of America: 14/21 (66.7 %) United Kingdom of Great Britain and Northern Ireland: 3/21 (14.3 %) Portugal: 1/21 (4.8 %) Germany: 1/21 (4.8 %) Canada: 1/21 (4.8 %) | United States of America: 4/6 (66.7 %) Belgium: 2/6 (33.3 %) | United States of America: 5/9 (55.6 %) United Kingdom of Great Britain and Northern Ireland: 2/9 (22.2 %) Germany: 1/9 (11.1 %) France: 1/9 (11.1 %) |
| **Co-reported drugs** | Furosemide: 7/ 21 (33.3 %) Alendronate sodium: 6/ 21 (28.6 %) Warfarin sodium: 5/ 21 (23.8 %) Warfarin: 5/ 21 (23.8 %) Chlorhexidine: 5/ 21 (23.8 %) | Metoprolol tartrate: 2/ 6 (33.3 %) Metoprolol succinate: 2/ 6 (33.3 %) Blinatumomab: 1/ 6 (16.7 %) Acetylsalicylic acid: 1/ 6 (16.7 %) Budesonide;Formoterol fumarate;Glycopyrronium bromide: 1/ 6 (16.7 %) | Ascorbic acid;Biotin;Calcium;Calcium phosphate dibasic;Chromic chloride;Cupric oxide;Cyanocobalamin;Ferrous fumarate;Folic acid;Magnesium oxide;Manganese sulfate;Nickel sulfate;Nicotinamide;Pantothenic acid;Phytomenadione;Potassium chloride;Potassium iodide;Pyridoxine hydrochloride;Retinol;Riboflavin;Sodium metasilicate;Sodium molybdate;Sodium selenate;Thiamine mononitrate;Tocopheryl acetate;Vitamin d nos;Zinc oxide: 2/ 9 (22.2 %) Exemestane: 1/ 9 (11.1 %) Pantoprazole sodium sesquihydrate: 1/ 9 (11.1 %) Ubidecarenone: 1/ 9 (11.1 %) Levothyroxine sodium: 1/ 9 (11.1 %) |
| **Co-reported reactions** | Pain: 11/21 (52.4 %) Arthralgia: 10/21 (47.6 %) Anxiety: 9/21 (42.9 %) Osteonecrosis of jaw: 9/21 (42.9 %) Depression: 8/21 (38.1 %) | Vomiting: 2/6 (33.3 %) Blood pressure increased: 2/6 (33.3 %) Lipase increased: 1/6 (16.7 %) Inappropriate schedule of product administration: 1/6 (16.7 %) Neuralgia: 1/6 (16.7 %) | Arthritis: 2/9 (22.2 %) Rash: 2/9 (22.2 %) Dyspnoea: 2/9 (22.2 %) Paraesthesia: 2/9 (22.2 %) Pain in extremity: 2/9 (22.2 %) |
| **Reporter qualification** | Healthcare professional : 10/21 (47.62 %) Non healthcare professional : 11/21 (52.38 %) | Healthcare professional : 3/6 (50.0 %) Non healthcare professional : 3/6 (50.0 %) | Healthcare professional : 4/9 (44.44 %) Non healthcare professional : 5/9 (55.56 %) |
| **Indications** | Unknown : 9/21 (42.9 %) Osteoporosis : 8/21 (38.1 %) Product used for unknown indication : 3/21 (14.3 %) Plasma cell myeloma : 2/21 (9.5 %) Bone density decreased : 1/21 (4.8 %) | Chronic myeloid leukaemia : 4/6 (66.7 %) Unknown : 4/6 (66.7 %) Acute lymphocytic leukaemia : 1/6 (16.7 %) Chronic phase chronic myeloid leukemia : 1/6 (16.7 %) Chronic phase chronic myeloid leukaemia : 1/6 (16.7 %) | Breast cancer : 4/9 (44.4 %) Unknown : 2/9 (22.2 %) Breast carcinoma : 1/9 (11.1 %) Breast cancer stage I : 1/9 (11.1 %) Breast cancer female : 1/9 (11.1 %) |
| **Mean Time to Onset (std)** | 360 days (267 days) | 494 days (185 days) | 459 days (389 days) |

|  | **Peginterferon alfa-2a** | **Simvastatin** | **Infliximab** |
| --- | --- | --- | --- |
| **Number of cases** | 17 | 19 | 50 |
| **IC025_RP_main** | 0.4 | 0.11 | 0.35 |
| **IC025_RP_comparator** | -0.73 | 0.1 | -0.07 |
| **IC025_RP_healthcare** | 0.5 | -0.28 | 0.32 |
| **IC025_RP_malefemale** | 0.35 | 0.04 | -0.05 |
| **IC025_RP-Broad_main** | -0.2 | 2.8 | 0.2 |
| **Sex (Female / Male / Unknown)** | 11 / 6 / 0 | 12 / 6 / 1 | 32 / 15 / 3 |
| **Mean age (SD) in years** | 49.73 (8.92) years | 60.36 (8.07) years | 44.77 (16.88) years |
| **Mean number of suspect/interacting drugs (SD)** | 2.38 (2.16) | 2.83 (2.33) | 4.9 (4.37) |
| **Withdrawn** | 4/17 (23.53 %) | 10/19 (52.63 %) | 18/50 (36.0 %) |
| **Outcome after drug withdrawal** | Recovered: 1/4 (25.0 %) Recovered with sequelae: 1/4 (25.0 %) Evolution Unknown: 2/4 (50.0 %) | Recovered: 2/10 (20.0 %) Recovering: 1/10 (10.0 %) Not recovered: 2/10 (20.0 %) Evolution Unknown: 4/10 (40.0 %) | Recovered: 4/18 (22.22 %) Recovering: 2/18 (11.11 %) Not recovered: 3/18 (16.67 %) Evolution Unknown: 7/18 (38.89 %) |
| **Country of primary source** | United States of America: 10/17 (58.8 %) France: 2/17 (11.8 %) Germany: 1/17 (5.9 %) Italy: 1/17 (5.9 %) Türkiye: 1/17 (5.9 %) | United Kingdom of Great Britain and Northern Ireland: 6/19 (31.6 %) United States of America: 4/19 (21.1 %) Germany: 3/19 (15.8 %) Netherlands: 3/19 (15.8 %) France: 2/19 (10.5 %) | United States of America: 20/50 (40.0 %) Canada: 10/50 (20.0 %) France: 2/50 (4.0 %) Spain: 2/50 (4.0 %) United Kingdom of Great Britain and Northern Ireland: 2/50 (4.0 %) |
| **Co-reported drugs** | Ribavirin: 16/ 17 (94.1 %) Mirtazapine: 2/ 17 (11.8 %) Boceprevir: 1/ 17 (5.9 %) Temazepam: 1/ 17 (5.9 %) Fexofenadine hydrochloride: 1/ 17 (5.9 %) | Atenolol: 3/ 19 (15.8 %) Bisoprolol: 2/ 19 (10.5 %) Ramipril: 2/ 19 (10.5 %) Acetylsalicylic acid: 2/ 19 (10.5 %) Metformin hydrochloride: 1/ 19 (5.3 %) | Methotrexate: 10/ 50 (20.0 %) Folic acid: 7/ 50 (14.0 %) Methylprednisolone: 4/ 50 (8.0 %) Methylprednisolone sodium succinate: 4/ 50 (8.0 %) Paracetamol: 3/ 50 (6.0 %) |
| **Co-reported reactions** | Fatigue: 5/17 (29.4 %) Fibromyalgia: 4/17 (23.5 %) Headache: 3/17 (17.6 %) Leukopenia: 2/17 (11.8 %) Rash: 2/17 (11.8 %) | Myalgia: 4/19 (21.1 %) Arthralgia: 3/19 (15.8 %) Muscular weakness: 2/19 (10.5 %) Headache: 2/19 (10.5 %) Back pain: 2/19 (10.5 %) | Arthralgia: 9/50 (18.0 %) Headache: 8/50 (16.0 %) Infusion related reaction: 7/50 (14.0 %) Drug ineffective: 6/50 (12.0 %) Fatigue: 6/50 (12.0 %) |
| **Reporter qualification** | Healthcare professional : 11/17 (64.71 %) Non healthcare professional : 6/17 (35.29 %) | Healthcare professional : 10/19 (52.63 %) Non healthcare professional : 9/19 (47.37 %) | Healthcare professional : 38/50 (76.0 %) Non healthcare professional : 12/50 (24.0 %) |
| **Indications** | Hepatitis C : 7/17 (41.2 %) Chronic hepatitis C : 4/17 (23.5 %) Unknown : 4/17 (23.5 %) Hepatitis C virus test : 2/17 (11.8 %) Malignant melanoma of skin : 1/17 (5.9 %) | Unknown : 10/19 (52.6 %) Blood cholesterol increased : 2/19 (10.5 %) Pure hypercholesterolaemia : 2/19 (10.5 %) Product used for unknown indication : 1/19 (5.3 %) Heart attack : 1/19 (5.3 %) | Crohn's disease : 12/50 (24.0 %) Unknown : 10/50 (20.0 %) Rheumatoid arthritis : 7/50 (14.0 %) Product used for unknown indication : 3/50 (6.0 %) Ankylosing spondylitis : 3/50 (6.0 %) |
| **Mean Time to Onset (std)** | 162 days (172 days) | 132 days (240 days) | 252 days (314 days) |

|  | **Rituximab** | **Finasteride** | **Certolizumab pegol** |
| --- | --- | --- | --- |
| **Number of cases** | 33 | 8 | 24 |
| **IC025_RP_main** | 0.19 | 0.17 | 0.33 |
| **IC025_RP_comparator** | 0.01 | 0.74 | -0.08 |
| **IC025_RP_healthcare** | 0.12 | -0.16 | -0.06 |
| **IC025_RP_malefemale** | 0.08 | -0.88 | 0.74 |
| **IC025_RP-Broad_main** | 0.09 | 1.16 | -0.21 |
| **Sex (Female / Male / Unknown)** | 25 / 5 / 3 | 1 / 7 / 0 | 20 / 4 / 0 |
| **Mean age (SD) in years** | 50.38 (13.34) years | 54.67 (28.57) years | 51.75 (14.28) years |
| **Mean number of suspect/interacting drugs (SD)** | 9.97 (6.4) | 2.75 (0.5) | 4.77 (3.7) |
| **Withdrawn** | 3/33 (9.09 %) | 3/8 (37.5 %) | 18/24 (75.0 %) |
| **Outcome after drug withdrawal** | Recovered: 1/3 (33.33 %) Evolution Unknown: 2/3 (66.67 %) | Not recovered: 2/3 (66.67 %) | Recovered: 1/18 (5.56 %) Recovering: 2/18 (11.11 %) Not recovered: 7/18 (38.89 %) Evolution Unknown: 2/18 (11.11 %) |
| **Country of primary source** | United States of America: 15/33 (45.5 %) Canada: 12/33 (36.4 %) Germany: 2/33 (6.1 %) Romania: 1/33 (3.0 %) United Kingdom of Great Britain and Northern Ireland: 1/33 (3.0 %) | United States of America: 4/8 (50.0 %) Australia: 1/8 (12.5 %) France: 1/8 (12.5 %) Netherlands: 1/8 (12.5 %) United Kingdom of Great Britain and Northern Ireland: 1/8 (12.5 %) | United States of America: 14/24 (58.3 %) Switzerland: 3/24 (12.5 %) Canada: 3/24 (12.5 %) United Kingdom of Great Britain and Northern Ireland: 2/24 (8.3 %) France: 1/24 (4.2 %) |
| **Co-reported drugs** | Prednisone: 15/ 33 (45.5 %) Paracetamol: 12/ 33 (36.4 %) Hydroxychloroquine: 12/ 33 (36.4 %) Hydroxychloroquine sulfate: 12/ 33 (36.4 %) Diphenhydramine hydrochloride: 11/ 33 (33.3 %) | Drospirenone;Ethinylestradiol: 2/ 8 (25.0 %) Drospirenone;Ethinylestradiol betadex clathrate: 2/ 8 (25.0 %) Leuprorelin acetate: 1/ 8 (12.5 %) Fluoxetine: 1/ 8 (12.5 %) Alprazolam: 1/ 8 (12.5 %) | Methotrexate: 6/ 24 (25.0 %) Methotrexate sodium: 6/ 24 (25.0 %) Prednisone: 3/ 24 (12.5 %) Budesonide: 2/ 24 (8.3 %) Ondansetron hydrochloride: 2/ 24 (8.3 %) |
| **Co-reported reactions** | Fatigue: 11/33 (33.3 %) Dyspnoea: 10/33 (30.3 %) Arthralgia: 9/33 (27.3 %) Weight decreased: 7/33 (21.2 %) Rheumatoid arthritis: 7/33 (21.2 %) | Anxiety: 4/8 (50.0 %) Sexual dysfunction: 4/8 (50.0 %) Depression: 4/8 (50.0 %) Cognitive disorder: 3/8 (37.5 %) Erectile dysfunction: 3/8 (37.5 %) | Fatigue: 5/24 (20.8 %) Drug ineffective: 5/24 (20.8 %) Arthralgia: 4/24 (16.7 %) Sjogren's syndrome: 4/24 (16.7 %) Rheumatoid arthritis: 4/24 (16.7 %) |
| **Reporter qualification** | Healthcare professional : 29/33 (87.88 %) Non healthcare professional : 4/33 (12.12 %) | Healthcare professional : 4/8 (50.0 %) Non healthcare professional : 4/8 (50.0 %) | Healthcare professional : 14/24 (58.33 %) Non healthcare professional : 10/24 (41.67 %) |
| **Indications** | Rheumatoid arthritis : 12/33 (36.4 %) Unknown : 5/33 (15.2 %) Product used for unknown indication : 3/33 (9.1 %) Systemic lupus erythematosus : 3/33 (9.1 %) Systemic scleroderma : 2/33 (6.1 %) | Unknown : 6/8 (75.0 %) Alopecia : 2/8 (25.0 %) Androgenetic alopecia : 2/8 (25.0 %) Benign prostatic hyperplasia : 1/8 (12.5 %) Prostatic obstruction : 1/8 (12.5 %) | Rheumatoid arthritis : 11/24 (45.8 %) Unknown : 10/24 (41.7 %) Product used for unknown indication : 4/24 (16.7 %) Psoriasis : 2/24 (8.3 %) Ankylosing spondylitis : 2/24 (8.3 %) |
| **Mean Time to Onset (std)** | 1293 days (1340 days) | nan | 624 days (1122 days) |

|  | **Corticotropin** | **Dihydroergotamine** | **Amantadine** |
| --- | --- | --- | --- |
| **Number of cases** | 5 | 3 | 5 |
| **IC025_RP_main** | 0.26 | 0.21 | 0.28 |
| **IC025_RP_comparator** | 0.16 | -0.93 | 0.42 |
| **IC025_RP_healthcare** | -3.2 | 0.33 | 0.19 |
| **IC025_RP_malefemale** | -0.46 | 0.3 | 0.23 |
| **IC025_RP-Broad_main** | 0.01 | 4.86 | 3.0 |
| **Sex (Female / Male / Unknown)** | 5 / 0 / 0 | 3 / 0 / 0 | 3 / 2 / 0 |
| **Mean age (SD) in years** | 47.33 (22.59) years | 66.67 (28.38) years | 63.75 (17.0) years |
| **Mean number of suspect/interacting drugs (SD)** | 12.2 (11.82) | 3.0 (0.0) | 4.4 (2.79) |
| **Withdrawn** | 5/5 (100.0 %) | 0/3 (0.0 %) | 2/5 (40.0 %) |
| **Outcome after drug withdrawal** | Not recovered: 2/5 (40.0 %) |  | Not recovered: 1/2 (50.0 %) Evolution Unknown: 1/2 (50.0 %) |
| **Country of primary source** | United States of America: 5/5 (100.0 %) | France: 3/3 (100.0 %) | France: 2/5 (40.0 %) Switzerland: 1/5 (20.0 %) Netherlands: 1/5 (20.0 %) Germany: 1/5 (20.0 %) |
| **Co-reported drugs** | Insulin human isophane: 2/ 5 (40.0 %) Insulin human: 2/ 5 (40.0 %) Prednisone: 2/ 5 (40.0 %) Acetylsalicylic acid: 2/ 5 (40.0 %) Hydrocodone bitartrate;Paracetamol: 2/ 5 (40.0 %) | Spiramycin: 1/ 3 (33.3 %) Paracetamol: 1/ 3 (33.3 %) Ambroxol hydrochloride: 1/ 3 (33.3 %) Caffeine;Cyclizine hydrochloride;Ergotamine tartrate: 1/ 3 (33.3 %) Amylocaine hydrochloride;Caffeine;Codeine;Phenazone: 1/ 3 (33.3 %) | Rasagiline: 2/ 5 (40.0 %) Rasagiline mesylate: 2/ 5 (40.0 %) Levodopa: 2/ 5 (40.0 %) Candesartan cilexetil;Hydrochlorothiazide: 1/ 5 (20.0 %) Ribavirin: 1/ 5 (20.0 %) |
| **Co-reported reactions** | Injection site pruritus: 3/5 (60.0 %) Product dose omission issue: 3/5 (60.0 %) Systemic lupus erythematosus: 2/5 (40.0 %) Weight increased: 2/5 (40.0 %) Dyspnoea: 2/5 (40.0 %) | Vertigo: 1/3 (33.3 %) Headache: 1/3 (33.3 %) Peripheral coldness: 1/3 (33.3 %) Vasoconstriction: 1/3 (33.3 %) | Livedo reticularis: 3/5 (60.0 %) Oedema peripheral: 2/5 (40.0 %) Hypokinesia: 1/5 (20.0 %) Insomnia: 1/5 (20.0 %) Muscle spasms: 1/5 (20.0 %) |
| **Reporter qualification** | Healthcare professional : 1/5 (20.0 %) Non healthcare professional : 4/5 (80.0 %) | Healthcare professional : 3/3 (100.0 %) Non healthcare professional : 0/3 (0.0 %) | Healthcare professional : 4/5 (80.0 %) Non healthcare professional : 1/5 (20.0 %) |
| **Indications** | Systemic lupus erythematosus : 2/5 (40.0 %) Unknown : 2/5 (40.0 %) Multiple sclerosis : 1/5 (20.0 %) Product used for unknown indication : 1/5 (20.0 %) Dermatomyositis : 1/5 (20.0 %) | Unknown : 2/3 (66.7 %) Migraine, unspecified : 1/3 (33.3 %) | Parkinson's disease : 2/5 (40.0 %) Disease Parkinson's : 1/5 (20.0 %) Parkinson's disease NOS : 1/5 (20.0 %) Hepatitis in viral disease classified elsewhere : 1/5 (20.0 %) |
| **Mean Time to Onset (std)** | nan | nan | 70 days (40 days) |

|  | **Ocrelizumab** | **Omalizumab** | **Imatinib** |
| --- | --- | --- | --- |
| **Number of cases** | 14 | 16 | 20 |
| **IC025_RP_main** | 0.16 | 0.17 | 0.28 |
| **IC025_RP_comparator** | -0.23 | 0.67 | 0.24 |
| **IC025_RP_healthcare** | -0.42 | 0.42 | 0.28 |
| **IC025_RP_malefemale** | -0.92 | 0.44 | -0.19 |
| **IC025_RP-Broad_main** | -0.8 | -0.83 | 0.34 |
| **Sex (Female / Male / Unknown)** | 12 / 2 / 0 | 14 / 1 / 1 | 13 / 7 / 0 |
| **Mean age (SD) in years** | 34.83 (12.67) years | 43.58 (12.19) years | 60.3 (13.01) years |
| **Mean number of suspect/interacting drugs (SD)** | 8.1 (8.86) | 5.38 (4.66) | 2.5 (2.55) |
| **Withdrawn** | 1/14 (7.14 %) | 4/16 (25.0 %) | 3/20 (15.0 %) |
| **Outcome after drug withdrawal** | Recovered: 1/1 (100.0 %) | Not recovered: 2/4 (50.0 %) Evolution Unknown: 2/4 (50.0 %) | Died: 1/3 (33.33 %) Evolution Unknown: 2/3 (66.67 %) |
| **Country of primary source** | United States of America: 8/14 (57.1 %) Germany: 4/14 (28.6 %) Belgium: 1/14 (7.1 %) Italy: 1/14 (7.1 %) | United States of America: 5/16 (31.2 %) France: 2/16 (12.5 %) Spain: 2/16 (12.5 %) United Kingdom of Great Britain and Northern Ireland: 2/16 (12.5 %) Sweden: 1/16 (6.2 %) | United States of America: 6/20 (30.0 %) France: 3/20 (15.0 %) Germany: 3/20 (15.0 %) United Kingdom of Great Britain and Northern Ireland: 3/20 (15.0 %) Spain: 1/20 (5.0 %) |
| **Co-reported drugs** | Baclofen: 4/ 14 (28.6 %) Pregabalin: 3/ 14 (21.4 %) Elasomeran: 2/ 14 (14.3 %) Tozinameran: 2/ 14 (14.3 %) Gabapentin: 2/ 14 (14.3 %) | Fluticasone propionate;Salmeterol xinafoate: 3/ 16 (18.8 %) Montelukast: 3/ 16 (18.8 %) Montelukast sodium: 3/ 16 (18.8 %) Bilastine: 2/ 16 (12.5 %) Rupatadine fumarate: 2/ 16 (12.5 %) | Nilotinib hydrochloride monohydrate: 2/ 20 (10.0 %) Acetylsalicylic acid: 1/ 20 (5.0 %) Candesartan cilexetil: 1/ 20 (5.0 %) Enalapril maleate: 1/ 20 (5.0 %) Fenoterol hydrobromide;Ipratropium bromide: 1/ 20 (5.0 %) |
| **Co-reported reactions** | COVID-19: 4/14 (28.6 %) Off label use: 3/14 (21.4 %) Nasopharyngitis: 3/14 (21.4 %) Arthralgia: 3/14 (21.4 %) Pain in extremity: 3/14 (21.4 %) | Systemic lupus erythematosus: 2/16 (12.5 %) Arthralgia: 2/16 (12.5 %) Paraesthesia: 2/16 (12.5 %) Product use in unapproved indication: 2/16 (12.5 %) Antinuclear antibody positive: 2/16 (12.5 %) | Fatigue: 4/20 (20.0 %) Pain: 3/20 (15.0 %) Myalgia: 3/20 (15.0 %) Headache: 3/20 (15.0 %) Neuropathy peripheral: 3/20 (15.0 %) |
| **Reporter qualification** | Healthcare professional : 7/14 (50.0 %) Non healthcare professional : 7/14 (50.0 %) | Healthcare professional : 13/16 (81.25 %) Non healthcare professional : 3/16 (18.75 %) | Healthcare professional : 13/20 (65.0 %) Non healthcare professional : 7/20 (35.0 %) |
| **Indications** | Relapsing-remitting multiple sclerosis : 5/14 (35.7 %) Product used for unknown indication : 3/14 (21.4 %) Multiple sclerosis : 3/14 (21.4 %) Relapsing multiple sclerosis : 1/14 (7.1 %) Secondary progressive multiple sclerosis : 1/14 (7.1 %) | Product used for unknown indication : 3/16 (18.8 %) Asthma : 3/16 (18.8 %) Urticaria : 2/16 (12.5 %) Chronic urticaria : 2/16 (12.5 %) Unknown : 2/16 (12.5 %) | Unknown : 11/20 (55.0 %) Gastrointestinal stromal tumour : 3/20 (15.0 %) Product used for unknown indication : 1/20 (5.0 %) Gastrointestinal stromal tumor : 1/20 (5.0 %) Chronic myeloid leukaemia : 1/20 (5.0 %) |
| **Mean Time to Onset (std)** | 350 days (278 days) | 60 days (1 days) | 246 days (307 days) |

|  | **Leflunomide** | **Amitriptyline** | **Lorlatinib** |
| --- | --- | --- | --- |
| **Number of cases** | 11 | 13 | 4 |
| **IC025_RP_main** | 0.28 | 0.28 | 0.13 |
| **IC025_RP_comparator** | -0.11 | -0.47 | 0.11 |
| **IC025_RP_healthcare** | -0.38 | 0.01 | -1.43 |
| **IC025_RP_malefemale** | -0.56 | 0.12 | -0.21 |
| **IC025_RP-Broad_main** | 2.1 | 3.24 | -0.74 |
| **Sex (Female / Male / Unknown)** | 9 / 1 / 1 | 12 / 1 / 0 | 2 / 1 / 1 |
| **Mean age (SD) in years** | 52.43 (7.39) years | 51.0 (24.04) years | 58.33 (6.66) years |
| **Mean number of suspect/interacting drugs (SD)** | 6.11 (4.76) | 3.44 (1.13) | 2.33 (2.31) |
| **Withdrawn** | 1/11 (9.09 %) | 4/13 (30.77 %) | 0/4 (0.0 %) |
| **Outcome after drug withdrawal** | Not recovered: 1/1 (100.0 %) | Recovered: 1/4 (25.0 %) Recovered with sequelae: 1/4 (25.0 %) Not recovered: 2/4 (50.0 %) |  |
| **Country of primary source** | United States of America: 4/11 (36.4 %) Germany: 2/11 (18.2 %) France: 2/11 (18.2 %) Canada: 1/11 (9.1 %) Switzerland: 1/11 (9.1 %) | United Kingdom of Great Britain and Northern Ireland: 3/13 (23.1 %) Germany: 3/13 (23.1 %) Netherlands: 2/13 (15.4 %) Sweden: 1/13 (7.7 %) Iceland: 1/13 (7.7 %) | United States of America: 3/4 (75.0 %) France: 1/4 (25.0 %) |
| **Co-reported drugs** | Methotrexate: 6/ 11 (54.5 %) Methotrexate sodium: 6/ 11 (54.5 %) Etanercept: 4/ 11 (36.4 %) Methylprednisolone: 3/ 11 (27.3 %) Naproxen: 3/ 11 (27.3 %) | Sumatriptan: 2/ 13 (15.4 %) Zopiclone: 2/ 13 (15.4 %) Ibuprofen: 2/ 13 (15.4 %) Simvastatin: 1/ 13 (7.7 %) Esomeprazole: 1/ 13 (7.7 %) | Rosuvastatin: 2/ 4 (50.0 %) Glyceryl trinitrate: 1/ 4 (25.0 %) Tadalafil: 1/ 4 (25.0 %) Furosemide: 1/ 4 (25.0 %) Enoxaparin sodium: 1/ 4 (25.0 %) |
| **Co-reported reactions** | Arthralgia: 4/11 (36.4 %) Hypertension: 3/11 (27.3 %) Malaise: 3/11 (27.3 %) Drug ineffective: 3/11 (27.3 %) Diarrhoea: 2/11 (18.2 %) | Peripheral coldness: 1/13 (7.7 %) Non-cardiac chest pain: 1/13 (7.7 %) Gastritis: 1/13 (7.7 %) Fibromyalgia: 1/13 (7.7 %) Electrocardiogram repolarisation abnormality: 1/13 (7.7 %) | Neuropathy peripheral: 2/4 (50.0 %) Oedema: 1/4 (25.0 %) Oedema peripheral: 1/4 (25.0 %) Tendon injury: 1/4 (25.0 %) Diverticulum intestinal: 1/4 (25.0 %) |
| **Reporter qualification** | Healthcare professional : 6/11 (54.55 %) Non healthcare professional : 5/11 (45.45 %) | Healthcare professional : 10/13 (76.92 %) Non healthcare professional : 3/13 (23.08 %) | Healthcare professional : 2/4 (50.0 %) Non healthcare professional : 2/4 (50.0 %) |
| **Indications** | Product used for unknown indication : 4/11 (36.4 %) Rheumatoid arthritis : 3/11 (27.3 %) Unknown : 2/11 (18.2 %) Lung transplant : 1/11 (9.1 %) Psoriatic arthritis : 1/11 (9.1 %) | Unknown : 5/13 (38.5 %) Neuropathic pain : 2/13 (15.4 %) Migraine prophylaxis : 1/13 (7.7 %) Ill-defined disorder : 1/13 (7.7 %) Migraine : 1/13 (7.7 %) | Unknown : 1/4 (25.0 %) Non-small cell lung cancer stage IV : 1/4 (25.0 %) Metastases to central nervous system : 1/4 (25.0 %) Lung neoplasm malignant : 1/4 (25.0 %) Lung adenocarcinoma : 1/4 (25.0 %) |
| **Mean Time to Onset (std)** | 101 days (114 days) | 265 days (417 days) | nan |

|  | **Cinacalcet** | **Adalimumab** | **Lithium** |
| --- | --- | --- | --- |
| **Number of cases** | 14 | 144 | 11 |
| **IC025_RP_main** | 0.2 | 0.16 | 0.18 |
| **IC025_RP_comparator** | -0.95 | -0.26 | 0.88 |
| **IC025_RP_healthcare** | 0.52 | 0.21 | -0.78 |
| **IC025_RP_malefemale** | 0.6 | -0.32 | 0.24 |
| **IC025_RP-Broad_main** | 0.23 | -0.48 | 2.31 |
| **Sex (Female / Male / Unknown)** | 11 / 3 / 0 | 112 / 25 / 7 | 6 / 5 / 0 |
| **Mean age (SD) in years** | 54.91 (9.98) years | 52.41 (13.57) years | 45.3 (16.4) years |
| **Mean number of suspect/interacting drugs (SD)** | nan (nan) | 6.57 (6.25) | 5.5 (6.89) |
| **Withdrawn** | 0/14 (0.0 %) | 32/144 (22.22 %) | 1/11 (9.09 %) |
| **Outcome after drug withdrawal** |  | Recovered: 5/32 (15.62 %) Recovering: 2/32 (6.25 %) Not recovered: 6/32 (18.75 %) Evolution Unknown: 9/32 (28.12 %) | Recovering: 1/1 (100.0 %) |
| **Country of primary source** | Colombia: 13/14 (92.9 %) United States of America: 1/14 (7.1 %) | United States of America: 102/144 (70.8 %) United Kingdom of Great Britain and Northern Ireland: 10/144 (6.9 %) Germany: 7/144 (4.9 %) Canada: 5/144 (3.5 %) Colombia: 4/144 (2.8 %) | United States of America: 5/11 (45.5 %) France: 2/11 (18.2 %) Ireland: 1/11 (9.1 %) Denmark: 1/11 (9.1 %) Netherlands: 1/11 (9.1 %) |
| **Co-reported drugs** |  | Methotrexate sodium: 29/ 144 (20.1 %) Methotrexate: 29/ 144 (20.1 %) Prednisone: 17/ 144 (11.8 %) Folic acid: 16/ 144 (11.1 %) Hydroxychloroquine: 13/ 144 (9.0 %) | Valproate semisodium: 2/ 11 (18.2 %) Olanzapine: 2/ 11 (18.2 %) Levothyroxine sodium: 2/ 11 (18.2 %) Modafinil: 1/ 11 (9.1 %) Oxcarbazepine: 1/ 11 (9.1 %) |
| **Co-reported reactions** | Localised oedema: 1/14 (7.1 %) | Fatigue: 29/144 (20.1 %) Arthralgia: 24/144 (16.7 %) Pain: 23/144 (16.0 %) Systemic lupus erythematosus: 23/144 (16.0 %) Drug ineffective: 21/144 (14.6 %) | Peripheral coldness: 2/11 (18.2 %) Diarrhoea: 2/11 (18.2 %) Rash: 1/11 (9.1 %) Pneumonia: 1/11 (9.1 %) Malaise: 1/11 (9.1 %) |
| **Reporter qualification** | Healthcare professional : 14/14 (100.0 %) Non healthcare professional : 0/14 (0.0 %) | Healthcare professional : 49/144 (34.03 %) Non healthcare professional : 95/144 (65.97 %) | Healthcare professional : 6/11 (54.55 %) Non healthcare professional : 5/11 (45.45 %) |
| **Indications** | Primary hyperparathyroidism : 13/14 (92.9 %) Hypercalcaemia : 1/14 (7.1 %) | Rheumatoid arthritis : 61/144 (42.4 %) Unknown : 32/144 (22.2 %) Crohn's disease : 23/144 (16.0 %) Psoriatic arthropathy : 16/144 (11.1 %) Product used for unknown indication : 13/144 (9.0 %) | Unknown : 7/11 (63.6 %) Depression : 2/11 (18.2 %) Product used for unknown indication : 1/11 (9.1 %) Bipolar affective disorder : 1/11 (9.1 %) |
| **Mean Time to Onset (std)** | 388 days (166 days) | 602 days (647 days) | 5 days (9 days) |

|  | **Ofatumumab** | **Agalsidase beta** | **Peginterferon beta-1a** |
| --- | --- | --- | --- |
| **Number of cases** | 10 | 5 | 11 |
| **IC025_RP_main** | 0.31 | 0.08 | 0.1 |
| **IC025_RP_comparator** | -0.03 | 0.21 | -1.01 |
| **IC025_RP_healthcare** | -2.36 | -0.84 | -0.45 |
| **IC025_RP_malefemale** | 0.27 | -0.82 | -0.65 |
| **IC025_RP-Broad_main** | -0.7 | 0.05 | -0.93 |
| **Sex (Female / Male / Unknown)** | 7 / 2 / 1 | 4 / 1 / 0 | 11 / 0 / 0 |
| **Mean age (SD) in years** | 45.67 (5.32) years | 38.33 (4.62) years | 46.17 (17.17) years |
| **Mean number of suspect/interacting drugs (SD)** | 3.0 (3.46) | nan (nan) | 11.5 (10.47) |
| **Withdrawn** | 1/10 (10.0 %) | 0/5 (0.0 %) | 5/11 (45.45 %) |
| **Outcome after drug withdrawal** | Not recovered: 1/1 (100.0 %) |  | Not recovered: 4/5 (80.0 %) Evolution Unknown: 1/5 (20.0 %) |
| **Country of primary source** | United States of America: 8/10 (80.0 %) Austria: 1/10 (10.0 %) France: 1/10 (10.0 %) | United States of America: 3/5 (60.0 %) Finland: 1/5 (20.0 %) Colombia: 1/5 (20.0 %) | Germany: 3/11 (27.3 %) United States of America: 3/11 (27.3 %) Poland: 1/11 (9.1 %) United Kingdom of Great Britain and Northern Ireland: 1/11 (9.1 %) Spain: 1/11 (9.1 %) |
| **Co-reported drugs** | Modafinil: 1/ 10 (10.0 %) Atorvastatin calcium: 1/ 10 (10.0 %) Ascorbic acid: 1/ 10 (10.0 %) Vitamin d nos: 1/ 10 (10.0 %) Fish oil: 1/ 10 (10.0 %) |  | Vaccinium macrocarpon: 2/ 11 (18.2 %) Mometasone furoate: 2/ 11 (18.2 %) Loratadine: 2/ 11 (18.2 %) Macrogol 3350: 2/ 11 (18.2 %) Ferrous sulfate: 2/ 11 (18.2 %) |
| **Co-reported reactions** | Fatigue: 5/10 (50.0 %) Asthenia: 3/10 (30.0 %) Dizziness: 3/10 (30.0 %) Headache: 2/10 (20.0 %) Loss of consciousness: 2/10 (20.0 %) | Tinnitus: 2/5 (40.0 %) Deafness: 1/5 (20.0 %) Body temperature decreased: 1/5 (20.0 %) Temperature intolerance: 1/5 (20.0 %) Protein urine present: 1/5 (20.0 %) | Diverticular perforation: 2/11 (18.2 %) Nephropathy toxic: 2/11 (18.2 %) Hypotension: 2/11 (18.2 %) Pneumonia: 2/11 (18.2 %) Influenza like illness: 2/11 (18.2 %) |
| **Reporter qualification** | Healthcare professional : 2/10 (20.0 %) Non healthcare professional : 8/10 (80.0 %) | Healthcare professional : 3/5 (60.0 %) Non healthcare professional : 2/5 (40.0 %) | Healthcare professional : 4/11 (36.36 %) Non healthcare professional : 7/11 (63.64 %) |
| **Indications** | Multiple sclerosis : 5/10 (50.0 %) Unknown : 4/10 (40.0 %) Relapsing multiple sclerosis : 3/10 (30.0 %) Product used for unknown indication : 2/10 (20.0 %) | Fabry's disease : 2/5 (40.0 %) Unknown : 2/5 (40.0 %) Lipidosis : 1/5 (20.0 %) | Multiple sclerosis : 7/11 (63.6 %) Drug use for unknown indication : 2/11 (18.2 %) Unknown : 2/11 (18.2 %) MS : 1/11 (9.1 %) |
| **Mean Time to Onset (std)** | 476 days (543 days) | nan | 363 days (421 days) |

|  | **Sulfasalazine** | **Pramipexole** | **Pembrolizumab** |
| --- | --- | --- | --- |
| **Number of cases** | 10 | 6 | 20 |
| **IC025_RP_main** | 0.06 | 0.04 | 0.11 |
| **IC025_RP_comparator** | 0.49 | -1.13 | -0.06 |
| **IC025_RP_healthcare** | -0.19 | -1.03 | -0.04 |
| **IC025_RP_malefemale** | -1.17 | -0.13 | 0.95 |
| **IC025_RP-Broad_main** | 1.75 | 0.99 | -0.5 |
| **Sex (Female / Male / Unknown)** | 8 / 0 / 2 | 6 / 0 / 0 | 16 / 4 / 0 |
| **Mean age (SD) in years** | 41.71 (17.04) years | 62.0 (8.49) years | 63.47 (10.33) years |
| **Mean number of suspect/interacting drugs (SD)** | 2.44 (1.94) | 1.67 (0.58) | 6.0 (7.5) |
| **Withdrawn** | 4/10 (40.0 %) | 1/6 (16.67 %) | 9/20 (45.0 %) |
| **Outcome after drug withdrawal** | Recovered: 1/4 (25.0 %) Evolution Unknown: 3/4 (75.0 %) | Recovered: 1/1 (100.0 %) | Recovered: 2/9 (22.22 %) Recovering: 2/9 (22.22 %) Not recovered: 2/9 (22.22 %) Evolution Unknown: 3/9 (33.33 %) |
| **Country of primary source** | Germany: 2/10 (20.0 %) Canada: 2/10 (20.0 %) United States of America: 2/10 (20.0 %) Sweden: 1/10 (10.0 %) Netherlands: 1/10 (10.0 %) | France: 2/6 (33.3 %) United States of America: 2/6 (33.3 %) Germany: 1/6 (16.7 %) United Kingdom of Great Britain and Northern Ireland: 1/6 (16.7 %) | United States of America: 8/20 (40.0 %) France: 4/20 (20.0 %) Germany: 2/20 (10.0 %) Argentina: 2/20 (10.0 %) Australia: 1/20 (5.0 %) |
| **Co-reported drugs** | Methotrexate: 4/ 10 (40.0 %) Tofacitinib citrate: 2/ 10 (20.0 %) Infliximab: 2/ 10 (20.0 %) Methylprednisolone acetate: 1/ 10 (10.0 %) Secukinumab: 1/ 10 (10.0 %) | Rasagiline mesylate: 2/ 6 (33.3 %) Carbidopa Monohydrate;Entacapone;Levodopa: 1/ 6 (16.7 %) Selegiline: 1/ 6 (16.7 %) Amantadine: 1/ 6 (16.7 %) | Carboplatin: 7/ 20 (35.0 %) Acetylsalicylic acid: 3/ 20 (15.0 %) Acetylsalicylate lysine: 3/ 20 (15.0 %) Paracetamol: 3/ 20 (15.0 %) Pemetrexed: 3/ 20 (15.0 %) |
| **Co-reported reactions** | Hypersensitivity: 2/10 (20.0 %) Nausea: 2/10 (20.0 %) Chronic obstructive pulmonary disease: 1/10 (10.0 %) Malaise: 1/10 (10.0 %) Skin ulcer: 1/10 (10.0 %) | Peripheral coldness: 3/6 (50.0 %) Poor peripheral circulation: 2/6 (33.3 %) Constipation: 1/6 (16.7 %) Reduced facial expression: 1/6 (16.7 %) Depression: 1/6 (16.7 %) | Vasculitis: 2/20 (10.0 %) Tenosynovitis: 2/20 (10.0 %) Onycholysis: 2/20 (10.0 %) Pain: 2/20 (10.0 %) Neuropathy peripheral: 2/20 (10.0 %) |
| **Reporter qualification** | Healthcare professional : 8/10 (80.0 %) Non healthcare professional : 2/10 (20.0 %) | Healthcare professional : 3/6 (50.0 %) Non healthcare professional : 3/6 (50.0 %) | Healthcare professional : 18/20 (90.0 %) Non healthcare professional : 2/20 (10.0 %) |
| **Indications** | Product used for unknown indication : 4/10 (40.0 %) Rheumatoid arthritis : 3/10 (30.0 %) Unknown : 2/10 (20.0 %) Ulcerative proctitis : 1/10 (10.0 %) | Parkinson's disease : 2/6 (33.3 %) Unknown : 2/6 (33.3 %) Restless legs syndrome : 2/6 (33.3 %) | Unknown : 3/20 (15.0 %) Non-small cell lung cancer metastatic : 1/20 (5.0 %) Nasopharyngeal cancer : 1/20 (5.0 %) Metastatic bronchial carcinoma : 1/20 (5.0 %) Non-small cell lung cancer : 1/20 (5.0 %) |
| **Mean Time to Onset (std)** | 1279 days (1831 days) | nan | 38 days (35 days) |

|  | **Letrozole** | **Total** |
| --- | --- | --- |
| **Number of cases** | 9 | 4430 |
| **IC025_RP_main** | 0.02 |  |
| **IC025_RP_comparator** | -0.08 |  |
| **IC025_RP_healthcare** | -0.14 |  |
| **IC025_RP_malefemale** | 0.05 |  |
| **IC025_RP-Broad_main** | 0.13 |  |
| **Sex (Female / Male / Unknown)** | 8 / 1 / 0 | 3142 / 1100 / 188 |
| **Mean age (SD) in years** | 53.14 (17.49) years | 47.36 (19.0) years |
| **Mean number of suspect/interacting drugs (SD)** | 5.25 (5.68) | 3.49 (4.89) |
| **Withdrawn** | 6/9 (66.67 %) | 902/4430 (20.36 %) |
| **Outcome after drug withdrawal** | Recovered: 1/6 (16.67 %) Recovered with sequelae: 1/6 (16.67 %) Not recovered: 2/6 (33.33 %) Evolution Unknown: 2/6 (33.33 %) | Recovered: 215/902 (23.84 %) Recovering: 83/902 (9.2 %) Recovered with sequelae: 13/902 (1.44 %) Not recovered: 164/902 (18.18 %) Died: 3/902 (0.33 %) Evolution Unknown: 244/902 (27.05 %) |
| **Country of primary source** | United States of America: 4/9 (44.4 %) France: 2/9 (22.2 %) Germany: 2/9 (22.2 %) Brazil: 1/9 (11.1 %) | United States of America: 2005/4430 (45.3 %) France: 464/4430 (10.5 %) United Kingdom of Great Britain and Northern Ireland: 426/4430 (9.6 %) Germany: 305/4430 (6.9 %) Netherlands: 236/4430 (5.3 %) |
| **Co-reported drugs** | Palbociclib: 2/ 9 (22.2 %) Ibandronate sodium: 1/ 9 (11.1 %) Allopurinol: 1/ 9 (11.1 %) Lercanidipine hydrochloride: 1/ 9 (11.1 %) Pantoprazole sodium sesquihydrate: 1/ 9 (11.1 %) | Methylphenidate hydrochloride: 254/ 4430 (5.7 %) Methylphenidate: 254/ 4430 (5.7 %) Levothyroxine: 235/ 4430 (5.3 %) Levothyroxine sodium: 235/ 4430 (5.3 %) Acetylsalicylic acid: 226/ 4430 (5.1 %) |
| **Co-reported reactions** | Arthralgia: 3/9 (33.3 %) Vulvovaginal dryness: 2/9 (22.2 %) Alopecia: 2/9 (22.2 %) Hot flush: 2/9 (22.2 %) Systemic scleroderma: 2/9 (22.2 %) | Fatigue: 407/4430 (9.2 %) Arthralgia: 394/4430 (8.9 %) Pain: 285/4430 (6.4 %) Pain in extremity: 275/4430 (6.2 %) Headache: 260/4430 (5.9 %) |
| **Reporter qualification** | Healthcare professional : 7/9 (77.78 %) Non healthcare professional : 2/9 (22.22 %) | Healthcare professional : 2601/4430 (58.71 %) Non healthcare professional : 1829/4430 (41.29 %) |
| **Indications** | Breast cancer : 5/9 (55.6 %) Unknown : 2/9 (22.2 %) Ovarian cancer : 1/9 (11.1 %) Breast carcinoma : 1/9 (11.1 %) Body height below normal : 1/9 (11.1 %) | Unknown : 878/4430 (19.8 %) Product used for unknown indication : 268/4430 (6.0 %) Rheumatoid arthritis : 262/4430 (5.9 %) Migraine : 182/4430 (4.1 %) ADHD : 152/4430 (3.4 %) |
| **Mean Time to Onset (std)** | 185 days (131 days) | 610 days (1031 days) |

# Supplementary Table 4. Preferred terms associated with an event-event disproportionate reporting using narrow definition of Raynaud's phenomenon among each drug class

| **Prefered Terms** | **Number of cases** | **IC_025_** | **Drug class** |
| --- | --- | --- | --- |
| Amnesia | 5 | 0.02 | Beta blockers (BB) |
| Oedema peripheral | 10 | 0.26 | Beta blockers (BB) |
| Paraesthesia | 13 | 0.69 | Beta blockers (BB) |
| Breast pain | 3 | 0.25 | Beta blockers (BB) |
| Peripheral coldness | 23 | 2.55 | Beta blockers (BB) |
| Anxiety | 9 | 0.25 | Beta blockers (BB) |
| Depression | 12 | 0.56 | Beta blockers (BB) |
| Hypoaesthesia | 18 | 2.02 | Beta blockers (BB) |
| Skin discolouration | 15 | 2.87 | Beta blockers (BB) |
| Arthralgia | 13 | 0.88 | Beta blockers (BB) |
| Pain in extremity | 14 | 1.29 | Beta blockers (BB) |
| Skin ulcer | 4 | 0.87 | Beta blockers (BB) |
| Extremity necrosis | 3 | 0.67 | Beta blockers (BB) |
| Dry eye | 8 | 0.95 | Beta blockers (BB) |
| Peripheral swelling | 8 | 0.75 | Beta blockers (BB) |
| Sjogren's syndrome | 3 | 0.62 | Beta blockers (BB) |
| Scleroderma | 4 | 1.30 | Beta blockers (BB) |
| Osteoarthritis | 4 | 0.54 | Beta blockers (BB) |
| Osteoporosis | 3 | 0.17 | Beta blockers (BB) |
| Maternal exposure during pregnancy | 7 | 0.87 | Beta blockers (BB) |
| Condition aggravated | 10 | 0.38 | Beta blockers (BB) |
| Fibromyalgia | 4 | 0.93 | Beta blockers (BB) |
| Gastrooesophageal reflux disease | 7 | 0.77 | Beta blockers (BB) |
| Weight decreased | 8 | 0.20 | Beta blockers (BB) |
| Nipple pain | 5 | 1.67 | Beta blockers (BB) |
| Depressed mood | 4 | 0.19 | Beta blockers (BB) |
| Pallor | 4 | 0.36 | Beta blockers (BB) |
| Herpes zoster | 3 | 0.15 | Beta blockers (BB) |
| Disturbance in attention | 4 | 0.13 | Beta blockers (BB) |
| Neuralgia | 6 | 1.90 | Beta blockers (BB) |
| Ventricular extrasystoles | 4 | 0.18 | Beta blockers (BB) |
| Memory impairment | 6 | 0.27 | Beta blockers (BB) |
| Intermittent claudication | 3 | 0.29 | Beta blockers (BB) |
| Prinzmetal angina | 3 | 0.54 | Beta blockers (BB) |
| Feeling cold | 7 | 1.74 | Antimigraine drugs (AM) |
| Chillblains | 3 | 0.70 | Antimigraine drugs (AM) |
| Condition aggravated | 17 | 1.80 | Antimigraine drugs (AM) |
| Skin discolouration | 18 | 3.65 | Antimigraine drugs (AM) |
| Alopecia | 13 | 0.23 | Antimigraine drugs (AM) |
| Peripheral coldness | 23 | 4.06 | Antimigraine drugs (AM) |
| Paraesthesia | 18 | 0.55 | Antimigraine drugs (AM) |
| Pain in extremity | 11 | 1.17 | Antimigraine drugs (AM) |
| Hypoaesthesia | 14 | 1.14 | Antimigraine drugs (AM) |
| Pallor | 4 | 0.83 | Antimigraine drugs (AM) |
| Weight increased | 13 | 0.59 | Antimigraine drugs (AM) |
| Arthralgia | 16 | 1.15 | Antimigraine drugs (AM) |
| Poor peripheral circulation | 6 | 2.21 | Antimigraine drugs (AM) |
| Hyperhidrosis | 7 | 0.04 | Antimigraine drugs (AM) |
| Systemic lupus erythematosus | 3 | 0.33 | Antimigraine drugs (AM) |
| Abdominal distension | 5 | 0.04 | Antimigraine drugs (AM) |
| Anxiety | 11 | 0.20 | Antimigraine drugs (AM) |
| Extremity necrosis | 3 | 0.73 | Antimigraine drugs (AM) |
| Burning sensation | 5 | 0.08 | Antimigraine drugs (AM) |
| Cyanosis | 3 | 0.27 | Antimigraine drugs (AM) |
| Muscle spasms | 7 | 0.04 | Antimigraine drugs (AM) |
| Stress | 5 | 0.46 | Antimigraine drugs (AM) |
| Chills | 5 | 0.37 | Antimigraine drugs (AM) |
| Ventricular tachycardia | 3 | 0.10 | Antimigraine drugs (AM) |
| Scleroderma | 4 | 1.32 | Amfetamine-like (drugs) (AL) |
| Chillblains | 7 | 2.34 | Amfetamine-like (drugs) (AL) |
| Paraesthesia | 11 | 0.53 | Amfetamine-like (drugs) (AL) |
| Peripheral coldness | 19 | 2.45 | Amfetamine-like (drugs) (AL) |
| Peripheral swelling | 9 | 1.60 | Amfetamine-like (drugs) (AL) |
| Bruxism | 10 | 1.21 | Amfetamine-like (drugs) (AL) |
| Livedo reticularis | 5 | 1.53 | Amfetamine-like (drugs) (AL) |
| Feeling cold | 6 | 1.17 | Amfetamine-like (drugs) (AL) |
| Skin discolouration | 22 | 3.24 | Amfetamine-like (drugs) (AL) |
| Pain in extremity | 17 | 1.95 | Amfetamine-like (drugs) (AL) |
| Hypoaesthesia | 17 | 1.45 | Amfetamine-like (drugs) (AL) |
| Systemic lupus erythematosus | 3 | 0.38 | Amfetamine-like (drugs) (AL) |
| Erythromelalgia | 3 | 0.62 | Amfetamine-like (drugs) (AL) |
| Vasculitis | 4 | 0.91 | Amfetamine-like (drugs) (AL) |
| Cyanosis | 15 | 2.83 | Amfetamine-like (drugs) (AL) |
| Arthralgia | 8 | 0.26 | Amfetamine-like (drugs) (AL) |
| Pallor | 4 | 0.04 | Amfetamine-like (drugs) (AL) |
| Erythema | 13 | 1.64 | Amfetamine-like (drugs) (AL) |
| Peripheral vascular disorder | 3 | 0.36 | Amfetamine-like (drugs) (AL) |
| Vasospasm | 3 | 0.47 | Amfetamine-like (drugs) (AL) |
| Carpal tunnel syndrome | 4 | 1.08 | Amfetamine-like (drugs) (AL) |
| Antinuclear antibody positive | 4 | 1.05 | Amfetamine-like (drugs) (AL) |
| Angiopathy | 4 | 1.17 | Amfetamine-like (drugs) (AL) |
| Skin discolouration | 11 | 2.89 | TKI-Onc |
| Pain | 18 | 0.51 | TKI-Onc |
| Diarrhoea | 11 | 0.23 | TKI-Onc |
| Myalgia | 11 | 1.57 | TKI-Onc |
| Headache | 13 | 0.44 | TKI-Onc |
| Dyspepsia | 7 | 1.18 | TKI-Onc |
| Neuropathy peripheral | 11 | 2.56 | TKI-Onc |
| Oedema | 5 | 0.46 | TKI-Onc |
| Pain in extremity | 11 | 0.55 | TKI-Onc |
| Weight decreased | 12 | 2.02 | TKI-Onc |
| Peripheral swelling | 9 | 0.68 | TKI-Onc |
| Back pain | 12 | 1.59 | TKI-Onc |
| Anxiety | 7 | 1.07 | TKI-Onc |
| Malaise | 9 | 0.05 | TKI-Onc |
| Insomnia | 6 | 0.57 | TKI-Onc |
| Fatigue | 24 | 1.36 | TKI-Onc |
| Hypoaesthesia | 15 | 2.77 | TKI-Onc |
| Feeling abnormal | 7 | 0.72 | TKI-Onc |
| Dry skin | 5 | 0.79 | TKI-Onc |
| Paraesthesia | 10 | 2.16 | TKI-Onc |
| Cyanosis | 3 | 0.70 | TKI-Onc |
| Joint swelling | 10 | 1.14 | TKI-Onc |
| Oedema peripheral | 6 | 0.71 | TKI-Onc |
| Musculoskeletal stiffness | 13 | 1.77 | TKI-Onc |
| Therapeutic response unexpected | 4 | 0.27 | TKI-Onc |
| Dry eye | 13 | 3.03 | TKI-Onc |
| Peripheral coldness | 7 | 2.43 | TKI-Onc |
| Dry mouth | 16 | 3.27 | TKI-Onc |
| Chest pain | 6 | 0.80 | TKI-Onc |
| Neoplasm | 5 | 1.55 | TKI-Onc |
| Sjogren's syndrome | 5 | 1.74 | TKI-Onc |
| Hypotension | 4 | 0.76 | TKI-Onc |
| Brain fog | 3 | 0.40 | TKI-Onc |
| White blood cell count decreased | 5 | 0.44 | TKI-Onc |
| Musculoskeletal discomfort | 4 | 0.95 | TKI-Onc |
| Arthralgia | 25 | 1.57 | TKI-Onc |
| Dysuria | 3 | 0.36 | TKI-Onc |
| Peripheral arterial occlusive disease | 3 | 0.13 | TKI-Onc |
| Asthenia | 8 | 0.67 | TKI-Onc |
| Constipation | 7 | 0.86 | TKI-Onc |
| Hot flush | 3 | 0.21 | TKI-Onc |
| Condition aggravated | 12 | 0.20 | TKI-Onc |
| Osteoarthritis | 5 | 1.12 | TKI-Onc |
| Arthritis | 6 | 0.68 | TKI-Onc |
| Onychoclasis | 3 | 0.62 | TKI-Onc |
| Onychomycosis | 4 | 1.27 | TKI-Onc |
| Nail discolouration | 3 | 0.65 | TKI-Onc |
| Finger deformity | 3 | 0.42 | TKI-Onc |
| Dyspnoea | 9 | 0.71 | TKI-Onc |
| Nail infection | 3 | 0.70 | TKI-Onc |
| Temperature intolerance | 4 | 1.27 | TKI-Onc |
| Neuralgia | 4 | 1.01 | TKI-Onc |
| Fibromyalgia | 4 | 0.87 | TKI-Onc |
| Night sweats | 5 | 1.39 | TKI-Onc |
| Increased tendency to bruise | 4 | 1.17 | TKI-Onc |
| Sleep disorder | 5 | 1.13 | TKI-Onc |
| Pallor | 3 | 0.49 | TKI-Onc |

Supplementary Figure 1. Sankey diagram representing target of each drug per class using DrugBank database

L = Legend, A = Beta-blockers, B = Antimigraine drugs, C = Amfetamine-like drugs, D= Interferons, E = Dopaminergic agonists, F = Tyrosine Kinase inhibitors, G = Contraceptive hormones, H = Antineoplastic agents, I = Antidepressants, J = Immunosuppressants, K = Others


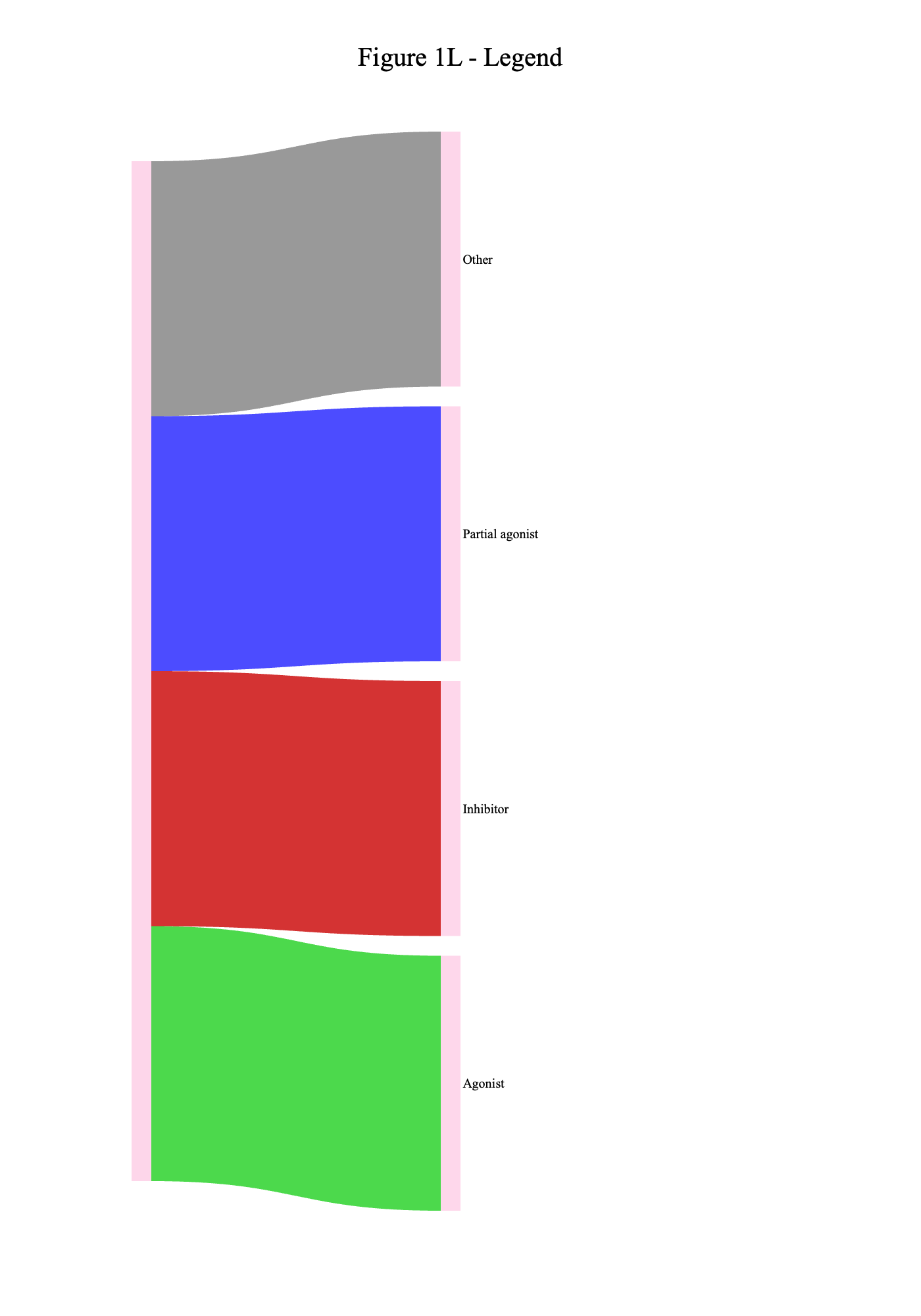


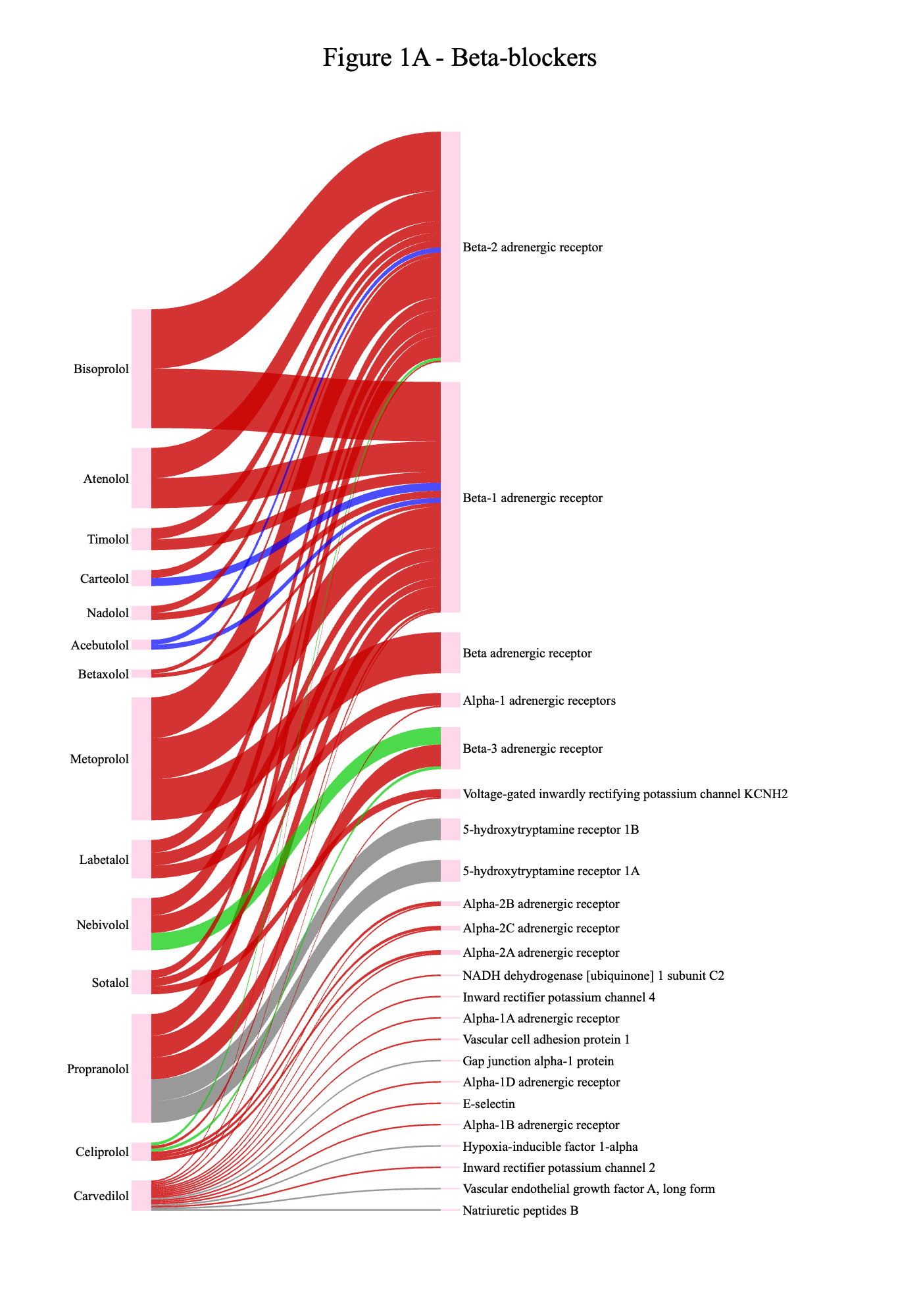


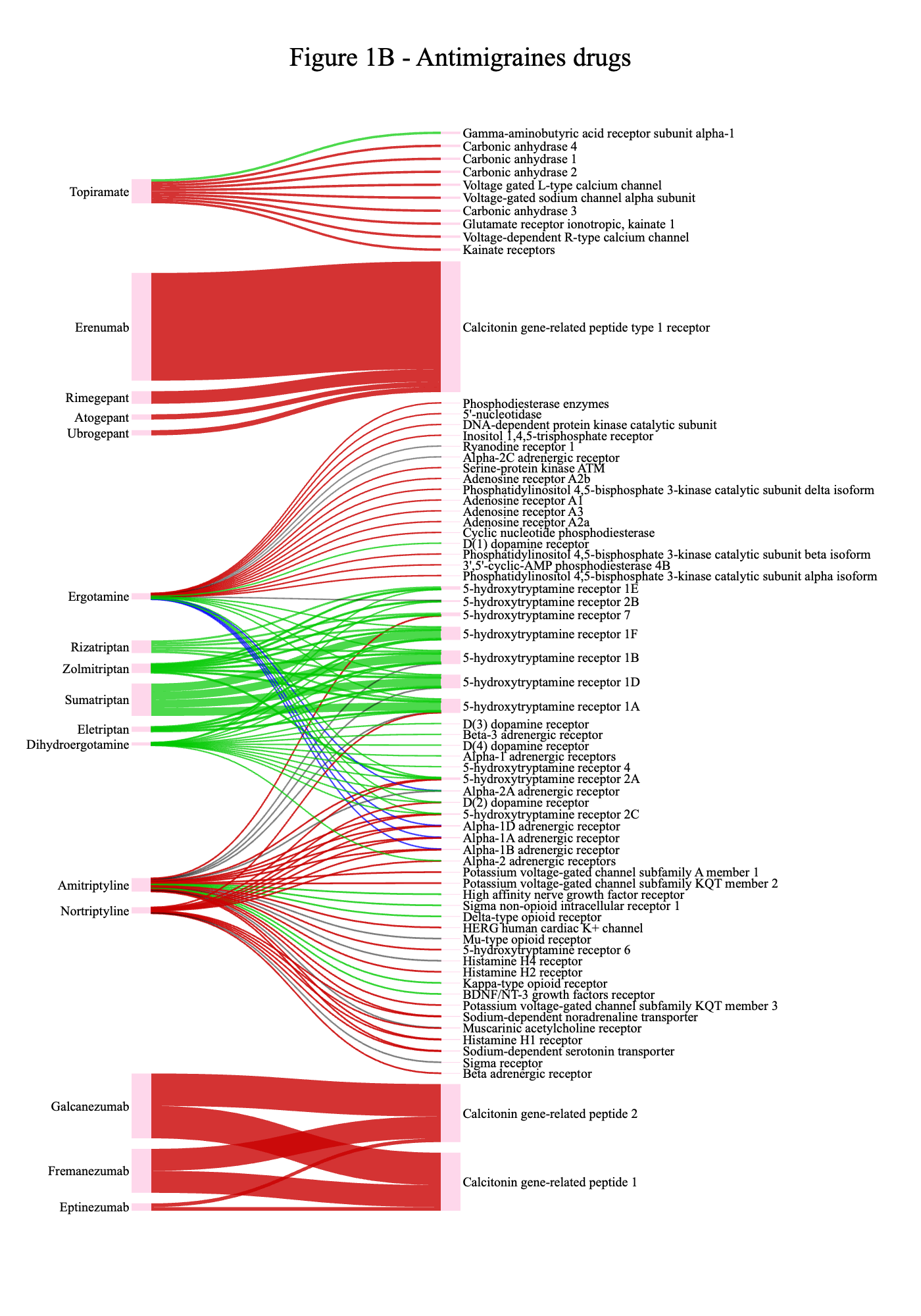


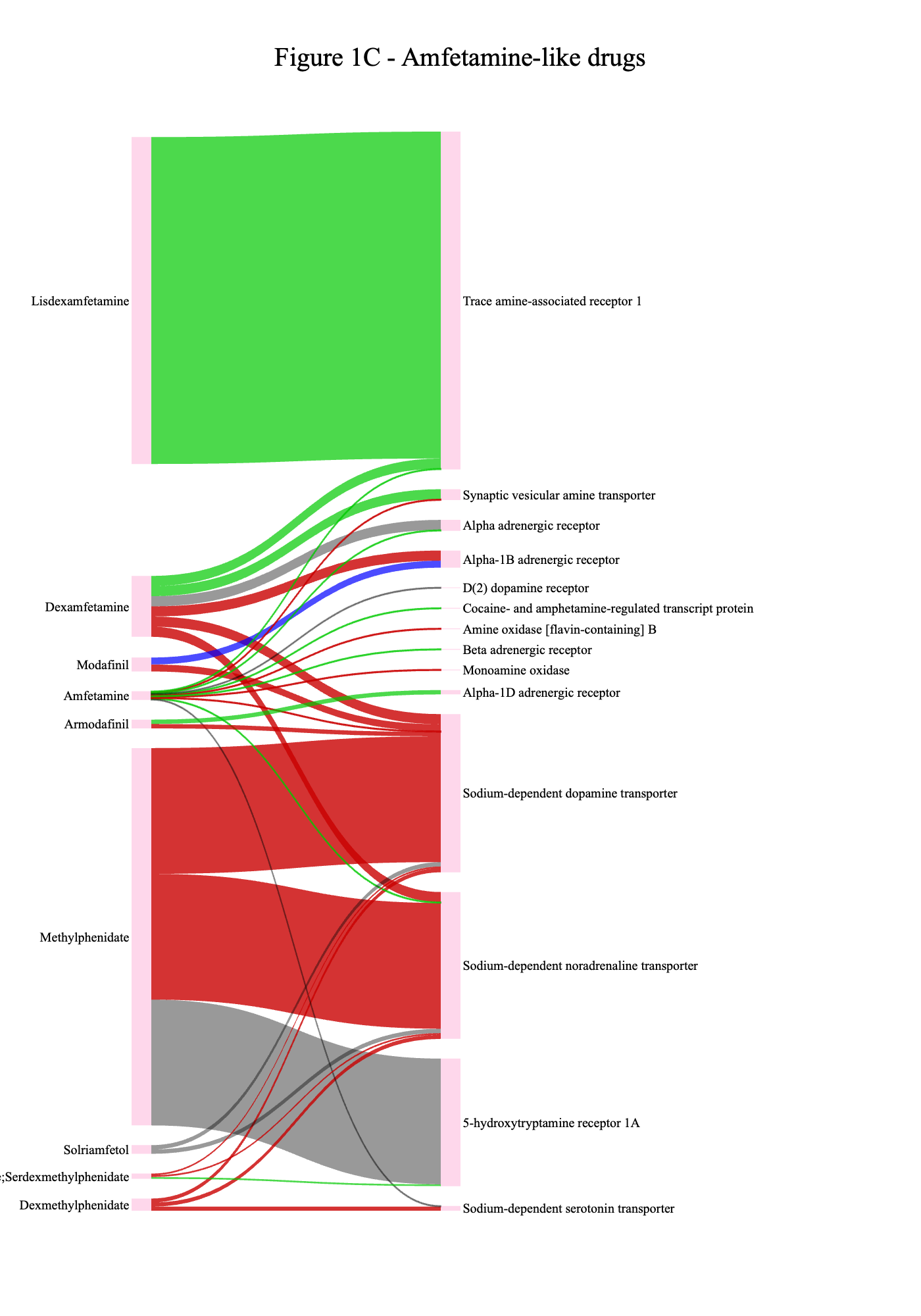


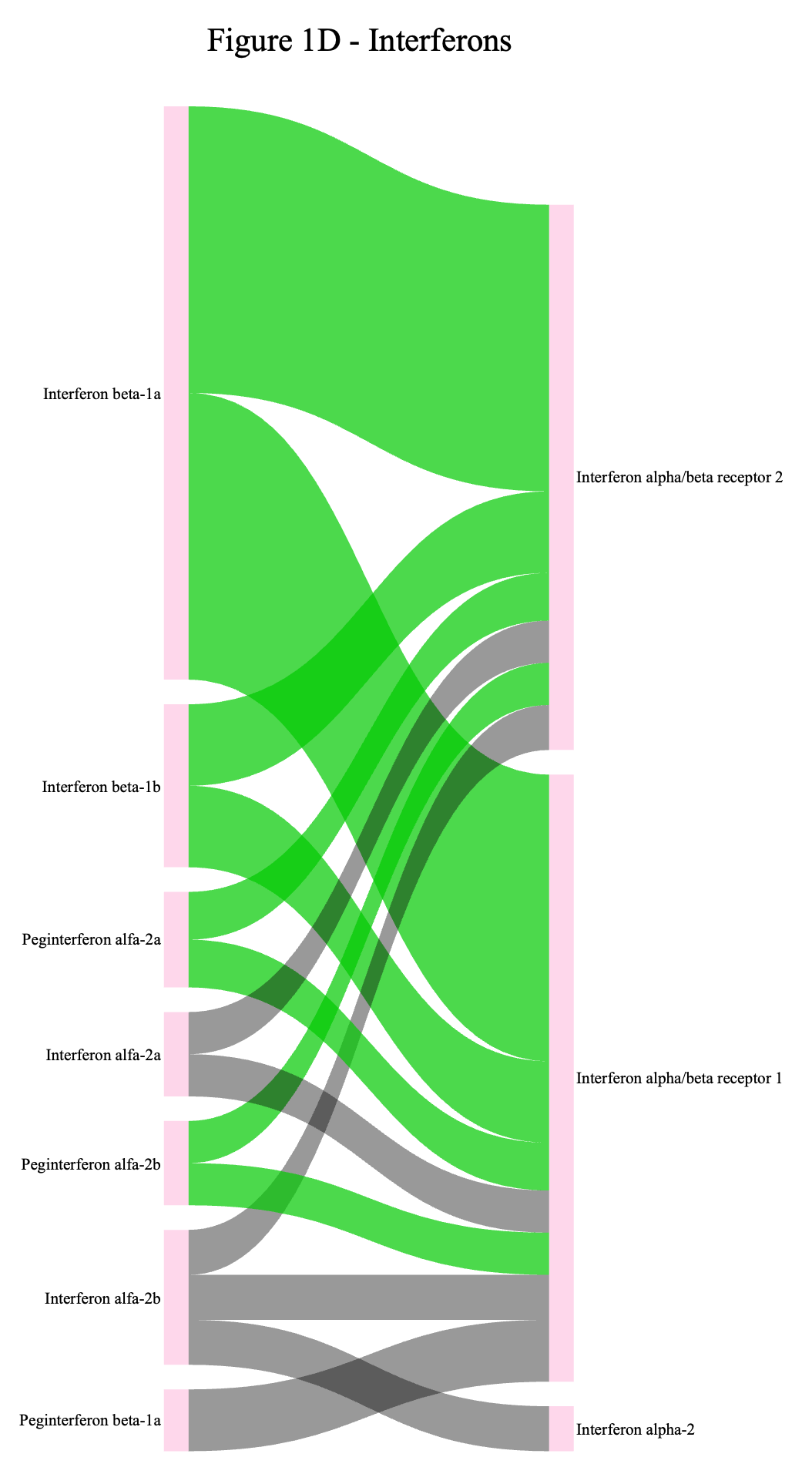


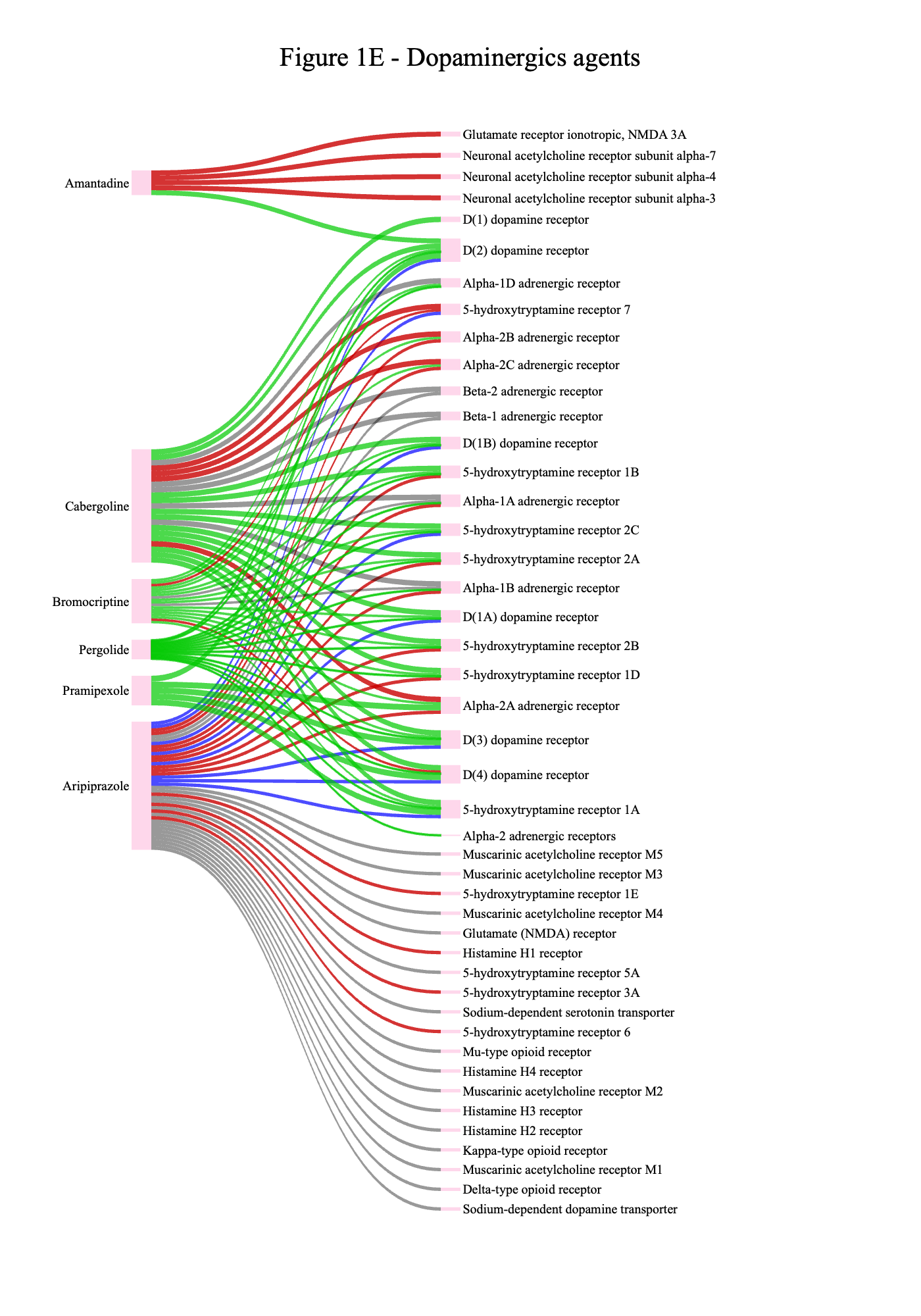


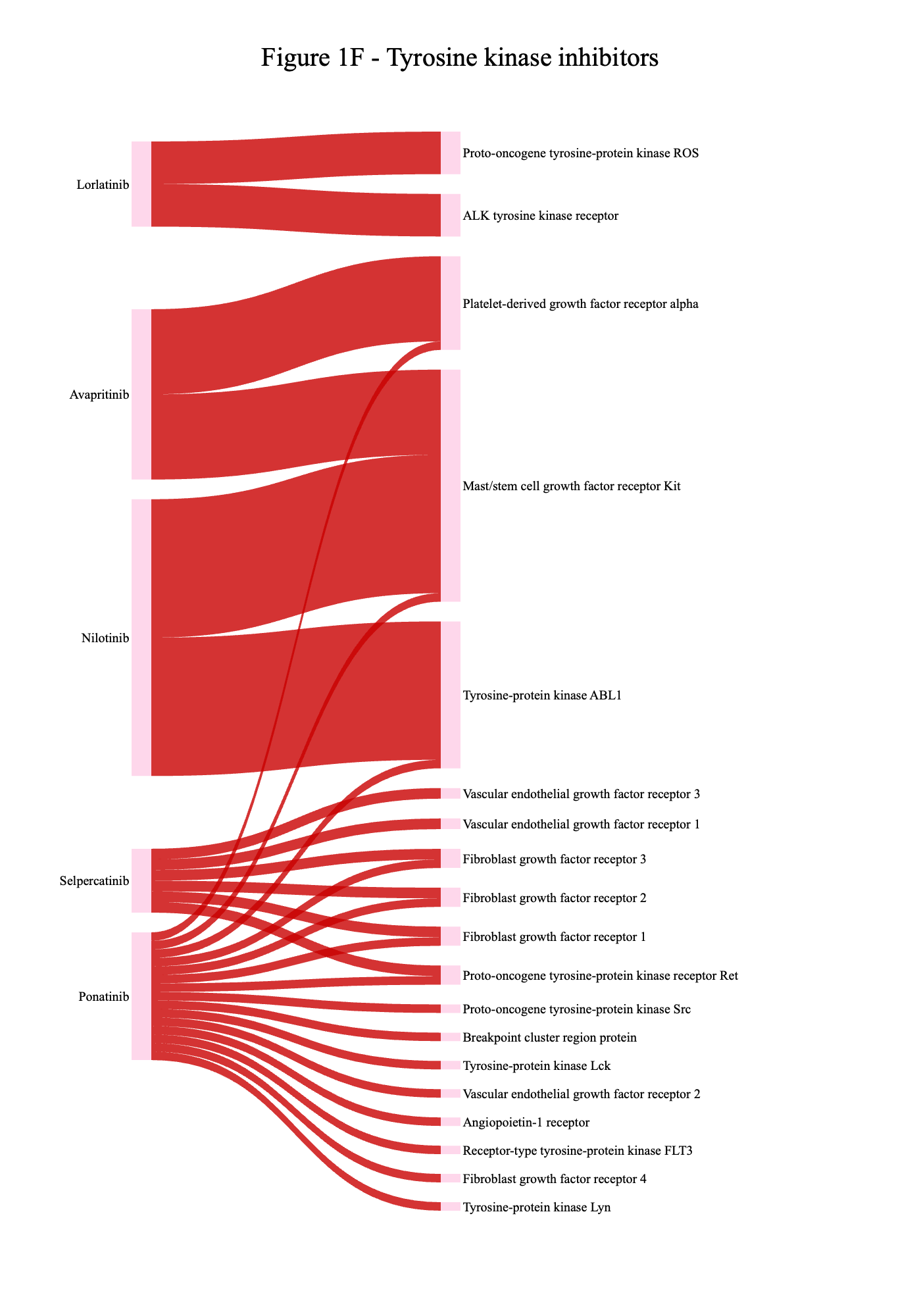


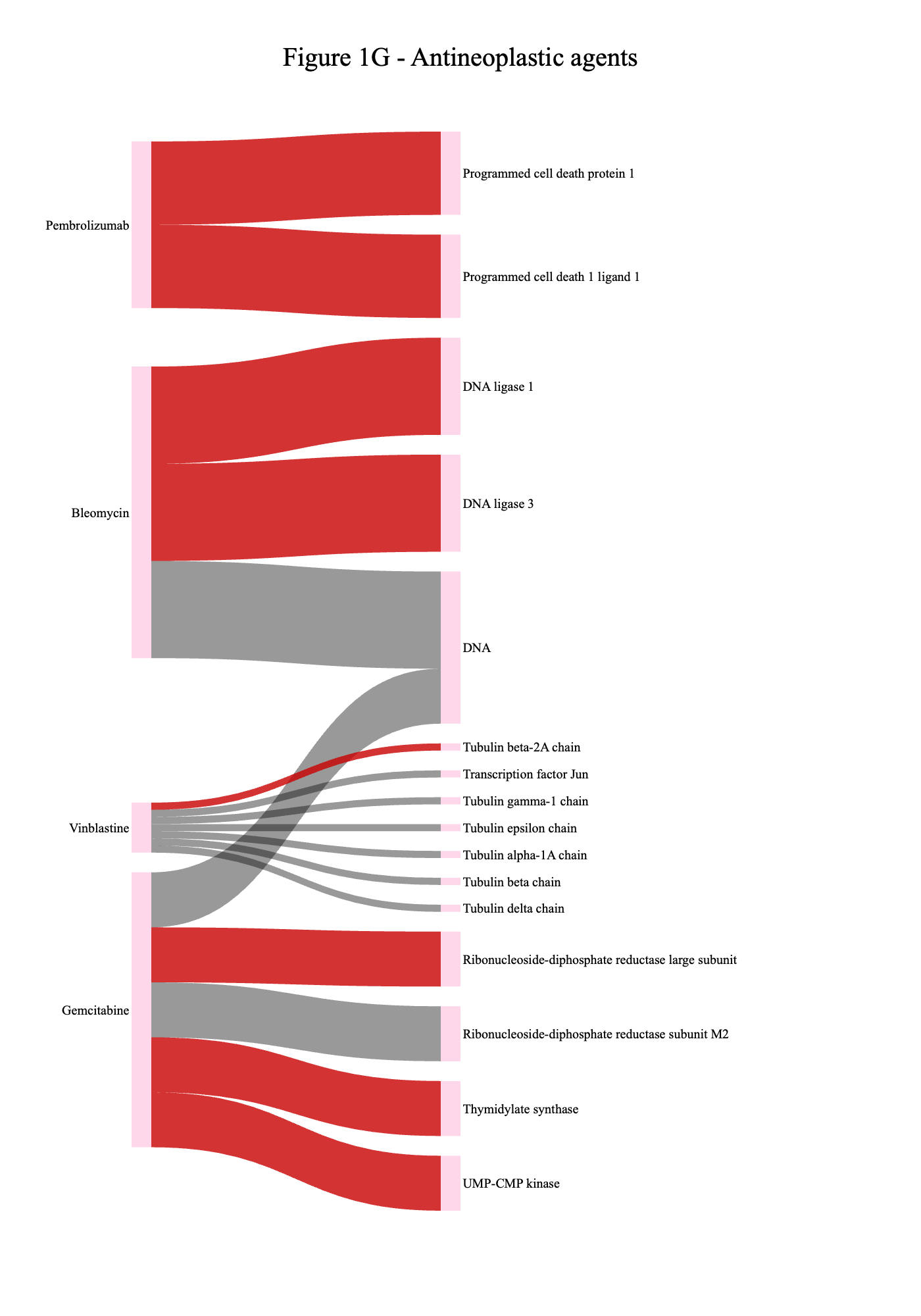


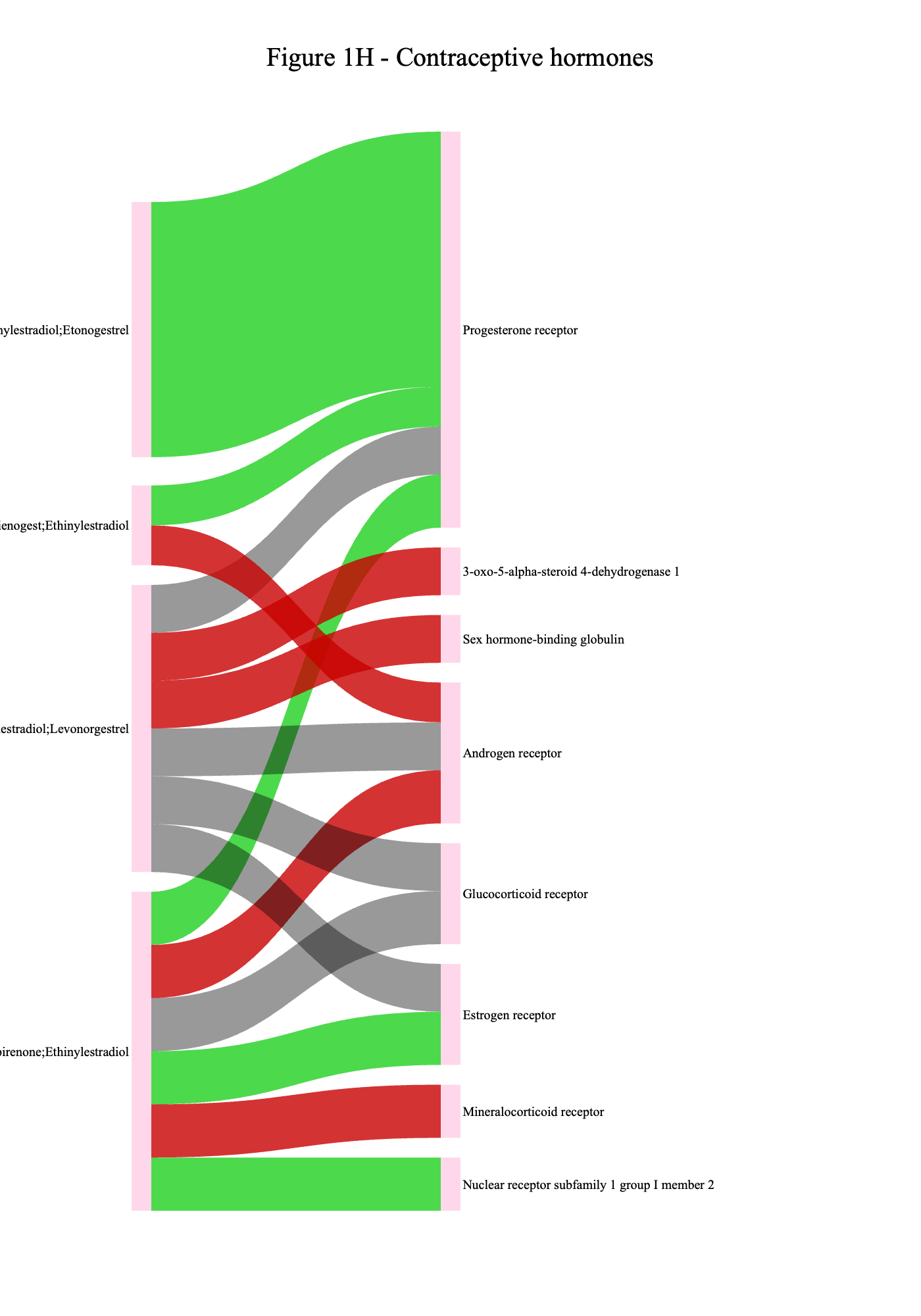


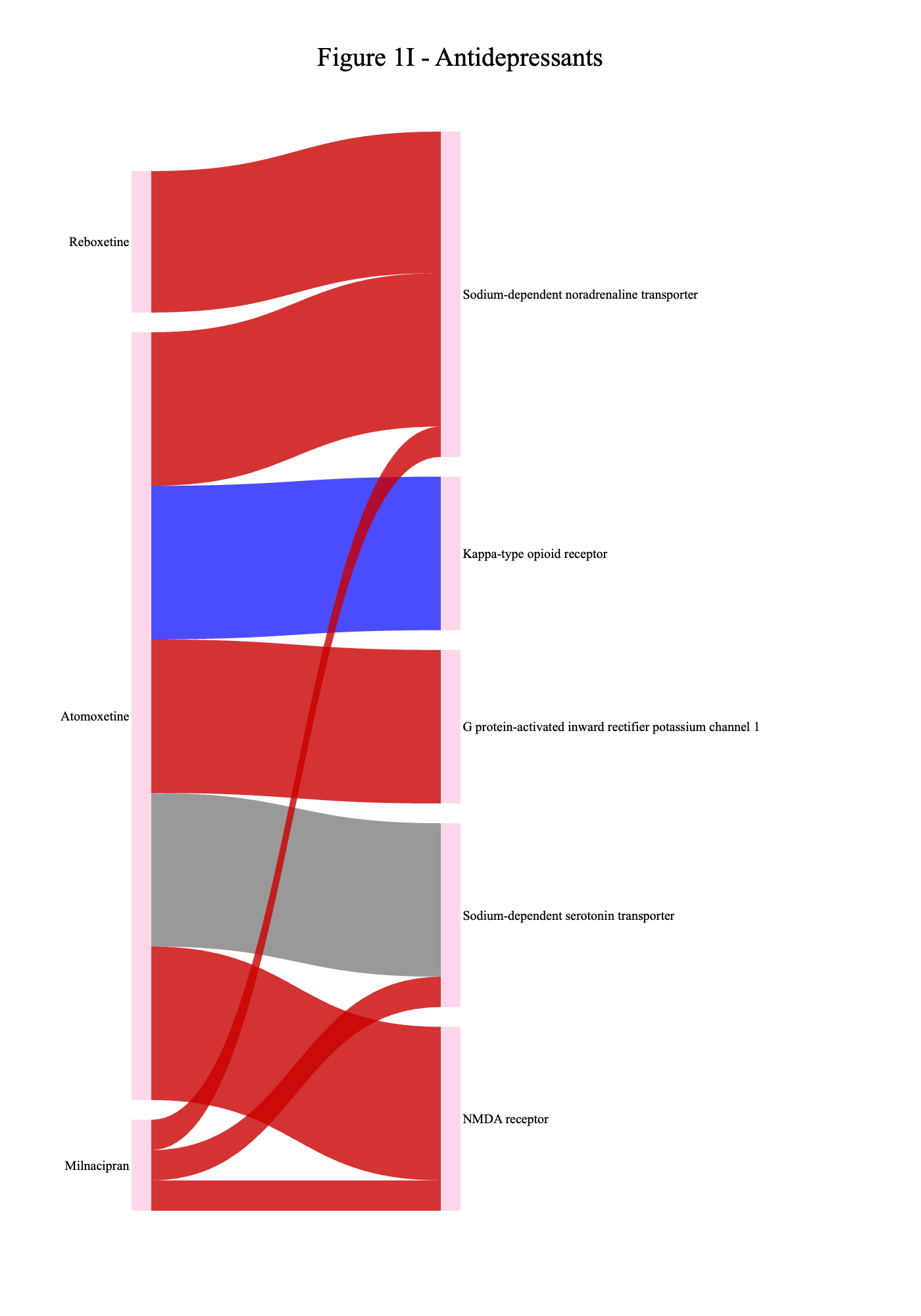


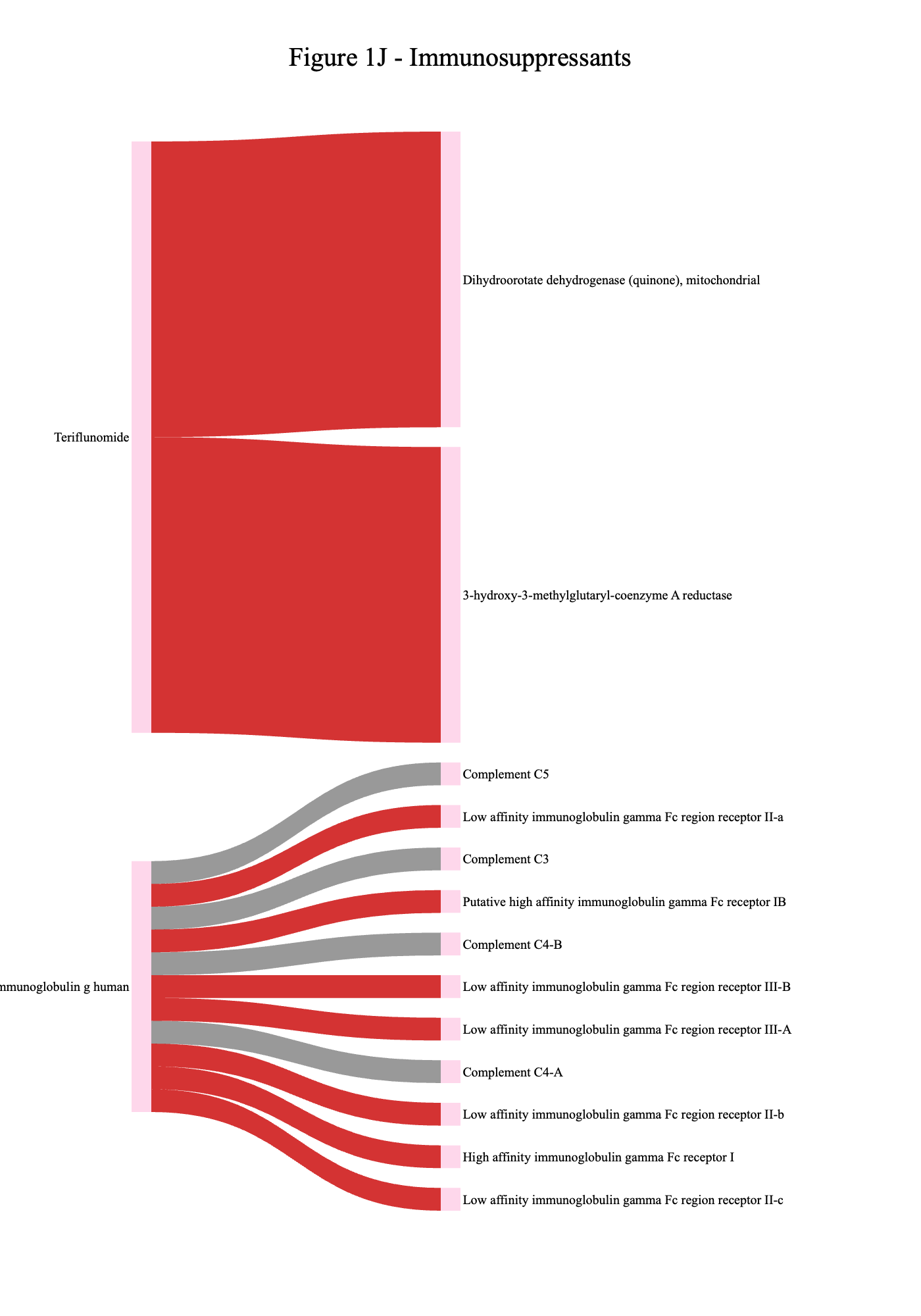


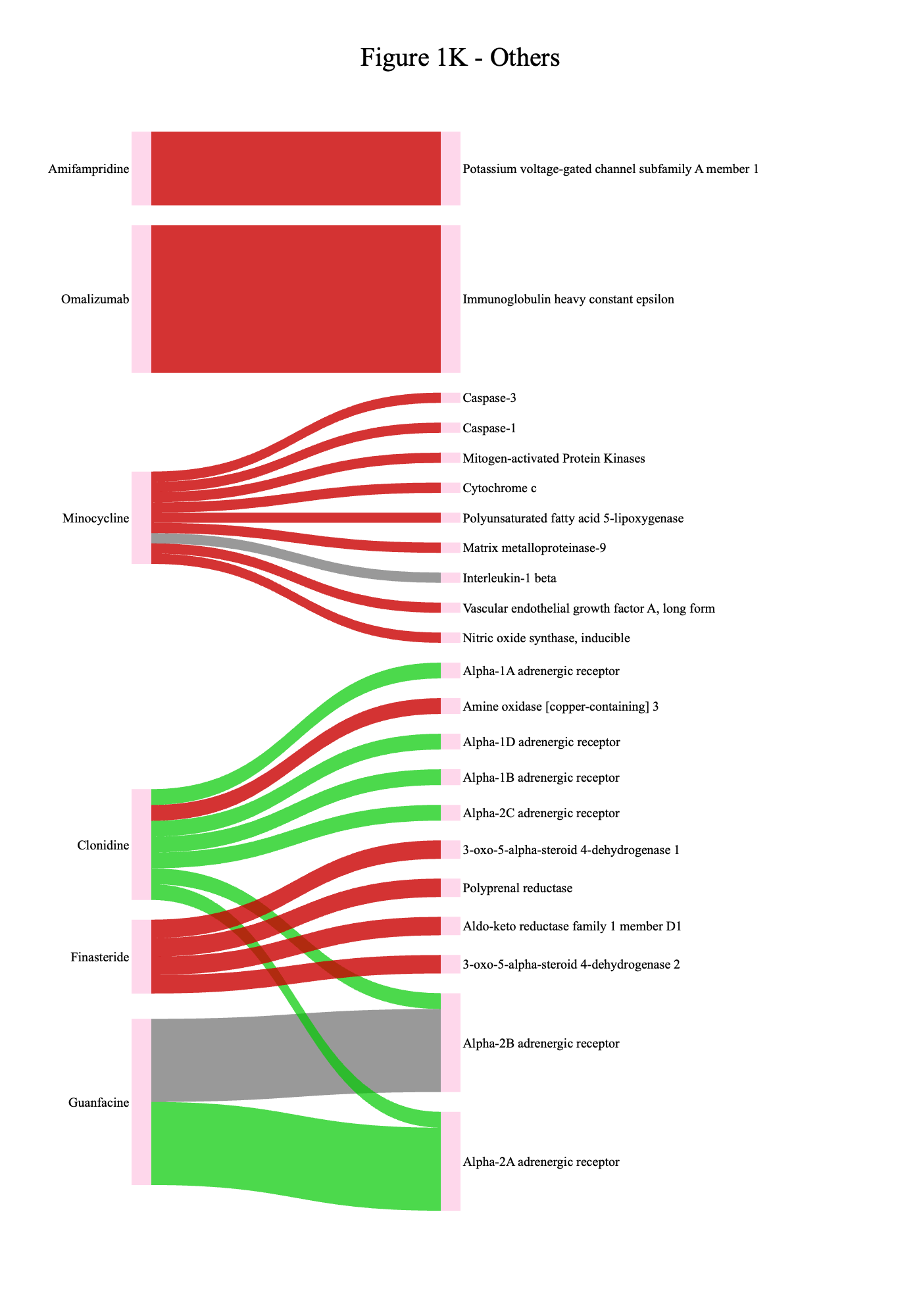


# Supplementary Figure 2. Network clustering using PPMI matrix and Ising model for PTs associated with beta adrenoreceptor blockers


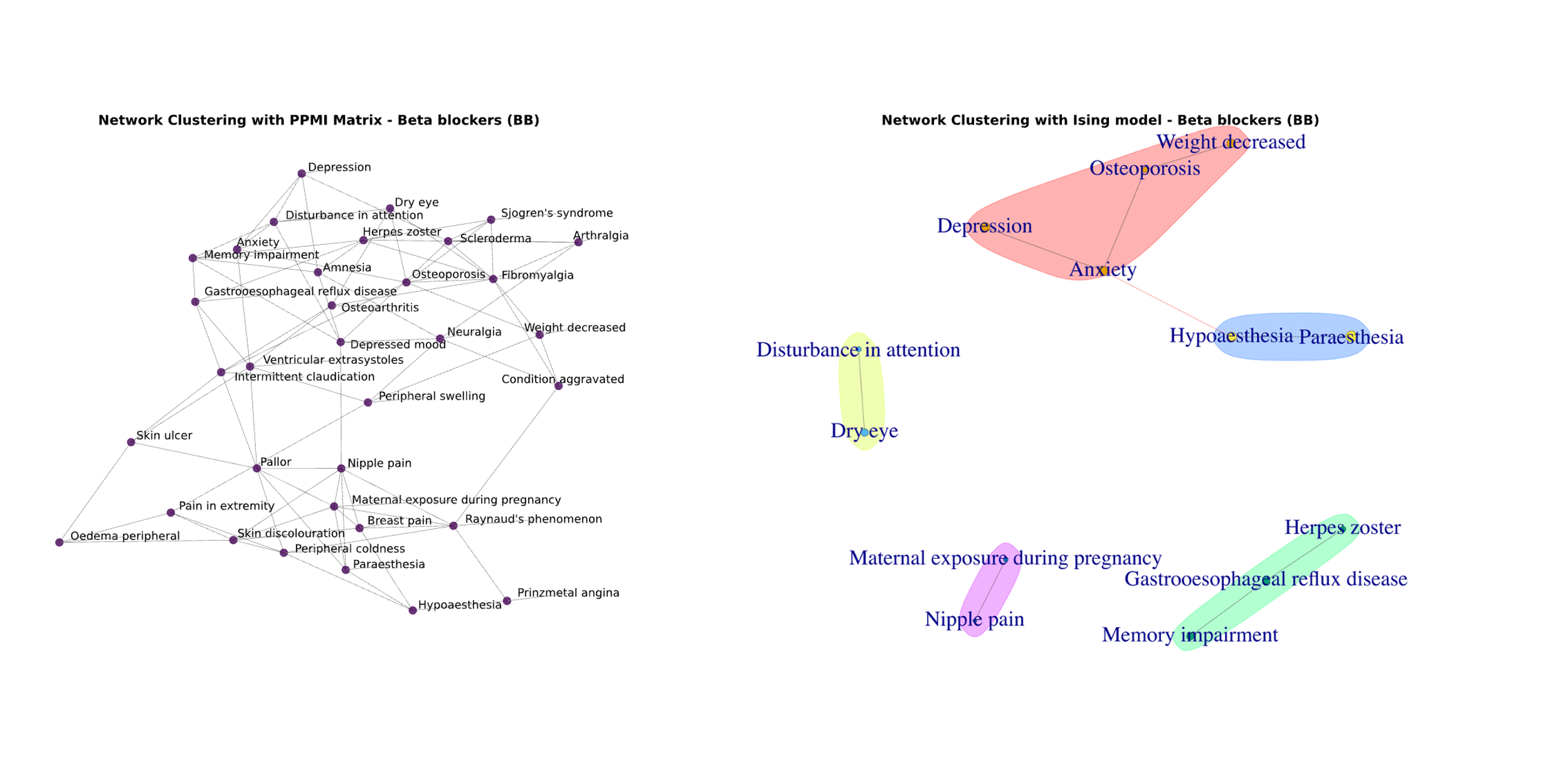


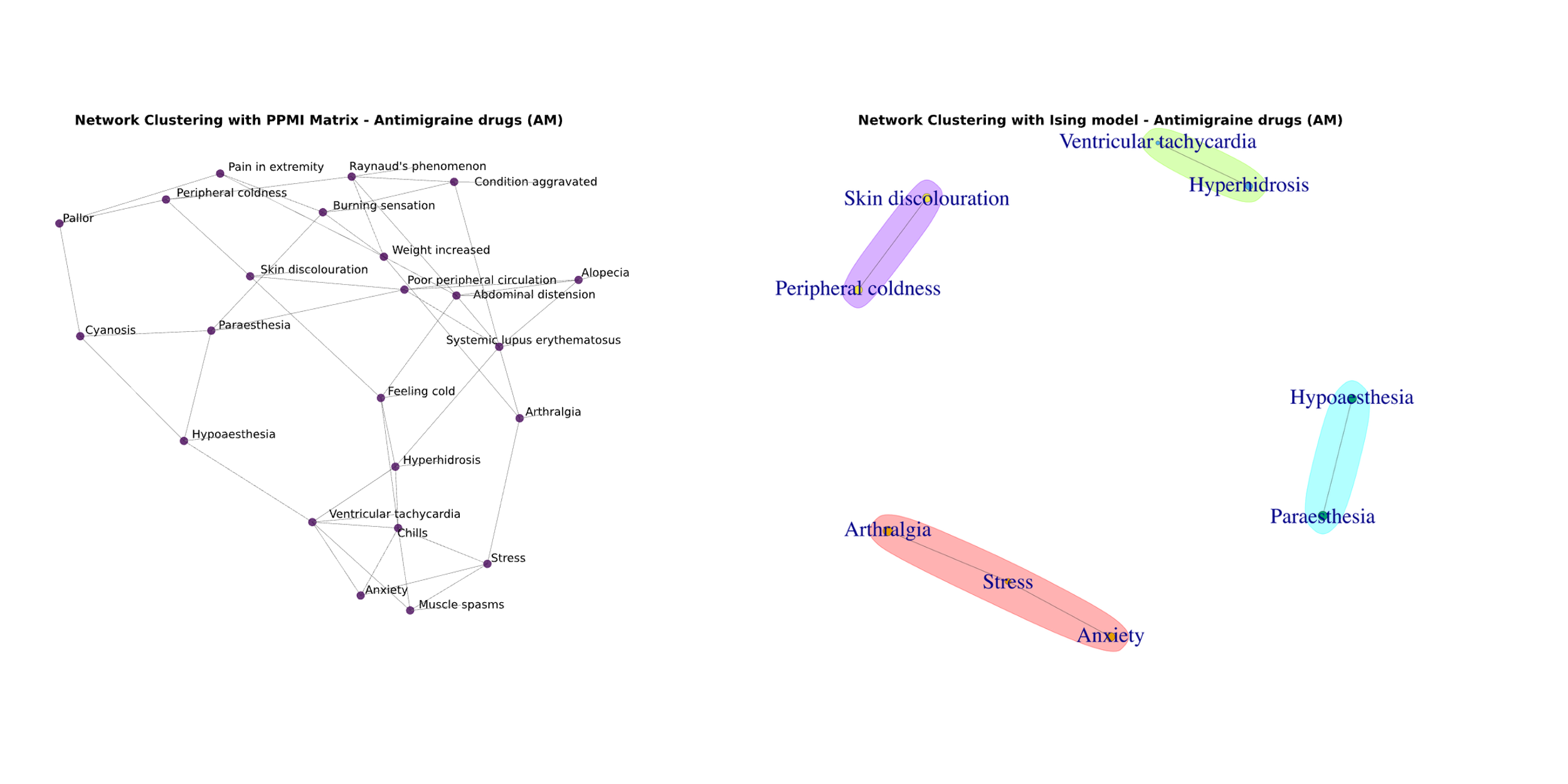


Figure 3 - Network clustering using PPMI matrix and Ising model for PTs associate with antimigraine drugs (AM)


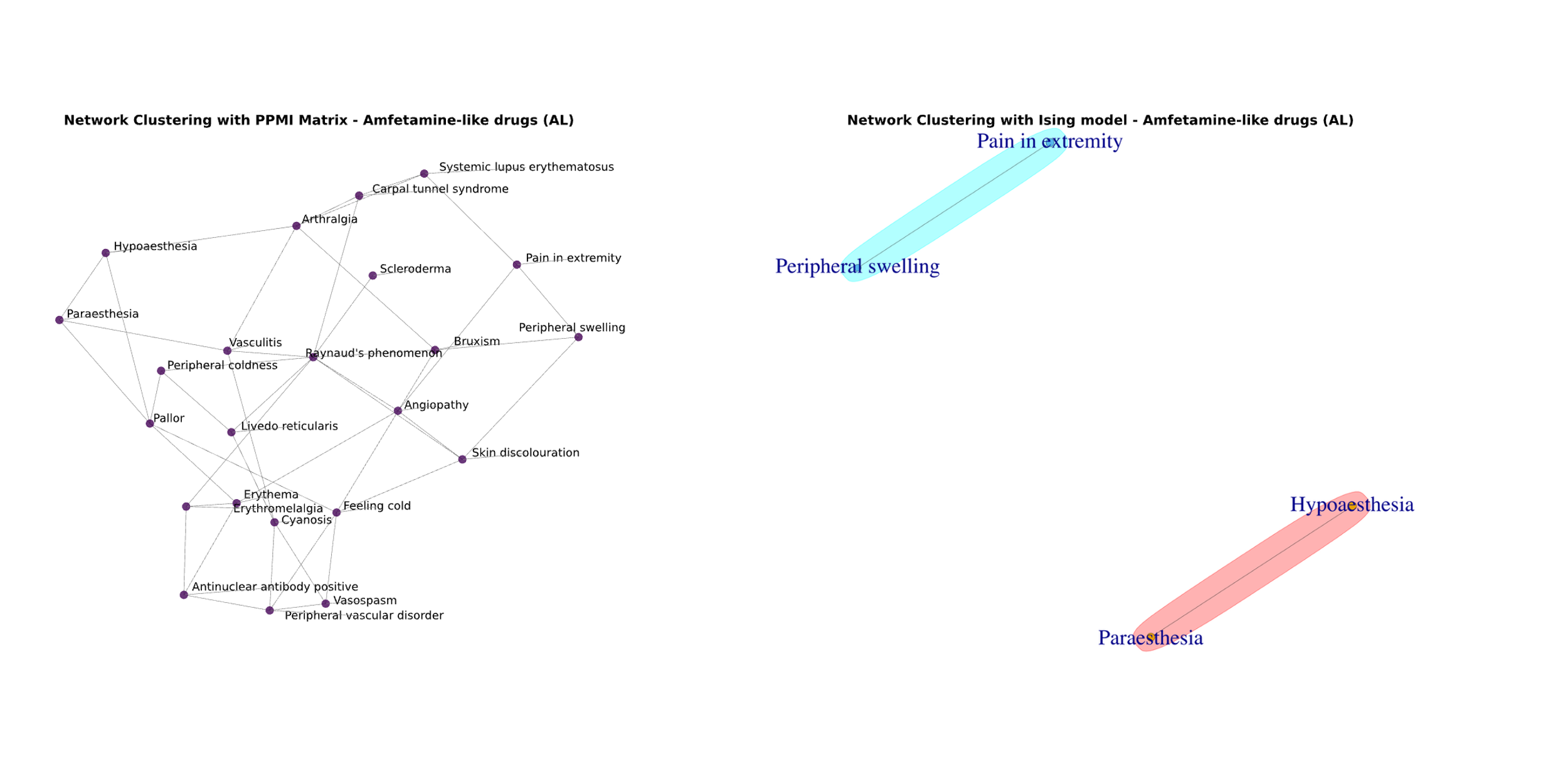


Figure 4 - Network clustering using PPMI matrix and Ising model for PTs associate with amfetamine like drugs (AL)


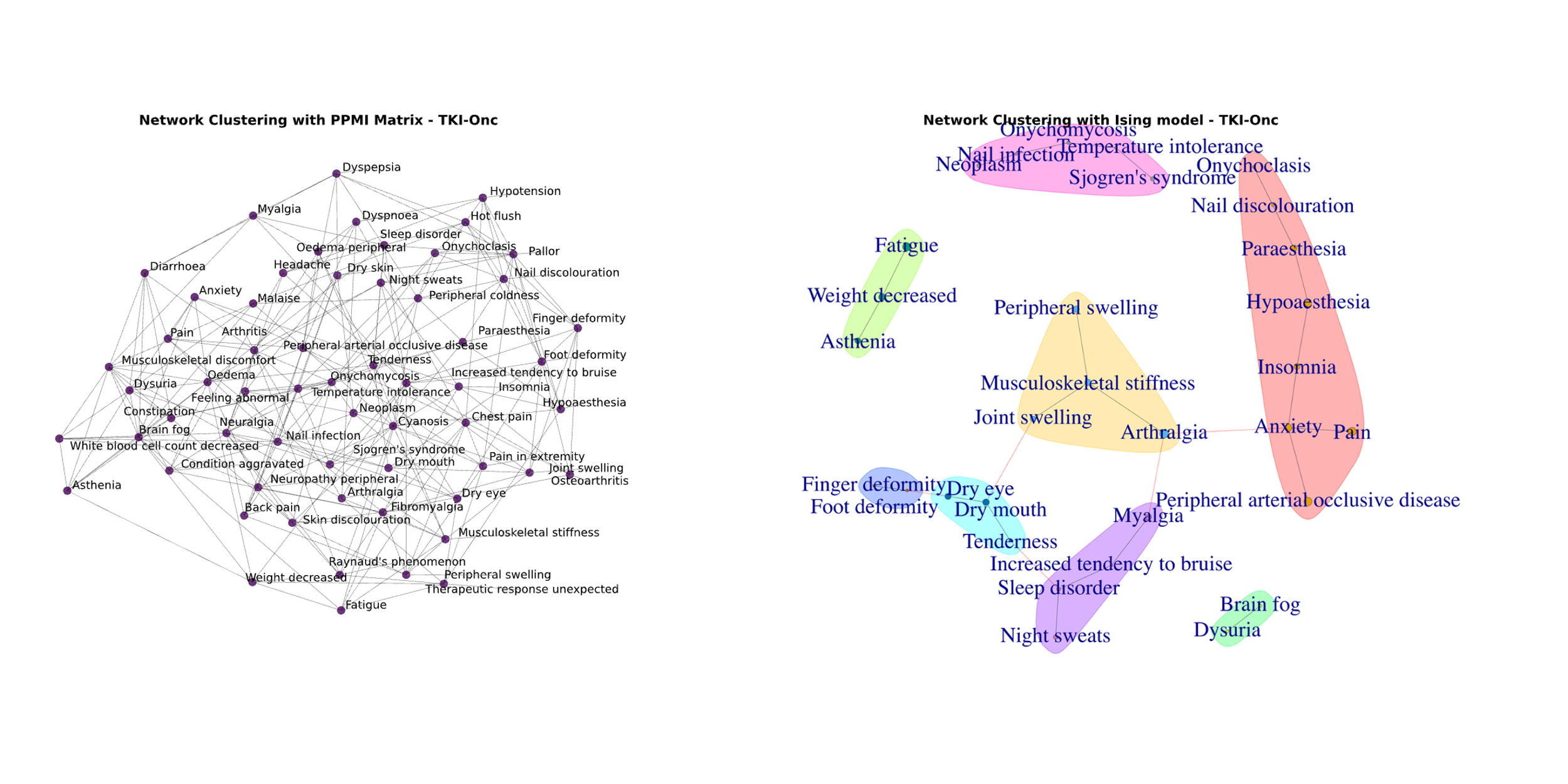


Figure 5 - Network clustering using PPMI matrix and Ising model for PTs associate with tyrosine kinase inhibitor used in oncology (TKI-Onc)
